# Multivariate gene expression analysis reveals functional connectivity changes between normal/tumoral prostates

**DOI:** 10.1186/1752-0509-2-106

**Published:** 2008-12-05

**Authors:** André Fujita, Luciana Rodrigues Gomes, João Ricardo Sato, Rui Yamaguchi, Carlos Eduardo Thomaz, Mari Cleide Sogayar, Satoru Miyano

**Affiliations:** 1 Human Genome Center, Institute of Medical Science, University of Tokyo, 4-6-1 Shirokanedai, Minato-ku, Tokyo, 108-8639, Japan; 2 Chemistry Institute, University of São Paulo, Av. Lineu Prestes, 748, São Paulo-SP, 05508-900, Brazil; 3 Mathematics, Computation and Cognition Center, Universidade Federal do ABC, Rua Santa Adélia, 166 – Santo André, 09210-170, Brazil; 4 Department of Electrical Engineering, Centro Universitário da FEI, Av. Humberto de Alencar Castelo Branco, 3972 – São Bernardo do Campo, 09850-901, Brazil

## Abstract

**Background:**

Prostate cancer is a leading cause of death in the male population, therefore, a comprehensive study about the genes and the molecular networks involved in the tumoral prostate process becomes necessary. In order to understand the biological process behind potential biomarkers, we have analyzed a set of 57 cDNA microarrays containing ~25,000 genes.

**Results:**

Principal Component Analysis (PCA) combined with the Maximum-entropy Linear Discriminant Analysis (MLDA) were applied in order to identify genes with the most discriminative information between normal and tumoral prostatic tissues. Data analysis was carried out using three different approaches, namely: (i) differences in gene expression levels between normal and tumoral conditions from an univariate point of view; (ii) in a multivariate fashion using MLDA; and (iii) with a dependence network approach. Our results show that malignant transformation in the prostatic tissue is more related to functional connectivity changes in their dependence networks than to differential gene expression. The MYLK, KLK2, KLK3, HAN11, LTF, CSRP1 and TGM4 genes presented significant changes in their functional connectivity between normal and tumoral conditions and were also classified as the top seven most informative genes for the prostate cancer genesis process by our discriminant analysis. Moreover, among the identified genes we found classically known biomarkers and genes which are closely related to tumoral prostate, such as KLK3 and KLK2 and several other potential ones.

**Conclusion:**

We have demonstrated that changes in functional connectivity may be implicit in the biological process which renders some genes more informative to discriminate between normal and tumoral conditions. Using the proposed method, namely, MLDA, in order to analyze the multivariate characteristic of genes, it was possible to capture the changes in dependence networks which are related to cell transformation.

## Background

Cancer is one of the main public health problems in the United States and worldwide [1]. Among the diverse types of neoplasia, prostate cancer is the third most common cancer in the World [2], being ranked as the second leading cause of death in men, the first being lung cancer [1]. Its incidence and mortality varies in different parts of the World, being highest in Western countries, mainly among Africans [3].

With the widespread use of the prostate-specific antigen (PSA) test, more men are examined, and consequently, identification of patients with asymptomatic low-stage tumors has increased considerably [4,5]. Although the majority of prostate cancers is confined to the prostate gland, rarely affecting life expectancy, in about 30% of the cases, a specialized group of cells from the primary tumor mass may invade and colonize other distant tissues causing death, therefore, metastatic disease rather than the primary tumor itself is responsible for death, causing the prognosis to be directly related to the spread of the tumor. Unfortunately, the therapeutic approaches used nowadays against advanced stages of prostatic cancers are not effective [6]. Therefore, it is extremely important to understand the basic molecular biology involved in this disease in order to prevent the progression of the tumor [6]. However, the identification and analysis of these molecular mechanisms has been hampered by the heterogeneity and high molecular complexity of the process involved in the development of this disease.

In the last few years, several efforts have been made towards determining the genetic mechanisms involved in the development of this tumor [6,7]. A widely used approach in studying the development of several types of cancers has been the high-throughput gene expression microarray analysis, which has provided a wealth of information about tumor marker genes. Conventional methods of microarray data analysis have been systematically used to examine the differentially expressed genes [8], and molecular pathways [9] and discriminative methods have been used in order to identify biomarkers [10,11].

In general, discriminant studies focus only on the classification accuracy of the method and on a pre-step selection of the features (genes) which best classifies the samples [12]. This selection of features is often carried out by selecting a subgroup of the most differentially expressed genes [13] or in a multivariate fashion [12]. However, understanding of the structure responsible for regulation of these discriminative set of genes in prostatic cancer is required [14].

Many years of intensive research have demonstrated that signaling molecules are organized into complex biochemical networks. These signaling circuits are complicated systems consisting of multiple elements interacting in a multifarious fashion. Signaling networks are regulated both in time and space [15]; allow the cell to decide which cellular process (cell division, differentiation, transformation, or apoptosis) is the most appropriate response for each situation. Due to the high connectivity and complexity of these biological systems, small modifications in a few members ("hub" genes, i.e., highly functionally connected genes) of these biochemical networks are sufficient to perturb the whole system [16], consequently resulting in a change on the cell's phenotype [17]. Frequently, changes in the relative concentration of molecules, such as mRNAs and proteins, are the unique parameter analyzed in biological systems. However, the biomolecules' concentration is not the only important variable, but their compartmentalization and diffusion are also determinants of the cell's phenotype. Therefore, these approaches are reductionists in defining a good biomarker as the most differentially expressed gene or protein when comparing distinct cellular contexts.

**Figure 1:**
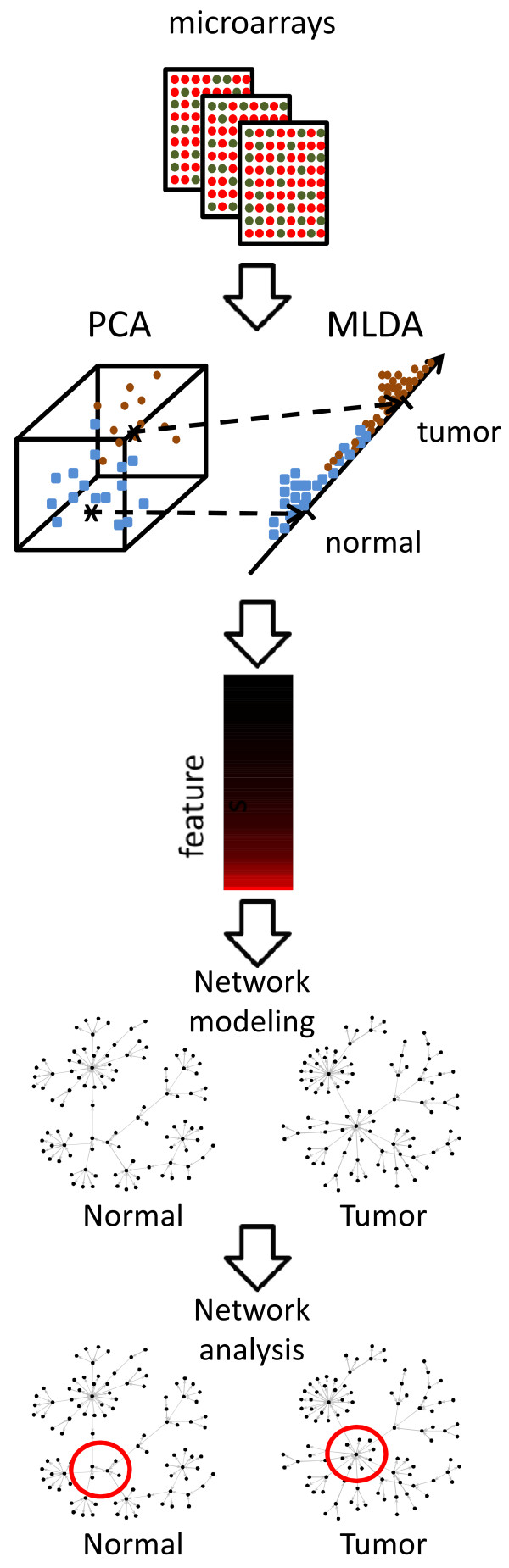
A pictorial scheme of the combination of PCA+MLDA and dependence network analysis for two populations (normal and tumoral prostatic tissues).

Here, we report a cDNA microarray-based study in prostatic cancer aimed at understanding why some genes are good predictors in discriminating normal versus tumoral samples and others are not. We demonstrate that the discriminative information between normal and tumoral prostates is related to the change in functional connectivity between certain genes and not necessarily in their differential expression, as has often been assumed. Moreover, we present a systematic and straightforward approach based on MLDA (Maximum-entropy Linear Discriminant Analysis) to identify putative biomarkers in high dimensional data (when the number of features is greater than the number of observations), and a dependence network analysis in order to interprete sets of discriminative genes. This idea is illustrated in Figure 1.

## Results

### Simulation

**Figure 2:**
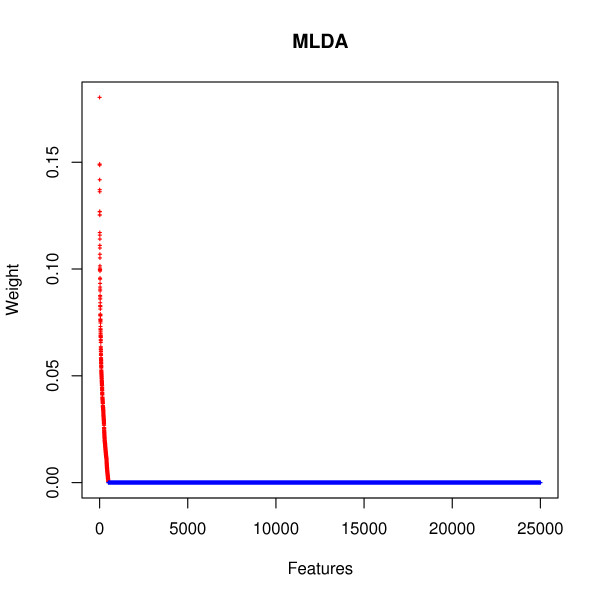
**The discriminative weight of each simulated feature.** The features are sorted (in decreasing order) by the absolute value of the weight. Red crosses represent the 500 features that have their functional connectivities alterated between conditions 1 and 2. Blue crosses represent the 24,500 features which have their functional connectivities unaltered.

The combination of PCA (Principal Component Analysis) + MLDA (Maximum-entropy Linear Discriminant Analysis) [18] was applied in a simulated data described in the Methods section in order to demonstrate that functional connectivity changes may be captured by the proposed approach. Figure 2 describes the weights in absolute values attributed by MLDA to each feature (artifically generated genes). The features are sorted in a decreasing order of weight. Red crosses represent the genes which have their functional connectivity alterated between conditions 1 and 2. Blue crosses represent the genes which have their connectivities unaltered.

### Samples classification

Applying the PCA combined with the MLDA approach to all ~25,000 genes available in our microarray dataset [19], it was possible to classify the samples with an accuracy of 96.5% (a misclassification of 2 out of 57 samples), using a leave-one-out cross validation.

### Projection matrix *ψ*_MLDA _analysis

**Figure 3:**
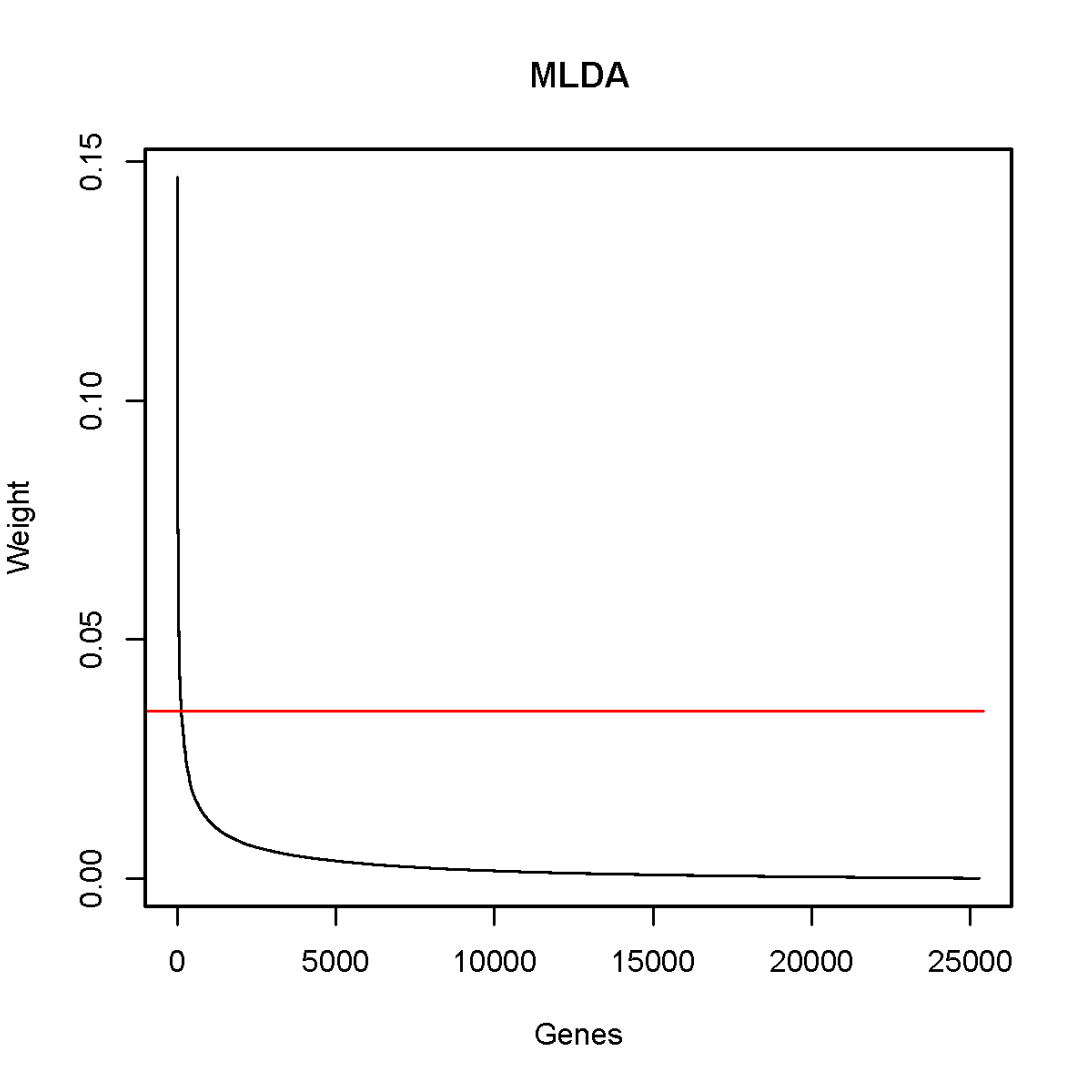
**The discriminative weight of each gene.** The genes are sorted (in decreasing order) by the absolute value of the weight. The horizontal red line indicates the 100th gene.

The projection matrix *ψ*_MLDA _contains the weights (degree of relationship between the gene and the normal/tumoral state) for each feature (gene). Figure 3 describes the weights in absolute values attributed by MLDA to each gene. The genes are sorted in a decreasing order of weight.

### The most informative genes correlated to prostatic cancer

Table 1 illustrates the top 100 features identified as the most informative genes related to malignant transformation by the PCA+MLDA approach ranked in a decreasing order of weight values. This set of 100 most informative genes represents ~0.4% of the total number of genes available in the microarrays (~25,000 genes). Notice that these 100 genes have a MLDA weight different from zero, i.e., the 100th gene RPS28 has a MLDA weight (~0.035, Table 1) located before the convergence of the curve to zero (Figure 3, the horizontal red line indicates the 100th gene). In order to verify the stability and robustness of our results, 27 observations out of 32 from normal sample and 20 out of 25 from tumoral sample were randomly selected and the *ψ*_MLDA _was re-calculated. This step was performed 100 times and the mean rank for each gene was obtained. About 80% of the originally obtained top 100 most discriminative genes were ranked as the top 100 most discriminative genes.

**Table 1:** *ψ*_MLDA_: the weights attributed by MLDA.

	Gene name	Official Full Name	*ψ*_MLDA_	p-value (Wilcoxon)	References:
1	*MYLK	myosin light chain kinase	0.14672	0.00000	[24]
2	*KLK2	kallikrein-related peptidase 2	0.12512	0.01053	[49]
3	*KLK3	kallikrein-related peptidase 3	0.12032	0.05625	[50]
4	HAN11	WD repeat domain 68	0.12019	0.00000	
5	*LTF	lactotransferrin	0.11594	0.00092	[39]
6	CSRP1	cysteine and glycine-rich protein 1	0.11355	0.00000	[51]
7	*TGM4	transglutaminase 4 (prostate)	0.10452	0.06063	[42]
8	*ACTG2	actin gamma 2 smooth muscle enteric	0.09826	0.00000	[52]
9	MYL6	myosin light chain 6 alkali smooth muscle and non-muscle	0.09817	0.00045	[53]
10	*RDH11	retinol dehydrogenase 11 (all-trans/9-cis/11-cis)	0.09583	0.00018	[54]
11	*AZGP1	alpha-2-glycoprotein 1 zinc-binding	0.08817	0.00059	[55]
12	NPAL3	NIPA-like domain containing 3	0.08478	0.00008	
13	**PRO1073**	PRO1073 protein	0.08077	0.28733	
14	*FXYD3	FXYD domain containing ion transport regulator 3	0.08024	0.05417	[56]
15	TPM2	tropomyosin 2 (beta)	0.07919	0.00001	[57]
16	CRYAB	crystallin alpha B	0.07560	0.00000	[58]
17	ACTA2	actin alpha 2 smooth muscle aorta	0.07372	0.01610	[59]
18	*RPS6	ribosomal protein S6	0.07323	0.12130	[60]
19	TMEM130	transmembrane protein 130	0.07296	0.00005	
20	*ACPP	acid phosphatase prostate	0.07185	0.00037	[61]
21	*PCP4	Purkinje cell protein 4	0.07128	0.00000	[62]
22	*SYNPO2	synaptopodin 2	0.06943	0.00000	[63]
23	*SORBS1	sorbin and SH3 domain containing 1	0.06773	0.00000	[64]
24	*MSMB	microseminoprotein beta	0.06588	0.00076	[65]
25	ACTC	actin alpha cardiac muscle 1	0.06335	0.00001	
26	*TGFB3	transforming growth factor beta 3	0.06313	0.00000	[66]
27	*MALT1	mucosa associated lymphoid tissue lymphoma translocation gene 1	0.06205	0.14208	[67]
28	ZNF532	zinc finger protein 532	0.06131	0.00000	
29	ANXA1	annexin A1	0.06119	0.00001	[68]
30	PALLD	palladin cytoskeletal associated protein	0.06116	0.00000	[69]
31	*MT2A	metallothionein 2A	0.06054	0.00141	[70]
32	ING5	inhibitor of growth family member 5	0.05872	0.93009	[71]
33	PGM5	phosphoglucomutase 5	0.05862	0.00000	
34	**SERPINA3**	serpin peptidase inhibitor clade A (alpha-1 antiproteinase antitrypsin) member 3	0.05828	0.19710	[72]
35	*KRT5	keratin 5 (epidermolysis bullosa simplex Dowling-Meara/Kobner/Weber-Cockayne types)	0.05699	0.00000	[73]
36	**RPL5**	ribosomal protein L5	0.05589	0.53873	[74]
37	*IGF1	insulin-like growth factor 1 (somatomedin C)	0.05549	0.00000	[75]
38	**ZNF92**	zinc finger protein 92 (HTF12)	0.05388	0.16056	
39	*FOLH1	folate hydrolase (prostate-specific membrane antigen) 1	0.05361	0.08683	[76]
40	*CYR61	cysteine-rich angiogenic inducer 61	0.05318	0.00020	[77]
41	FHL1	four and a half LIM domains 1	0.05305	0.00000	[78]
42	*H19	H19 imprinted maternally expressed transcript	0.05221	0.00006	[79]
43	DMN	desmuslin	0.05219	0.00000	
44	NEFH	neurofilament heavy polypeptide 200 kDa	0.05186	0.00001	[80]
45	PPP1R12B	protein phosphatase 1 regulatory (inhibitor) subunit 12B	0.05149	0.00000	
46	ANTXR2	anthrax toxin receptor 2	0.05141	0.00002	[81]
47	MRLC2	myosin regulatory light chain MRLC2	0.05056	0.02204	[82]
48	C20orf103	chromosome 20 open reading frame 103	0.05055	0.00150	
49	UBA52	ubiquitin A-52 residue ribosomal protein fusion product 1	0.05033	0.00518	[83]
50	TRGV9	T cell receptor gamma variable 9	0.04983	0.00190	
51	*SPARC	secreted protein acidic cysteine-rich (osteonectin)	0.04969	0.00240	[84]
52	*AMACR	alpha-methylacyl-CoA racemase	0.04903	0.00011	[85]
53	**DNER**	delta/notch-like EGF repeat containing	0.04809	0.09301	[86]
54	PRNP	prion protein (p27-30)	0.04806	0.00000	[87]
55	PDK4	pyruvate dehydrogenase kinase isozyme 4	0.04751	0.00002	[88]
56	*APOD	apolipoprotein D	0.04744	0.12931	[89]
57	*HERPUD1	homocysteine-inducible endoplasmic reticulum stress-inducible ubiquitin-like domain member 1	0.04695	0.00001	[90]
58	FSTL1	follistatin-like 1	0.04692	0.00092	[91]
59	**HSPCB**	heat shock protein 90 kDa alpha (cytosolic) class B member 1	0.04663	0.08386	[92]
60	*GSTM2	glutathione S-transferase M2 (muscle)	0.04446	0.00000	[93]
61	*PTN	pleiotrophin	0.04440	0.00000	[94]
62	*ERG	v-ets erythroblastosis virus E26 oncogene homolog (avian)	0.04410	0.06528	[95]
63	*CTGF	connective tissue growth factor	0.04342	0.00004	[96]
64	*GUCY1A3	guanylate cyclase 1 soluble alpha 3	0.04303	0.05841	[97]
65	MT1F	metallothionein 1F	0.04303	0.00002	[98]
66	*TIMP3	TIMP metallopeptidase inhibitor 3	0.04225	0.00000	[99]
67	*LDHB	lactate dehydrogenase B	0.04217	0.00000	[100]
68	RNASE4	ribonuclease RNase A family 4	0.04167	0.00000	
69	ANPEP	alanyl aminopeptidase	0.04165	0.00002	[101]
70	*CAV1	caveolin 1 caveolae protein 22 kDa	0.04135	0.00000	[102]
71	TM9SF2	transmembrane 9 superfamily member 2	0.04122	0.01275	
72	*HSPB8	heat shock 22 kDa protein 8	0.04088	0.00000	[103]
73	TUBA1A	tubulin alpha 1a	0.04087	0.00018	
74	**PDLIM5**	PDZ and LIM domain 5	0.04077	0.32533	[104]
75	LPP	LIM domain containing preferred translocation partner in lipoma	0.04073	0.00003	[105]
76	**MAD2L1BP**	MAD2L1 binding protein	0.04051	0.62639	[106]
77	*ADAMTS1	ADAM metallopeptidase with thrombospondin type 1 motif 1	0.04048	0.00011	[107]
78	*RHOA	ras homolog gene family member A	0.04039	0.11368	[108]
79	*TXNIP	thioredoxin interacting protein	0.03995	0.00227	[109]
80	**OGDH**	oxoglutarate (alpha-ketoglutarate) dehydrogenase (lipoamide)	0.03974	0.07543	
81	**RPL35**	ribosomal protein L35	0.03971	0.17555	
82	*ANKH	ankylosis progressive homolog (mouse)	0.03856	0.00318	[110]
83	MPST	mercaptopyruvate sulfurtransferase	0.03856	0.00000	[111]
84	MORF4L2	mortality factor 4 like 2	0.03831	0.01337	[112]
85	CRISPLD2	cysteine-rich secretory protein LCCL domain containing 2	0.03799	0.00000	
86	*CD9	CD9 molecule	0.03787	0.00150	[113]
87	ALDH3A2	aldehyde dehydrogenase 3 family member A2	0.03696	0.00001	
88	SCN2B	sodium channel voltage-gated type II beta	0.03693	0.00024	[114]
89	*SPARCL1	SPARC-like 1 (mast9 hevin)	0.03693	0.00045	[115]
90	IGJ	immunoglobulin J polypeptide linker protein for immunoglobulin alpha and mu polypeptides	0.03683	0.00190	[116]
91	ZNF134	zinc finger protein 134	0.03670	0.00007	
92	**MRPL43**	mitochondrial ribosomal protein L43	0.03655	0.54934	
93	LOC152485	hypothetical protein LOC152485	0.03647	0.00000	
94	**CALM2**	calmodulin 2 (phosphorylase kinase delta)	0.03622	0.05417	[117]
95	COL9A2	collagen type IX alpha 2	0.03546	0.00141	
96	*PAGE4	P antigen family member 4 (prostate associated)	0.03541	0.00001	[118]
97	CALM1	calmodulin 1 (phosphorylase kinase delta)	0.03536	0.00098	[119]
98	*ACTB	actin beta	0.03508	0.01159	[120]
99	*AGR2	anterior gradient homolog 2 (Xenopus laevis)	0.03498	0.56006	[121]
100	**RPS28**	ribosomal protein S28	0.03497	0.15578	

*: genes already described to be related to prostatic cancer. In bold are the genes which do not present statistical evidences to be differentially expressed between normal and tumoral conditions.

We have also manually annotated (which we believe be more accurate than automatic computer-based annotation, since it may be more efficient to capture semantic information from published articles) this set of 100 genes [see Table 1 and Additional file 1].

### Putative differentially expressed genes

We have also searched for differentially expressed genes. About 25% of the genes listed in Table 1 do not present statistical evidence to be differentially expressed between normal and tumoral conditions.

#### Relevance networks

**Figure 4:**
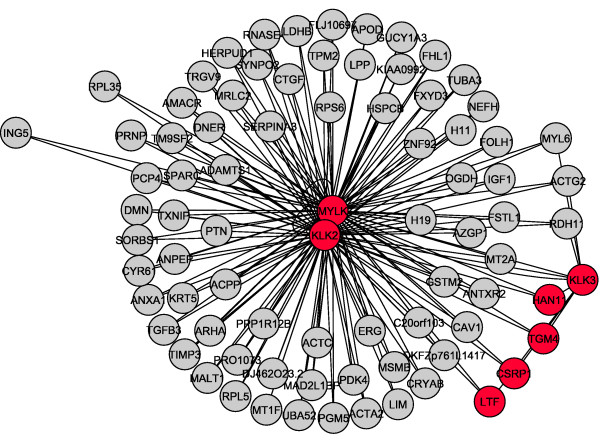
**A normal prostate relevance network constructed with the top 100 most discriminative genes and FDR of 5%.** Core genes are represented in red.

**Figure 5:**
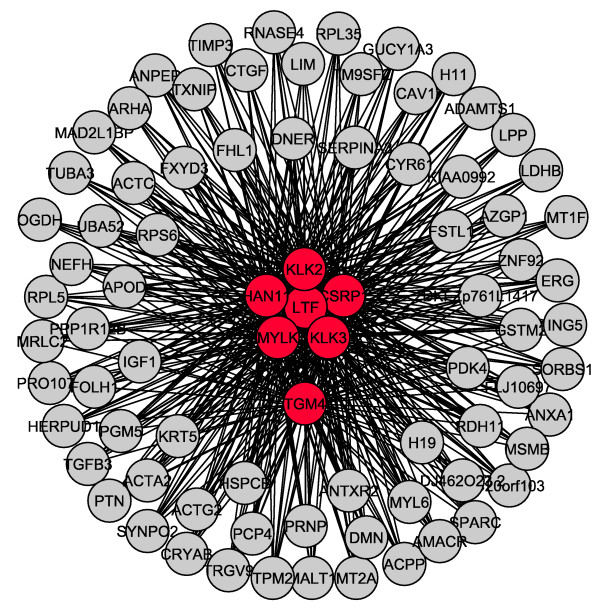
**A tumoral prostate relevance network constructed with the top 100 most discriminative genes and FDR of 5%.** Core genes are represented in red.

Both normal and tumoral relevance networks with the top 100 most informative genes were constructed, considering a false discovery rate of 5%, being illustrated in Figures 4 and 5, respectively. Nodes in red are the genes which have their functional connectivity (estimated using the non-parametric Hoeffding's D measure [20]) changed considerably between normal versus tumoral conditions, i.e., they become "hubs" (highly connected genes) [16] in tumoral prostates. "Hub" genes were maintained also when relevance networks were constructed under different FDR thresholds (1, 5 and 10%).

## Discussion

Firstly, the PCA+MLDA approach was applied to a simulated data set in order to illustrate that differences in connectivity may be behind the oncogenesis process. Sato *et al*. (2008) [21] have already demonstrated in another context (neuroscience) that the information contained in the connectivity may be useful to sample classification. The simulation was performed in a large scale multidimensional condition, where the relevant features (genes which have the connectivity changed) are only 2% (500 out of 25,000 genes). Interestinlgy, MLDA was able to correctly identify the discriminative features, represented by red crosses in Figure 2. Notice that the relevant features for discrimination do not present differential expression between conditions 1 and 2 (by construction).

In order to verify whether gene expression data contain the information to discriminate normal from tumoral prostatic samples, we have applied the PCA+MLDA approach to actual biological data, obtaining a high classification accuracy (96.5%) by the leave-one-out cross-validation. In this case, we have used all the principal components in order to avoid losing information. PCA is applied regarding computational cost and memory limitation. It is important to mention that the numerical results are identical in the absence of the PCA step [22]. Notice that MLDA does not require a pre-step feature selection, because it may also work for high dimensional data. Therefore, it was possible to include all of the 25,000 genes of the microarray dataset.

Since it was possible to verify that gene expression data retains information for classification, we analyzed the *ψ*_MLDA _projection matrix which contains the weight values for each feature (gene). Notice that the majority of the genes shown in Figure 3 have weights near zero, and only a few genes actually have discriminative information (high weight).

By analyzing Table 1, it is possible to verify that most of the 100 informative genes had already been described in the literature as genes related to cancer (76 genes) and 45 genes had specifically been associated to prostate tumor. Interestingly, most of the other 24 genes do not have references describing their functionality. Therefore, they may be associated to cancer but have not been studied yet. The description of the 76 genes in the literature corroborates the results obtained by the PCA+MLDA method, indicating that these genes are informative to discriminate between normal and tumoral samples. The stability and robustnees of this result were verified by obtaining around 80% of the same top 100 genes when five observations were excluded randomly from normal sample and five from tumoral sample in 100 re-calculations. For more details about annotation of the top 100 genes and the complete list of the ~25,000 genes, please see Additional file 2.

Comparing the weights obtained by MLDA and the differentially expressed genes, it is surprising that the most differentially expressed genes are not necessarily the most discriminative ones. In other words, a multivariate combination of genes may be regulating the normal/tumoral state, i.e., the combination of genes may contain more information about normal/tumoral conditions than an univariate differentially expressed gene.

Since it is known that a complex network is involved in the regulation of several molecular processes, we further analyzed the dependence network involved in these putative biomarkers in order to gain new insights. The analyis of Figures 4 and 5 indicate that exactly the top seven most discriminative genes described in Table 1 (MYLK, KLK2, KLK3, HAN11, LTF, CSRP1, TGM4) have considerably changed their functional connectivity between normal and tumoral conditions as illustrated by red nodes in Figures 4 and 5. These seven genes become "hubs" [16], i.e., highly connected genes in the tumoral condition, whereas in the normal condition, their connectivity was not different when compared to that of other genes. Furthermore, these seven genes maintained the position of the top seven most discriminative ones also when we have re-sampled the samples (the experiment which was performed in order to verify the stability and robustness of the top 100 genes). A Z-value summary table related to these seven genes is illustrated in Table 2. Z-values increase from normal to tumoral conditions, representing the changes in functional connectivities between these two conditions. The mean Z-values were calculated between the "hub" gene and the other 99 genes. In addition, in the list of the most discriminative features, there are genes which are more differentially expressed than these seven ones (lower p-value), however, their connectivity did not change. Krostka and Spang (2004) [17] have already suggested that differences in co-regulation between normal/disease states may be related to some pathologies. Moreover, Sato *et al*. (2008) [21] have reported that changes in networks connectivities may influence classification methods. These reports support our results showing that changes in functional connectivity may be closely related to the normal/tumoral states in prostate and that these changes in dependence may contain an additional information when compared to differential gene expression.

**Table 2:** The seven "hub" genes.

Gene name	mean Z-value (normal)	Standard Error	mean Z-value (tumoral)	Standard Error
MYLK	1.138	0.107	2.464	0.177
KLK2	0.871	0.084	1.161	0.102
KLK3	1.070	0.100	0.953	0.073
HAN11	1.305	0.142	1.502	0.141
LTF	0.862	0.080	1.750	0.127
CSRPP1	1.254	0.139	1.601	0.157
TGM4	0.869	0.116	0.956	0.121

Mean Z-values obtained by Hoeffding's D measure and the corresponding standard errors.

Almost all top seven genes identified as the most discriminative features between normal and tumoral phenotypes had previously been described in the literature as being associated to cancer. The only gene that so far has not been correlated to cancer is HAN11, probably because little is known about this gene (only two articles were found in the literature describing this gene). Five of these top seven genes namely, MYLK, KLK2, KLK3, LTF and TGM4 had already been specifically related to prostate carcinoma (Table 1).

Myosin light chain kinase (MYLK) is one of them. This enzyme catalyzes the phosphorylation of a specific serine residue on the 20 kD light chain of myosin II (MCL20), consequently regulating the actin-myosin II interaction [23]. This reaction is responsible for smoothing muscle contraction/relaxation and organization of the cytoskeleton. Due to the central role played by the cytoskeleton in cell division and motility, it has been demonstrated that MYLK inhibition induces apoptosis in mammary prostate cancer cells and inhibits the growth of mammary and prostate tumors in rats and mice [24]. Furthermore, since MLC20 phosphorylation is necessary for cell motility [25,26], MYLK inhibition blocks cancer cell invasion and adhesion *in vitro*. As a result, some reports described the use of MYLK inhibitors as anti-cancer agents since they prevent cancer cells migration [27,28].

KLK3, also known as prostate specific antigen (PSA), is another gene which presents high functional connectivity in tumoral samples. PSA is a serine protease, secreted into seminal plasma, belonging to the human kallikrein gene family, being responsible for semen liquefaction. It is the first FDA (Food and Drug Administration)-approved tumor marker for cancer detection [29]. The prostatic gland volume affects the PSA level in serum, because it is produced and secreted by prostatic tissue [30,31]. However, increased levels of KLK3 are also observed in some patients with benign prostate hyperplasia. Therefore, elevated PSA concentration in patients' plasma may be indicative not only of prostate cancer, but, also of other prostatic pathologies. Consequently, the use of PSA as a cancer-specific marker is questioned.

Nowadays, 15 members of the kallikrein family (KLKs) are described in humans [32]. Among the KLKs, the highest homology is found between PSA and KLK2. In this case, the identity is 78% and 80% at the amino acid and DNA level, respectively [33]. KLK2 is another gene that presented functional connectivity changes between normal/tumoral conditions. The ratio of KLK2 to free PSA improves the discrimination of benign prostate hyperplasia and prostate cancer patients [34]. In addition, it has already been described that KLK2 discriminates between high and low grade tumors [35]. There is evidence indicating that KLK2 is more closely correlated to the total volume and higher grade prostate cancers than PSA [36].

Identification of both of these classic biomarkers of prostate carcinomas (PSA and KLK2), in our list of the most informative genes, provides additional evidence to the hypothesis that functional connectivity changes and not only differential expression levels are highly correlated to normal/tumoral process.

Another gene classified as one of the most discriminative prostate cancer biomarkers, whose anti-tumorigenic role has already been described [37] is lactotransferrin (LTF). This non-heme iron-binding glycoprotein [38] is found in a variety of biological secretions, such as semen, as well as in several secretions derived from glandular epithelium cells, including the prostate. LTF mRNA and protein levels are downregulated in prostate cancer, with significant PSA recurrence associations, due to promoter silencing by hypermethylation [39]. It has been reported that bovine lactotransferrin significantly inhibits colon, esophagus, lung, bladder and liver cancers in rats [40]. Prostate cancer cells treated with LTF presented high apoptotic response, growth arrest at G1 and reduced S phase, suggesting a role for specific cell cycle regulatory mechanisms in LTF-mediated cell growth inhibition [39].

CSRP1 (cysteine and glycine-rich protein 1) and TGM4 (human prostate-specific transglutaminase gene) are two other genes that become "hubs" [16] along tumoral development. The former belongs to the CSRP family, encoding a group of LIM domain proteins, which may be involved in regulatory processes which are important for development and cellular differentiation. Hirasawa and collaborators (2006) [41] suggest the use of CSRP as an important biomarker of hepatocellular carcinoma malignancy, because CSRP1 is inactivated in this model by aberrant methylation [41]. The latter, TGM4 was described as a candidate biomarker of region-specific epithelial identity in the prostate [42], being involved in the formation of stable protein-protein or protein-polyamide bounds [43].

Therefore, the literature supports the suggestion that these top seven genes (except for HAN11) may be considered as the most closely and informative prostate cancer biomarkers. Consequently, this suggests that the malignant transformation process in prostatic tissue is more correlated to functional connectivity changes in the gene dependence networks than differential gene expression itself.

Almost all of the 100 genes identified by PCA+MLDA are correlated to cancer, and, in many cases, to prostate cancer. Thus, TIMP3 and ADAMTS1 (Table 1) are genes classically correlated to invasion and the metastatic process, the main cancer attributes responsible for death.

## Conclusion

In summary, our main goal using PCA+MLDA was not dimension reduction or verification of the classification accuracy, but to investigate the discriminative characteristics extracted from the whole microarray dataset and how one can interpret them, although this procedure may also be used for classification, yielding good results, as previously described.

We have demonstrated that changes in functional connectivity may underly the biological process which render some genes more informative to discriminate between normal and tumoral conditions. Using the proposed PCA+MLDA method in order to analyze the multivariate gene characteristic, it was possible to capture the changes in dependence networks which are related to cell transformation. Identification of seven genes (MYLK, KLK2, KLK3, HAN11, LTF, CSRP1, TGM4) which have their connectivity altered between normal/tumoral conditions may provide novel insights into specific targets against tumor progression.

## Methods

### Principal component analysis (PCA)

Principal component analysis is a dimension reduction technique used to reduce the high dimensional space (number of genes).

PCA is defined as linear transformations which maps the data to a new orthogonal coordinate system. These linear combinations are constructed so that the greatest variance by any projection lies on the first coordinate (called the first principal component), the second greatest variance on the second coordinate, and so on.

In other words, PCA summarizes the original features information by retaining characteristics of the dataset which most contribute to its variance.

For a gene expression data matrix **X **containing the genes in the columns and the observations in the rows (normalized to have zero mean and unit variance), the PCA transformation matrix *ψ*_PCA _is given by

(1) *ψ*_PCA _= *eigenvectors*(*cov*(**X**^T^))

where *cov *is the covariance matrix. In order to prevent losing any variance information, *ψ*_PCA _is composed of all eigenvalues with non-zero eigenvectors. Here, PCA is used only to reduce computational and memory costs.

### Maximum-entropy linear discriminant analysis (MLDA)

In gene expression data analysis, we usually have a large number of genes (features), but only a few number of observations, i.e., microarrays experiments.

A critical problem in applying conventional Linear Discriminant Analysis (LDA) to these types of data is the singularity and instability of the within-class scatter matrix calculated when the number of features approaches the number of available examples. In order to overcome this limitation, we applied the MLDA approach.

The MLDA method is concerned with the stabilization of pooled covariance matrix estimate **S**_**p**_. This covariance matrix **S**_**p **_is constructed by selecting the largest dispersions regarding the **S**_**p **_average eigenvalue. It is based on the maximum entropy covariance selection idea developed by Thomaz *et al *(2004) [18].

It is known that the estimated errors of small eigenvalues are greater than that of large eigenvalues. Therefore, Thomaz *et al*. (2007) [44] proposed to expand only the smaller and less reliable eigenvalues of **S**_**p**_, keeping most of the larger eigenvalues unchanged.

The algorithm may be described as follows:

1. Let the between-class scatter matrix **S**_**b **_be defined as

(2) $$S_{b}=\sum_{i=1}^{g}n_{i}({\bar{x}}_{i}-\bar{x}){\left({\bar{x}}_{i}-\bar{x}\right)}^{T}$$

and the within-class scatter matrix **S**_**w **_be defined as

(3) $$S_{w}=\sum_{i=1}^{g}(n_{i}-1)S_{i}=\sum_{i=1}^{g}\sum_{j=1}^{n_{i}}(x_{i,j}-{\bar{x}}_{i}){\left(x_{i,j}-{\bar{x}}_{i}\right)}^{T}$$

where **x**_*i*, *j *_is the *m*-dimensional (*m*: number of genes) observation *j *from class ∏_*i *_(*i *= 1, 2, where 1 = normal and 2 = tumoral in our case) containing the gene expressions in the rows, *n*_*i *_is the number of observations (microarrays) from class ∏_*i*_, and *g *is the total number of classes (*g *= 2 in our case).

The vector $\bar{x}$_*i *_is the unbiased sample mean and the matrix **S**_*i *_is the sample covariance matrix of class ∏_*i*_. The mean vector $\bar{x}$ is calculated by

(4) $$\bar{x}=\frac{1}{n}\sum_{i=1}^{g}n_{i}{\bar{x}}_{i}=\frac{1}{n}\sum_{i=1}^{g}\sum_{j=1}^{n_{i}}x_{i,j}$$

where *n *is the total number of microarrays, i.e., $n=\sum_{j=1}^{g}n_{j}$.

2. Calculate the *ψ *eigenvectors and Λ eigenvalues of **S**_**p**_, where **S**_**p **_= **S**_**w**_/[*n *- *g*].

3. Calculate $\bar{\lambda }$, i.e., the average eigenvalue

(5) $$\bar{\lambda }=\frac{1}{m}\sum_{j=1}^{m}\lambda_{j}=\frac{trace(S_{p})}{m}$$

4. Construct the new matrix of eigenvalues based on the following largest dispersion criterion Λ* = *diag *[*max*(*λ*_*i*_, $\bar{\lambda }$),..., *max*(*λ*_*m*_, $\bar{\lambda }$)]

5. Construct the modified within-class scatter matrix $S_{w}^{*}$

(6) $$S_{w}^{*}=S_{p}^{*}(n-g)=(\psi \Lambda^{*}\psi^{T})(n-g)$$

6. Finally, calculate the projection matrix *ψ*_MLDA _which maximizes the ratio of the determinant of the between-class scatter matrix to the determinant of the within-class scatter matrix (Fisher's criterion):

(7) $$\psi_{MLDA}=eigenvector(S_{w}^{*-1}S_{b})$$

The main advantage of MLDA is that it avoids both the singularity and instability of the within-class scatter matrix **S**_**w **_when applied directly to gene expression data, which consists of a low number of observations and a high number of features.

The implemented R code is available in the Additional file 3.

### Simulation

This simulation was designed in order to demonstrate that MLDA is capable to discriminate two different conditions and also to identify the intrinsic functional connectivity changes underlying the tumoral process. For this simulation, artificial gene expressions for 25,000 genes (features) were generated, based on the simulation illustrated in [21]. The 25,000 genes were divided in three sets A (250 genes), B (250 genes) and C (24,500 genes). For each gene, 30 observations representing "normal" condition and 30 observations representing "tumoral" conditions were generated. The model to investigate the situation where there are fuctional connectivity changes and there is no differences in gene expressions between conditions 1 and 2 were as follows:

*ϕ*^(*A*) ^= 1 + 0.3*ε*

$$\phi^{\left(B\right)}=\{\begin{array}{ll} 1.3\phi^{\left(A\right)}+0.3\epsilon & \text{if condition }1.\\ 0.9\phi^{\left(A\right)}+0.3\epsilon & \text{if condition }2. \end{array}$$

*gene*^(*A*) ^= *ϕ*^*A *^+ 0.3*θ*_*A*_

*gene*^(*B*) ^= *ϕ*^*B *^+ 0.5*θ*_*B*_

*gene*^(*C*) ^= *θ*_*C*_

where *ε*, *ϵ*, *θ*_*A*_, *θ*_*B *_and *θ*_*C *_are independent Gaussian random variables with mean of zero and variance of one. This model considers two latent variables *ϕ*^(*A*) ^and *ϕ*^(*B*)^. Moreover, there is a functional relationship between A and B. Notice that there is no difference in means between A and B.

### Differentially expressed genes

In order to identify putative differentially expressed genes, we have applied the non-parametric Wilcoxon test under a false discovery rate control (FDR) [45] of 5%. Wilcoxon procedure tests the median, therefore, it is more robust to outliers than the t-test (which tests the mean).

### Relevance networks

Relevance networks [46] were constructed using the Hoeffding's D measure [20], a non-parametric association method (the R code is freely available in the Hmisc package at [47]), which is more robust to outliers than the Pearson's correlation. Pairwise correlations were measured and the false discovery rate (FDR) [45] was controlled to 1, 5 and 10%. "Hub" genes were determined by calculating the degree (the number of adjacent edges, i.e. functional connectivities) of each gene and selecting the highest ones.

### Microarrays

We have analyzed the normal and tumoral prostate dataset publicly available at the Stanford MicroArray Database [48,19]. This dataset is composed of ~25,000 genes with 32 observations for normal state and 25 for tumoral condition.

## Authors' contributions

AF has made substantial contributions to the conception, design and implementation of the study, and has also been responsible for drafting the manuscript. LRG has made substantial contributions to the biological interpretations, and has been responsible for drafting some parts of the manuscript. JRS has made substantial contributions to data analysis and applications of statistical concepts. RY, CET and MCS have discussed the results and critically revised the manuscript for important intellectual content. SM has directed the work and has given the final approval of the version to be published.

## Supplementary Material

Additional file 1 **Manual annotation.** The manual annotation of the 100 genes described in Table 1. Click here for file

Additional file 2 **MLDA hyperplane weight.** The MLDA hyperplane weight and the p-values (Wilcoxon test) for all the ~25,000 genes. Click here for file

Additional file 3 **R code.** Implemented R code for MLDA. Click here for file
