# Supplementary material for: Multivariate gene expression analysis reveals functional connectivity changes between normal/tumoral prostates
Source: BMC Syst Biol. 2008 Dec 5;2:106. doi: 10.1186/1752-0509-2-106 (PMC2628381; doi:10.1186/1752-0509-2-106)
Supplement: Additional file 1 — Manual annotation. The manual annotation of the 100 genes described in Table 1. [file 1752-0509-2-106-S1.doc]

**Gene 1: MYLK**

**Official symbol:** MYLY

**Official Full Name:** myosin light chain kinase

**Also known as:** KRP; MLCK; MYLK1; smMLCK; MLCK108; MLCK210; MSTP083; FLJ12216; DKFZp686I10125

**Function:** Kinase protein involved in myosin interaction with actin
filaments to produce contractile activity.

**Cancer Correlation:** apoptosis, Tumor invasion, migration and metastasis.

**References:**

1. [Gu LZ, Hu WY, Antic N, Mehta R, Turner JR, de Lanerolle P.](http://www.ncbi.nlm.nih.gov/pubmed/16574402?ordinalpos=5&itool=EntrezSystem2.PEntrez.Pubmed.Pubmed_ResultsPanel.Pubmed_RVDocSum) (2006). Inhibiting myosin light chain kinase retards the growth of mammary and prostate cancer cells. *European Journal of Cancer* **42**: 948-957.

2. [Fazal F, Gu L, Ihnatovych I, Han Y, Hu W, Antic N, Carreira F, Blomquist JF, Hope TJ, Ucker DS, de Lanerolle P.](http://www.ncbi.nlm.nih.gov/pubmed/15988034?ordinalpos=8&itool=EntrezSystem2.PEntrez.Pubmed.Pubmed_ResultsPanel.Pubmed_RVDocSum) (2005). Inhibiting myosin light chain kinase induces apoptosis in vitro and in vivo. *Molecular and cellular Biology* **25**: 6259-6266.

3. Tohtong R, Phattarasakul K, Jiraviriyakul A, Sutthiphongchai T. (2003). Dependence of metastatic cancer cell invasion on MLCK-catalyzed phosphorylation of myosin regulatory light chain. *Prostate Cancer and Prostatic Diseases* **6**: 212-216.

4. [Nguyen DH, Catling AD, Webb DJ, Sankovic M, Walker LA, Somlyo AV, Weber MJ, Gonias SL.](http://www.ncbi.nlm.nih.gov/pubmed/10402467?ordinalpos=28&itool=EntrezSystem2.PEntrez.Pubmed.Pubmed_ResultsPanel.Pubmed_RVDocSum) (1999). Myosin light chain kinase functions downstream of Ras/ERK to promote migration of urokinase-type plasminogen activator-stimulated cells in an integrin-selective manner. *Journal of Cell Biology* **146**: 149-164.

**Gene 2: KLK2**

**Official symbol:** KLK2

**Official Full Name:** kallikrein-related peptidase 2

**Also known as:** hK2; KLK2A2; MGC12201

**Function: S**erine protease highly abundant in the prostate and found in seminal plasma.

**Cancer Correlation:** Invasion, tumor spread, metastasis and growth

**References:**

5. [Lilja H, Ulmert D, Björk T, Becker C, Serio AM, Nilsson JA, Abrahamsson PA, Vickers AJ, Berglund G.](http://www.ncbi.nlm.nih.gov/pubmed/17264339?ordinalpos=6&itool=EntrezSystem2.PEntrez.Pubmed.Pubmed_ResultsPanel.Pubmed_RVDocSum) (2007). Long-term prediction of prostate cancer up to 25 years before diagnosis of prostate cancer using prostate kallikreins measured at age 44 to 50 years. *Journal of Clinical Oncology* **25**: 431-436.

6. [Nam RK, Zhang WW, Klotz LH, Trachtenberg J, Jewett MA, Sweet J, Toi A, Teahan S, Venkateswaran V, Sugar L, Loblaw A, Siminovitch K, Narod SA.](http://www.ncbi.nlm.nih.gov/pubmed/17085659?ordinalpos=10&itool=EntrezSystem2.PEntrez.Pubmed.Pubmed_ResultsPanel.Pubmed_RVDocSum) (2006). Variants of the hK2 protein gene (KLK2) are associated with serum hK2 levels and predict the presence of prostate cancer at biopsy. *Clinical Cancer Research* **12**:6452-6458.

7. [Chiang CH, Hong CJ, Chang YH, Chang LS, Chen KK.](http://www.ncbi.nlm.nih.gov/pubmed/15643194?ordinalpos=7&itool=EntrezSystem2.PEntrez.Pubmed.Pubmed_ResultsPanel.Pubmed_RVDocSum) (2005). Human kallikrein-2 gene polymorphism is associated with the occurrence of prostate cancer. *Journal of Urology* 173: 429-432.

8. [Réhault S](http://www.ncbi.nlm.nih.gov/sites/entrez?Db=pubmed&Cmd=Search&Term="Réhault S"%5BAuthor%5D&itool=EntrezSystem2.PEntrez.Pubmed.Pubmed_ResultsPanel.Pubmed_RVAbstractPlusDrugs1), [Monget P](http://www.ncbi.nlm.nih.gov/sites/entrez?Db=pubmed&Cmd=Search&Term="Monget P"%5BAuthor%5D&itool=EntrezSystem2.PEntrez.Pubmed.Pubmed_ResultsPanel.Pubmed_RVAbstractPlusDrugs1), [Mazerbourg S](http://www.ncbi.nlm.nih.gov/sites/entrez?Db=pubmed&Cmd=Search&Term="Mazerbourg S"%5BAuthor%5D&itool=EntrezSystem2.PEntrez.Pubmed.Pubmed_ResultsPanel.Pubmed_RVAbstractPlusDrugs1), [Tremblay R](http://www.ncbi.nlm.nih.gov/sites/entrez?Db=pubmed&Cmd=Search&Term="Tremblay R"%5BAuthor%5D&itool=EntrezSystem2.PEntrez.Pubmed.Pubmed_ResultsPanel.Pubmed_RVAbstractPlusDrugs1), [Gutman N](http://www.ncbi.nlm.nih.gov/sites/entrez?Db=pubmed&Cmd=Search&Term="Gutman N"%5BAuthor%5D&itool=EntrezSystem2.PEntrez.Pubmed.Pubmed_ResultsPanel.Pubmed_RVAbstractPlusDrugs1), [Gauthier F](http://www.ncbi.nlm.nih.gov/sites/entrez?Db=pubmed&Cmd=Search&Term="Gauthier F"%5BAuthor%5D&itool=EntrezSystem2.PEntrez.Pubmed.Pubmed_ResultsPanel.Pubmed_RVAbstractPlusDrugs1), [Moreau T](http://www.ncbi.nlm.nih.gov/sites/entrez?Db=pubmed&Cmd=Search&Term="Moreau T"%5BAuthor%5D&itool=EntrezSystem2.PEntrez.Pubmed.Pubmed_ResultsPanel.Pubmed_RVAbstractPlusDrugs1). (2001). Insulin-like growth factor binding proteins (IGFBPs) as potential physiological substrates for human kallikreins hK2 and hK3. *European Journal of Biochemistry* **268**: 2960-2968

9. [Nam RK, Diamandis EP, Toi A, Trachtenberg J, Magklara A, Scorilas A, Papnastasiou PA, Jewett MA, Narod SA.](http://www.ncbi.nlm.nih.gov/pubmed/10694554?ordinalpos=117&itool=EntrezSystem2.PEntrez.Pubmed.Pubmed_ResultsPanel.Pubmed_RVDocSum) (2000). Serum human glandular kallikrein-2 protease levels predict the presence of prostate cancer among men with elevated prostate-specific antigen. *Journal of Clinical Oncology* **18**: 1036-42.

10. [Kawakami M, Okaneya T, Furihata K, Nishizawa O, Katsuyama T.](http://www.ncbi.nlm.nih.gov/pubmed/9331068?ordinalpos=156&itool=EntrezSystem2.PEntrez.Pubmed.Pubmed_ResultsPanel.Pubmed_RVDocSum)(1997). Detection of prostate cancer cells circulating in peripheral blood by reverse transcription-PCR for hKLK2. *Cancer Research* **57**:4167-4170.

**Gene 3: KLK3**

**Official symbol:** KLK3

**Official Full Name:** kallikrein-related peptidase 3

**Also known as:** APS; PSA; hK3; KLK2A1

**Function:** Serine protease involved in the liquefaction of seminal coagulum, presumably by hydrolysis of the high molecular mass seminal vesicle protein.

**Cancer Correlation:** Serum level of this protein, called PSA in the clinical setting, is useful in the diagnosis and monitoring of prostatic carcinoma.

**References:**

11. [Eeles RA, Kote-Jarai Z, Giles GG, Olama AA, Guy M, Jugurnauth SK, Mulholland S, Leongamornlert DA, Edwards SM, Morrison J, Field HI, Southey MC, Severi G, Donovan JL, Hamdy FC, Dearnaley DP, Muir KR, Smith C, Bagnato M, Ardern-Jones AT, Hall AL, O'Brien LT, Gehr-Swain BN, Wilkinson RA, Cox A, Lewis S, Brown PM, Jhavar SG, Tymrakiewicz M, Lophatananon A, Bryant SL; UK Genetic Prostate Cancer Study Collaborators; British Association of Urological Surgeons' Section of Oncology; UK ProtecT Study Collaborators, Horwich A, Huddart RA, Khoo VS, Parker CC, Woodhouse CJ, Thompson A, Christmas T, Ogden C, Fisher C, Jamieson C, Cooper CS, English DR, Hopper JL, Neal DE, Easton DF.](http://www.ncbi.nlm.nih.gov/pubmed/18264097?ordinalpos=2&itool=EntrezSystem2.PEntrez.Pubmed.Pubmed_ResultsPanel.Pubmed_RVDocSum) (2008). Multiple newly identified loci associated with prostate cancer susceptibility. *Nature genetics*. **40**: 316-321.

12. [Jia L, Coetzee GA.](http://www.ncbi.nlm.nih.gov/pubmed/16140973?ordinalpos=16&itool=EntrezSystem2.PEntrez.Pubmed.Pubmed_ResultsPanel.Pubmed_RVDocSum) (2005). Androgen receptor-dependent PSA expression in androgen-independent prostate cancer cells does not involve androgen receptor occupancy of the PSA locus. *Cancer Research* **65**: 8003-8008.

13. [Romanov VI, Whyard T, Adler HL, Waltzer WC, Zucker S.](http://www.ncbi.nlm.nih.gov/pubmed/15026347?ordinalpos=25&itool=EntrezSystem2.PEntrez.Pubmed.Pubmed_ResultsPanel.Pubmed_RVDocSum) (2004). Prostate cancer cell adhesion to bone marrow endothelium: the role of prostate-specific antigen. *Cancer Research* **64**: 2083-2089.

14. [Gurova KV, Roklin OW, Krivokrysenko VI, Chumakov PM, Cohen MB, Feinstein E, Gudkov AV.](http://www.ncbi.nlm.nih.gov/pubmed/11791186?ordinalpos=13&itool=EntrezSystem2.PEntrez.Pubmed.Pubmed_ResultsPanel.Pubmed_RVDocSum) (2002). Expression of prostate specific antigen (PSA) is negatively regulated by p53. *Oncogene* **21**: 153-157.

15. [Dhanasekaran SM, Barrette TR, Ghosh D, Shah R, Varambally S, Kurachi K, Pienta KJ, Rubin MA, Chinnaiyan AM.](http://www.ncbi.nlm.nih.gov/pubmed/11518967?ordinalpos=1&itool=EntrezSystem2.PEntrez.Pubmed.Pubmed_ResultsPanel.Pubmed_RVDocSum) (2001). Delineation of prognostic biomarkers in prostate cancer. *Nature* **412**: 822-826.

16. [Xue W, Irvine RA, Yu MC, Ross RK, Coetzee GA, Ingles SA.](http://www.ncbi.nlm.nih.gov/pubmed/10706090?ordinalpos=55&itool=EntrezSystem2.PEntrez.Pubmed.Pubmed_ResultsPanel.Pubmed_RVDocSum) (2000). Susceptibility to prostate cancer: interaction between genotypes at the androgen receptor and prostate-specific antigen loci. *Cancer Research* **60**: 839-841.

**Gene 4**: **HAN11**

**Official symbol:** WDR68

**Official Full Name:** WD repeat domain 68

**Also known as:** AN11; HAN11

**Function:** human homologue of anthocyanin (an)11, one of the genes that controls flower pigmentation.

**Cancer Correlation:** already no reported

**References:**

**Gene 5**: **LTF**

**Official symbol: LTF**

**Official Full Name:** lactotransferrin

**Also known as:** HLF2; GIG12

**Function:** non-heme iron binding glycoprotein that modulate iron metabolism, hemopoiesis, and immunologic reactions.

**Cancer Correlation:**  apoptosis and tumor growth

**References:**

19. [Vecchi M, Confalonieri S, Nuciforo P, Viganò MA, Capra M, Bianchi M, Nicosia D, Bianchi F, Galimberti V, Viale G, Palermo G, Riccardi A, Campanini R, Daidone MG, Pierotti MA, Pece S, Di Fiore PP.](http://www.ncbi.nlm.nih.gov/pubmed/17952122?ordinalpos=1&itool=EntrezSystem2.PEntrez.Pubmed.Pubmed_ResultsPanel.Pubmed_RVDocSum) (2007). Breast cancer metastases are molecularly distinct from their primary tumors. *Oncogene* **22** Epub ahead of print

20. [Shaheduzzaman S, Vishwanath A, Furusato B, Cullen J, Chen Y, Bañez L, Nau M, Ravindranath L, Kim KH, Mohammed A, Chen Y, Ehrich M, Srikantan V, Sesterhenn IA, McLeod DG, Vahey M, Petrovics G, Dobi A, Srivastava S.](http://www.ncbi.nlm.nih.gov/pubmed/17568188?ordinalpos=2&itool=EntrezSystem2.PEntrez.Pubmed.Pubmed_ResultsPanel.Pubmed_RVDocSum) (2007). Silencing of Lactotransferrin Expression by Methylation in Prostate Cancer Progression. *Cancer Biology & Therapy* **20** Epub ahead of print

21. [Klein G, Imreh S, Zabarovsky ER.](http://www.ncbi.nlm.nih.gov/pubmed/17433906?ordinalpos=4&itool=EntrezSystem2.PEntrez.Pubmed.Pubmed_ResultsPanel.Pubmed_RVDocSum) (2007). Why do we not all die of cancer at an early age? *Advances in Cancer rresearch* **98**:1-16.

22. [Iijima H, Tomizawa Y, Iwasaki Y, Sato K, Sunaga N, Dobashi K, Saito R, Nakajima T, Minna JD, Mori M.](http://www.ncbi.nlm.nih.gov/pubmed/16152584?ordinalpos=9&itool=EntrezSystem2.PEntrez.Pubmed.Pubmed_ResultsPanel.Pubmed_RVDocSum) (2006). Genetic and epigenetic inactivation of LTF gene at 3p21.3 in lung cancers. [*International journal of cancer*](http://www.ncbi.nlm.nih.gov/sites/entrez?Db=journals&Cmd=ShowDetailView&TermToSearch=4284)118: 797-801.

23. Ward PP, Paz E, Conneely OM. (2005). Multifunctional roles of lactoferrin: A critical overview. *Cellular and Molecular Life Sciences* **62**:2540‑2548.

24. Tsuda H, Sekine K, Fujita K, Ligo M. (2002). Cancer prevention by bovine lactoferrin and underly­ing mechanisms—A review of experimental and clinical studies. [*Biochemistry and Cell Biology*](http://www.ncbi.nlm.nih.gov/sites/entrez?Db=journals&Cmd=ShowDetailView&TermToSearch=1567) **80**:131‑136.

25. [Yoo YC](http://www.ncbi.nlm.nih.gov/sites/entrez?Db=pubmed&Cmd=Search&Term="Yoo YC"%5BAuthor%5D&itool=EntrezSystem2.PEntrez.Pubmed.Pubmed_ResultsPanel.Pubmed_RVAbstractPlusDrugs1), [Watanabe R](http://www.ncbi.nlm.nih.gov/sites/entrez?Db=pubmed&Cmd=Search&Term="Watanabe R"%5BAuthor%5D&itool=EntrezSystem2.PEntrez.Pubmed.Pubmed_ResultsPanel.Pubmed_RVAbstractPlusDrugs1), [Koike Y](http://www.ncbi.nlm.nih.gov/sites/entrez?Db=pubmed&Cmd=Search&Term="Koike Y"%5BAuthor%5D&itool=EntrezSystem2.PEntrez.Pubmed.Pubmed_ResultsPanel.Pubmed_RVAbstractPlusDrugs1), [Mitobe M](http://www.ncbi.nlm.nih.gov/sites/entrez?Db=pubmed&Cmd=Search&Term="Mitobe M"%5BAuthor%5D&itool=EntrezSystem2.PEntrez.Pubmed.Pubmed_ResultsPanel.Pubmed_RVAbstractPlusDrugs1), [Shimazaki K](http://www.ncbi.nlm.nih.gov/sites/entrez?Db=pubmed&Cmd=Search&Term="Shimazaki K"%5BAuthor%5D&itool=EntrezSystem2.PEntrez.Pubmed.Pubmed_ResultsPanel.Pubmed_RVAbstractPlusDrugs1), [Watanabe S](http://www.ncbi.nlm.nih.gov/sites/entrez?Db=pubmed&Cmd=Search&Term="Watanabe S"%5BAuthor%5D&itool=EntrezSystem2.PEntrez.Pubmed.Pubmed_ResultsPanel.Pubmed_RVAbstractPlusDrugs1), [Azuma I](http://www.ncbi.nlm.nih.gov/sites/entrez?Db=pubmed&Cmd=Search&Term="Azuma I"%5BAuthor%5D&itool=EntrezSystem2.PEntrez.Pubmed.Pubmed_ResultsPanel.Pubmed_RVAbstractPlusDrugs1). (1997). Apoptosis in human leukemic cells induced by lac­toferricin, a bovine milk protein‑derived peptide: Involvement of reactive oxygen species. *Biochemical and biophysical research communications* **237**:624‑628.

**Gene 6**: **CSRP1**

**Official symbol: CSRP1**

**Official Full Name:** cysteine and glycine-rich protein 1

**Also known as:** CRP; CRP1; CSRP; CYRP; D1S181E; DKFZp686M148

**Function:** member of the CSRP family of genes encoding a group of LIM domain proteins, which may be involved in regulatory processes important for development and cellular differentiation.

**Cancer Correlation:** Potencial biomarker of malignancy.

**References:**

26. [Miyasaka KY, Kida YS, Sato T, Minami M, Ogura T.](http://www.ncbi.nlm.nih.gov/pubmed/17592114?ordinalpos=1&itool=EntrezSystem2.PEntrez.Pubmed.Pubmed_ResultsPanel.Pubmed_RVDocSum) (2007). Csrp1 regulates dynamic cell movements of the mesendoderm and cardiac mesoderm through interactions with Dishevelled and Diversin. *Proceedings of the National Academy of Sciences of the United States of América* **104**: 11274-1179.

27. [Hirasawa Y, Arai M, Imazeki F, Tada M, Mikata R, Fukai K, Miyazaki M, Ochiai T, Saisho H, Yokosuka O.](http://www.ncbi.nlm.nih.gov/pubmed/17341888?ordinalpos=2&itool=EntrezSystem2.PEntrez.Pubmed.Pubmed_ResultsPanel.Pubmed_RVDocSum) (2006). Methylation status of genes upregulated by demethylating agent 5-aza-2'-deoxycytidine in hepatocellular carcinoma. *Oncology* **71**:77-85.

28. [Wang X, Lee G, Liebhaber SA, Cooke NE.](http://www.ncbi.nlm.nih.gov/pubmed/1374386?ordinalpos=11&itool=EntrezSystem2.PEntrez.Pubmed.Pubmed_ResultsPanel.Pubmed_RVDocSum) (1992). Human cysteine-rich protein. A member of the LIM/double-finger family displaying coordinate serum induction with c-myc. *Journal of biological chemistry* **267**:9176-84.

**Gene 7**: **TGM4**

**Official symbol:** TGM4

**Official Full Name:** transglutaminase 4 (prostate)

**Also known as:** TGP; hTGP

**Function:** Catalyze the posttranslational modification of proteins by the formation of epsilon-(gamma-glutamyl) lysine isopeptide

**Cancer Correlation:** Candidate marker of human prostate cancers

**References:**

29. [Thielen JL, Volzing KG, Collier LS, Green LE, Largaespada DA, Marker PC.](http://www.ncbi.nlm.nih.gov/pubmed/17244021?ordinalpos=1&itool=EntrezSystem2.PEntrez.Pubmed.Pubmed_ResultsPanel.Pubmed_RVDocSum) (2007). Markers of prostate region-specific epithelial identity define anatomical locations in the mouse prostate that are molecularly similar to human prostate cancers. *Differentiation* **75**: 49-61.

30. [Kholodnyuk ID, Szeles A, Yang Y, Klein G, Imreh S.](http://www.ncbi.nlm.nih.gov/pubmed/11156420?ordinalpos=2&itool=EntrezSystem2.PEntrez.Pubmed.Pubmed_ResultsPanel.Pubmed_RVDocSum) (2000). Inactivation of the human fragile histidine triad gene at 3p14.2 in monochromosomal human/mouse microcell hybrid-derived severe combined immunodeficient mouse tumors. *Cancer Research* **60**: 7119-7125.

31. [Dubbink HJ, Cleutjens KB, van der Korput HA, Trapman J, Romijn JC.](http://www.ncbi.nlm.nih.gov/pubmed/10580145?ordinalpos=3&itool=EntrezSystem2.PEntrez.Pubmed.Pubmed_ResultsPanel.Pubmed_RVDocSum) (1999). An Sp1 binding site is essential for basal activity of the human prostate-specific transglutaminase gene (TGM4) promoter. *Gene* **240**: 261-267.

32. [Dubbink HJ, de Waal L, van Haperen R, Verkaik NS, Trapman J, Romijn JC.](http://www.ncbi.nlm.nih.gov/pubmed/9721214?ordinalpos=4&itool=EntrezSystem2.PEntrez.Pubmed.Pubmed_ResultsPanel.Pubmed_RVDocSum) (1998). The human prostate-specific transglutaminase gene (TGM4): genomic organization, tissue-specific expression, and promoter characterization. *Genomics* **51**: 434-444.

**Gene 8: ACTG2**

**Official symbol:** ACTG2

**Official Full Name:** actin, gamma 2, smooth muscle, enteric

**Also known as:** CT; ACTE; ACTA3; ACTL3; ACTSG

**References:**

33. [van Wijngaarden P, Brereton HM, Coster DJ, Williams KA.](http://www.ncbi.nlm.nih.gov/pubmed/17893650?ordinalpos=2&itool=EntrezSystem2.PEntrez.Pubmed.Pubmed_ResultsPanel.Pubmed_RVDocSum) (2007). Stability of housekeeping gene expression in the rat retina during exposure to cyclic hyperoxia. *Molecular Vision* **13**: 1508-1515.

34. [Watson MB, Lind MJ, Smith L, Drew PJ, Cawkwell L.](http://www.ncbi.nlm.nih.gov/pubmed/17562441?ordinalpos=3&itool=EntrezSystem2.PEntrez.Pubmed.Pubmed_ResultsPanel.Pubmed_RVDocSum) (2007). Expression microarray analysis reveals genes associated with in vitro resistance to cisplatin in a cell line model. [*Acta oncológica*](http://www.ncbi.nlm.nih.gov/sites/entrez?Db=journals&Cmd=ShowDetailView&TermToSearch=144) **46**: 651-658.

35. [Wimmers K, Lin CL, Tholen E, Jennen DG, Schellander K, Ponsuksili S.](http://www.ncbi.nlm.nih.gov/pubmed/15771727?ordinalpos=5&itool=EntrezSystem2.PEntrez.Pubmed.Pubmed_ResultsPanel.Pubmed_RVDocSum) (2005). Polymorphisms in candidate genes as markers for sperm quality and boar fertility. *Animal genetics* **36**: 152-155.

36. [Untergasser G, Gander R, Lilg C, Lepperdinger G, Plas E, Berger P.](http://www.ncbi.nlm.nih.gov/pubmed/15610763?ordinalpos=6&itool=EntrezSystem2.PEntrez.Pubmed.Pubmed_ResultsPanel.Pubmed_RVDocSum) (2005). Profiling molecular targets of TGF-beta1 in prostate fibroblast-to-myofibroblast transdifferentiation. *Mechanisms of ageing and development* **126**:59-69.

**Gene 9: MYL6**

**Official symbol: MYL6**

**Official Full Name:** myosin, light chain 6, alkali, smooth muscle and non-muscle

**Also known as:** ESMLC; LC17A; LC17B; MLC1SM; MLC3NM; MLC3SM; LC17-GI; LC17-NM

**References:**

37. [Eichenmüller M, Bauer R, Von Schweinitz D, Hahn H, Kappler R.](http://www.ncbi.nlm.nih.gov/pubmed/17611698?ordinalpos=1&itool=EntrezSystem2.PEntrez.Pubmed.Pubmed_ResultsPanel.Pubmed_RVDocSum) (2007). Hedgehog-independent overexpression of transforming growth factor-beta1 in rhabdomyosarcoma of Patched1 mutant mice. *International journal of oncology* **31**:405-412.

38. [Li C, Kato M, Shiue L, Shively JE, Ares M Jr, Lin RJ.](http://www.ncbi.nlm.nih.gov/pubmed/16488998?ordinalpos=3&itool=EntrezSystem2.PEntrez.Pubmed.Pubmed_ResultsPanel.Pubmed_RVDocSum) (2006). Cell type and culture condition-dependent alternative splicing in human breast cancer cells revealed by splicing-sensitive microarrays. *Cancer Research* **66**:1990-1999.

39. [Luster MI, Simeonova PP, Gallucci RM, Bruccoleri A, Blazka ME, Yucesoy B, Matheson JM.](http://www.ncbi.nlm.nih.gov/pubmed/11083111?ordinalpos=5&itool=EntrezSystem2.PEntrez.Pubmed.Pubmed_ResultsPanel.Pubmed_RVDocSum) (2000). The role of tumor necrosis factor alpha in chemical-induced hepatotoxicity. *Annals of the New York Academy of Sciences* **919**:214-220.

40. [Konfortov BA, Jørgensen CB, Miller JR, Tucker EM.](http://www.ncbi.nlm.nih.gov/pubmed/9745669?ordinalpos=7&itool=EntrezSystem2.PEntrez.Pubmed.Pubmed_ResultsPanel.Pubmed_RVDocSum) (1998). Characterisation of a bovine/murine hybrid cell panel informative for all bovine autosomes. *Animal genetics* **29**: 302-306.

**Gene 10: RDH11**

**Official symbol: RDH11**

**Official Full Name:** retinol dehydrogenase 11 (all-trans/9-cis/11-cis)

**Also known as:** MDT1; PSDR1; RALR1; SCALD; ARSDR1; CGI-82; HCBP12; FLJ32633

**References:**

41. [Edwards S, Campbell C, Flohr P, Shipley J, Giddings I, Te-Poele R, Dodson A, Foster C, Clark J, Jhavar S, Kovacs G, Cooper CS.](http://www.ncbi.nlm.nih.gov/pubmed/15583692?ordinalpos=1&itool=EntrezSystem2.PEntrez.Pubmed.Pubmed_ResultsPanel.Pubmed_RVDocSum) (2005). Expression analysis onto microarrays of randomly selected cDNA clones highlights HOXB13 as a marker of human prostate cancer. [*British journal of cancer*](http://www.ncbi.nlm.nih.gov/sites/entrez?Db=journals&Cmd=ShowDetailView&TermToSearch=1765) **92**: 376-381.

42. [Belyaeva OV, Stetsenko AV, Nelson P, Kedishvili NY.](http://www.ncbi.nlm.nih.gov/pubmed/14674758?ordinalpos=2&itool=EntrezSystem2.PEntrez.Pubmed.Pubmed_ResultsPanel.Pubmed_RVDocSum) (2003). Properties of short-chain dehydrogenase/reductase RalR1: characterization of purified enzyme, its orientation in the microsomal membrane, and distribution in human tissues and cell lines. *Biochemistry* **42**: 14838-14845.

43. [Moore S, Pritchard C, Lin B, Ferguson C, Nelson PS.](http://www.ncbi.nlm.nih.gov/pubmed/12137953?ordinalpos=3&itool=EntrezSystem2.PEntrez.Pubmed.Pubmed_ResultsPanel.Pubmed_RVDocSum) (2002). Isolation and characterization of the murine prostate short-chain dehydrogenase/reductase 1 (Psdr1) gene, a new member of the short-chain steroid dehydrogenase/reductase family. *Gene* **293**: 149-160.

44. [Kedishvili NY, Chumakova OV, Chetyrkin SV, Belyaeva OV, Lapshina EA, Lin DW, Matsumura M, Nelson PS.](http://www.ncbi.nlm.nih.gov/pubmed/12036956?ordinalpos=4&itool=EntrezSystem2.PEntrez.Pubmed.Pubmed_ResultsPanel.Pubmed_RVDocSum) (2002). Evidence that the human gene for prostate short-chain dehydrogenase/reductase (PSDR1) encodes a novel retinal reductase (RalR1). *Journal of Biological Chemistry* **277**: 28909-28915.

45. [Lin B, White JT, Ferguson C, Wang S, Vessella R, Bumgarner R, True LD, Hood L, Nelson PS.](http://www.ncbi.nlm.nih.gov/pubmed/11245473?ordinalpos=5&itool=EntrezSystem2.PEntrez.Pubmed.Pubmed_ResultsPanel.Pubmed_RVDocSum) (2001). Prostate short-chain dehydrogenase reductase 1 (PSDR1): a new member of the short-chain steroid dehydrogenase/reductase family highly expressed in normal and neoplastic prostate epithelium. *Cancer Research* **61**: 1611-1618.

**Gene 11: AZGP1**

**Official symbol: AZGP1**

**Official Full Name:** alpha-2-glycoprotein 1, zinc-binding

**Also known as:** ZAG; ZA2G

**References:**

46. [Lapointe J, Malhotra S, Higgins JP, Bair E, Thompson M, Salari K, Giacomini CP, Ferrari M, Montgomery K, Tibshirani R, van de Rijn M, Brooks JD, Pollack JR.](http://www.ncbi.nlm.nih.gov/pubmed/18223322?ordinalpos=1&itool=EntrezSystem2.PEntrez.Pubmed.Pubmed_ResultsPanel.Pubmed_RVDocSum) (2008). hCAP-D3 expression marks a prostate cancer subtype with favorable clinical behavior and androgen signaling signature. *American Journal of Surgical Pathology* **32**: 205-209.

47. [Falvella FS, Spinola M, Pignatiello C, Noci S, Conti B, Pastorino U, Carbone A, Dragani TA.](http://www.ncbi.nlm.nih.gov/pubmed/17724461?ordinalpos=3&itool=EntrezSystem2.PEntrez.Pubmed.Pubmed_ResultsPanel.Pubmed_RVDocSum) (2007). AZGP1 mRNA levels in normal human lung tissue correlate with lung cancer disease status. *Oncogene* Epub ahead of print

48. [Bondar OP, Barnidge DR, Klee EW, Davis BJ, Klee GG.](http://www.ncbi.nlm.nih.gov/pubmed/17317883?ordinalpos=3&itool=EntrezSystem2.PEntrez.Pubmed.Pubmed_ResultsPanel.Pubmed_RVDocSum) (2007). LC-MS/MS quantification of Zn-alpha2 glycoprotein: a potential serum biomarker for prostate cancer. *Clinical Chemistry* **53**: 673-678.

49. [Henshall SM, Horvath LG, Quinn DI, Eggleton SA, Grygiel JJ, Stricker PD, Biankin AV, Kench JG, Sutherland RL.](http://www.ncbi.nlm.nih.gov/pubmed/17018789?ordinalpos=4&itool=EntrezSystem2.PEntrez.Pubmed.Pubmed_ResultsPanel.Pubmed_RVDocSum) (2006). Zinc-alpha2-glycoprotein expression as a predictor of metastatic prostate cancer following radical prostatectomy*.* [*Journal of the National Cancer Institute*](http://www.ncbi.nlm.nih.gov/sites/entrez?Db=journals&Cmd=ShowDetailView&TermToSearch=4982) **98**:1420-1424.

50. [Descazeaud A, de la Taille A, Allory Y, Faucon H, Salomon L, Bismar T, Kim R, Hofer MD, Chopin D, Abbou CC, Rubin MA.](http://www.ncbi.nlm.nih.gov/pubmed/16598739?ordinalpos=5&itool=EntrezSystem2.PEntrez.Pubmed.Pubmed_ResultsPanel.Pubmed_RVDocSum) (2006). Characterization of ZAG protein expression in prostate cancer using a semi-automated microscope system. *Prostate* **66**: 1037-1043.

51. [Lapointe J, Li C, Higgins JP, van de Rijn M, Bair E, Montgomery K, Ferrari M, Egevad L, Rayford W, Bergerheim U, Ekman P, DeMarzo AM, Tibshirani R, Botstein D, Brown PO, Brooks JD, Pollack JR.](http://www.ncbi.nlm.nih.gov/pubmed/14711987?ordinalpos=6&itool=EntrezSystem2.PEntrez.Pubmed.Pubmed_ResultsPanel.Pubmed_RVDocSum) (2004). Gene expression profiling identifies clinically relevant subtypes of prostate cancer. *Proceedings of the National Academy of Sciences of the United States of América* **101**: 811-816.

**Gene 12: DJ462O23.2**

**Official symbol:** NPAL3

**Official Full Name:** NIPA-like domain containing 3

**Also known as:** DJ462O23.2; RP3-462O23.3; DKFZp686E22155

**References:**

**Gene 13: PRO1073**

**Official symbol:**

**Official Full Name:** PRO1073 protein

**Also known as:**

**References:**

**Gene 14: FXYD3**

**Official symbol: FXYD3**

**Official Full Name:** FXYD domain containing ion transport regulator 3

**Also known as:** MAT8; PLML; MAT-8; MGC111076

**References:**

52. [Arimochi J, Ohashi-Kobayashi A, Maeda M.](http://www.ncbi.nlm.nih.gov/pubmed/17409496?ordinalpos=1&itool=EntrezSystem2.PEntrez.Pubmed.Pubmed_ResultsPanel.Pubmed_RVDocSum) (2007). Interaction of Mat-8 (FXYD-3) with Na+/K+-ATPase in colorectal cancer cells. *Biological & Pharmaceutical Bulletin* **30**: 648-654.

53. [Kayed H, Kleeff J, Kolb A, Ketterer K, Keleg S, Felix K, Giese T, Penzel R, Zentgraf H, Büchler MW, Korc M, Friess H.](http://www.ncbi.nlm.nih.gov/pubmed/16003754?ordinalpos=2&itool=EntrezSystem2.PEntrez.Pubmed.Pubmed_ResultsPanel.Pubmed_RVDocSum) (2006). FXYD3 is overexpressed in pancreatic ductal adenocarcinoma and influences pancreatic cancer cell growth*.* [*International journal of cancer*](http://www.ncbi.nlm.nih.gov/sites/entrez?Db=journals&Cmd=ShowDetailView&TermToSearch=4284) **118**:43-54.

54. [Grzmil M, Voigt S, Thelen P, Hemmerlein B, Helmke K, Burfeind P.](http://www.ncbi.nlm.nih.gov/pubmed/14654946?ordinalpos=3&itool=EntrezSystem2.PEntrez.Pubmed.Pubmed_ResultsPanel.Pubmed_RVDocSum) (2004). Up-regulated expression of the MAT-8 gene in prostate cancer and its siRNA-mediated inhibition of expression induces a decrease in proliferation of human prostate carcinoma cells. *InternationalJournal of Oncology* **24**: 97-105.

55. [Morrison BW, Moorman JR, Kowdley GC, Kobayashi YM, Jones LR, Leder P.](http://www.ncbi.nlm.nih.gov/pubmed/7836447?ordinalpos=4&itool=EntrezSystem2.PEntrez.Pubmed.Pubmed_ResultsPanel.Pubmed_RVDocSum) (1995). Mat-8, a novel phospholemman-like protein expressed in human breast tumors, induces a chloride conductance in Xenopus oocytes. *Journal of Biological Chemistry* **270**: 2176-2182.

**Gene 15: TPM2**

**Official symbol: TPM2**

**Official Full Name:** tropomyosin 2 (beta)

**Also known as:** DA1; TMSB; AMCD1

**References:**

56. [Li DQ, Wang L, Fei F, Hou YF, Luo JM; Wei-Chen, Zeng R, Wu J, Lu JS, Di GH, Ou ZL, Xia QC, Shen ZZ, Shao ZM.](http://www.ncbi.nlm.nih.gov/pubmed/16637015?ordinalpos=1&itool=EntrezSystem2.PEntrez.Pubmed.Pubmed_ResultsPanel.Pubmed_RVDocSum) (2006). Identification of breast cancer metastasis-associated proteins in an isogenic tumor metastasis model using two-dimensional gel electrophoresis and liquid chromatography-ion trap-mass spectrometry*. Proteomics* **6**: 3352-3368.

57. [van't Veer MB, Brooijmans AM, Langerak AW, Verhaaf B, Goudswaard CS, Graveland WJ, van Lom K, Valk PJ.](http://www.ncbi.nlm.nih.gov/pubmed/16434371?ordinalpos=2&itool=EntrezSystem2.PEntrez.Pubmed.Pubmed_ResultsPanel.Pubmed_RVDocSum) (2006). The predictive value of lipoprotein lipase for survival in chronic lymphocytic leukemia. *Haematologica* **91**:56-63.

58. [Varga AE, Stourman NV, Zheng Q, Safina AF, Quan L, Li X, Sossey-Alaoui K, Bakin AV.](http://www.ncbi.nlm.nih.gov/pubmed/15897890?ordinalpos=3&itool=EntrezSystem2.PEntrez.Pubmed.Pubmed_ResultsPanel.Pubmed_RVDocSum) (2005). Silencing of the Tropomyosin-1 gene by DNA methylation alters tumor suppressor function of TGF-beta. *Oncogene* **24**: 5043-5052.

**Gene 16: CRYAB**

**Official symbol:** CRYAB

**Official Full Name:** crystallin, alpha B

**Also known as:** CRYA2; CTPP2; HSPB5

**References:**

59. [Arrigo AP, Simon S, Gibert B, Kretz-Remy C, Nivon M, Czekalla A, Guillet D, Moulin M, Diaz-Latoud C, Vicart P.](http://www.ncbi.nlm.nih.gov/pubmed/17467701?ordinalpos=1&itool=EntrezSystem2.PEntrez.Pubmed.Pubmed_ResultsPanel.Pubmed_RVDocSum) (2007). Hsp27 (HspB1) and alphaB-crystallin (HspB5) as therapeutic targets. *FEBS Letters* 581: 3665-3674.

60. [Seitz S, Korsching E, Weimer J, Jacobsen A, Arnold N, Meindl A, Arnold W, Gustavus D, Klebig C, Petersen I, Scherneck S.](http://www.ncbi.nlm.nih.gov/pubmed/16552773?ordinalpos=2&itool=EntrezSystem2.PEntrez.Pubmed.Pubmed_ResultsPanel.Pubmed_RVDocSum) (2006). Genetic background of different cancer cell lines influences the gene set involved in chromosome 8 mediated breast tumor suppression. *Genes Chromosomes Cancer* **45**: 612-627.

61. [Chin D, Boyle GM, Williams RM, Ferguson K, Pandeya N, Pedley J, Campbell CM, Theile DR, Parsons PG, Coman WB.](http://www.ncbi.nlm.nih.gov/pubmed/15995513?ordinalpos=4&itool=EntrezSystem2.PEntrez.Pubmed.Pubmed_ResultsPanel.Pubmed_RVDocSum) (2005). Alpha B-crystallin, a new independent marker for poor prognosis in head and neck cancer. *Laryngoscope* **115**: 1239-1242.

62. [Stronach EA, Sellar GC, Blenkiron C, Rabiasz GJ, Taylor KJ, Miller EP, Massie CE, Al-Nafussi A, Smyth JF, Porteous DJ, Gabra H.](http://www.ncbi.nlm.nih.gov/pubmed/14695176?ordinalpos=6&itool=EntrezSystem2.PEntrez.Pubmed.Pubmed_ResultsPanel.Pubmed_RVDocSum) (2003). Identification of clinically relevant genes on chromosome 11 in a functional model of ovarian cancer tumor suppression. *Cancer Research* **24**: 8648-8655.

63. [Wittig R, Nessling M, Will RD, Mollenhauer J, Salowsky R, Münstermann E, Schick M, Helmbach H, Gschwendt B, Korn B, Kioschis P, Lichter P, Schadendorf D, Poustka A.](http://www.ncbi.nlm.nih.gov/pubmed/12438269?ordinalpos=7&itool=EntrezSystem2.PEntrez.Pubmed.Pubmed_ResultsPanel.Pubmed_RVDocSum) (2002). Candidate genes for cross-resistance against DNA-damaging drugs. *Cancer Research* **62**:6698-6705.

**Gene 17: ACTA2**

**Official symbol: ACTA2**

**Official Full Name:** actin, alpha 2, smooth muscle, aorta

**Also known as:** ACTSA

**References:**

64. [Casey TM, Eneman J, Crocker A, White J, Tessitore J, Stanley M, Harlow S, Bunn JY, Weaver D, Muss H, Plaut K.](http://www.ncbi.nlm.nih.gov/pubmed/17674196?ordinalpos=1&itool=EntrezSystem2.PEntrez.Pubmed.Pubmed_ResultsPanel.Pubmed_RVDocSum) (2007). Cancer associated fibroblasts stimulated by transforming growth factor beta1 (TGF-beta1) increase invasion rate of tumor cells: a population study. *Breast cancer research and treatment* Epub ahead of print

65. [Tatenhorst L, Senner V, Püttmann S, Paulus W.](http://www.ncbi.nlm.nih.gov/pubmed/15055445?ordinalpos=2&itool=EntrezSystem2.PEntrez.Pubmed.Pubmed_ResultsPanel.Pubmed_RVDocSum) (2004). Regulators of G-protein signaling 3 and 4 (RGS3, RGS4) are associated with glioma cell motility. Journal of Neuropathology and Experimental Neurology **63**: 210-222.

**Gene 18: RPS6**

**Official symbol: RPS6**

**Official Full Name:** ribosomal protein S6

**Also known as:**

**References:**

66. [Ravitz MJ, Chen L, Lynch M, Schmidt EV.](http://www.ncbi.nlm.nih.gov/pubmed/18056446?ordinalpos=1&itool=EntrezSystem2.PEntrez.Pubmed.Pubmed_ResultsPanel.Pubmed_RVDocSum) (2007). c-myc Repression of TSC2 contributes to control of translation initiation and Myc-induced transformation. *Cancer Research* **23**:11209-11217.

67. [Oh HJ, Lee JS, Song DK, Shin DH, Jang BC, Suh SI, Park JW, Suh MH, Baek WK.](http://www.ncbi.nlm.nih.gov/pubmed/17624310?ordinalpos=2&itool=EntrezSystem2.PEntrez.Pubmed.Pubmed_ResultsPanel.Pubmed_RVDocSum) (2007). D-glucosamine inhibits proliferation of human cancer cells through inhibition of p70S6K. *Biochemical and Biophysical Research Communications* **360**: 840-845.

68. [Martin PM, Aeder SE, Chrestensen CA, Sturgill TW, Hussaini IM.](http://www.ncbi.nlm.nih.gov/pubmed/16832347?ordinalpos=3&itool=EntrezSystem2.PEntrez.Pubmed.Pubmed_ResultsPanel.Pubmed_RVDocSum) (2007). Phorbol 12-myristate 13-acetate and serum synergize to promote rapamycin-insensitive cell proliferation via protein kinase C-eta. *Oncogene* **26**: 407-414.

69. [Hayashi M, Fearns C, Eliceiri B, Yang Y, Lee JD.](http://www.ncbi.nlm.nih.gov/pubmed/16140937?ordinalpos=7&itool=EntrezSystem2.PEntrez.Pubmed.Pubmed_ResultsPanel.Pubmed_RVDocSum) (2005). Big mitogen-activated protein kinase 1/extracellular signal-regulated kinase 5 signaling pathway is essential for tumor-associated angiogenesis. *Cancer Research* **65**: 7699-7706.

**Gene 19: DKFZp761L1417**

**Official symbol:** TMEM130

**Official Full Name:** transmembrane protein 130

**Also known as:** FLJ42643; DKFZp761L1417

**References:**

**Gene 20: ACPP**

**Official symbol: ACPP**

**Official Full Name:** acid phosphatase, prostate

**Also known as:** PAP; ACP3; ACP-3

**References:**

70. [Li SS.](http://www.ncbi.nlm.nih.gov/pubmed/8774111?ordinalpos=1&itool=EntrezSystem2.PEntrez.Pubmed.Pubmed_ResultsPanel.Pubmed_RVDocSum) (1996). Human prostatic acid phosphatase and prostate specific antigen: protein structure, gene organization, and expression in neoplastic and benign tissues. *Kaohsiung Journal of Medical Sciences* 12: 441-447.

71. [Sharief FS, Mohler JL, Sharief Y, Li SS.](http://www.ncbi.nlm.nih.gov/pubmed/7524903?ordinalpos=2&itool=EntrezSystem2.PEntrez.Pubmed.Pubmed_ResultsPanel.Pubmed_RVDocSum) (1994). Expression of human prostatic acid phosphatase and prostate specific antigen genes in neoplastic and benign tissues. *Biochemistry and Molecular Biology International* **33**: 567-574.

72. [Li SS, Sharief FS.](http://www.ncbi.nlm.nih.gov/pubmed/8244395?ordinalpos=3&itool=EntrezSystem2.PEntrez.Pubmed.Pubmed_ResultsPanel.Pubmed_RVDocSum) (1993). The prostatic acid phosphatase (ACPP) gene is localized to human chromosome 3q21-q23. *Genomics* **17**: 765-766.

73. [Leskelä S, Virkkunen P, Lukkarinen O, Winqvist R, Vihko P.](http://www.ncbi.nlm.nih.gov/pubmed/1713142?ordinalpos=4&itool=EntrezSystem2.PEntrez.Pubmed.Pubmed_ResultsPanel.Pubmed_RVDocSum) (1991). Lack of association between prostate-specific acid phosphatase RFLP genotypes and prostatic cancer or benign prostatic hyperplasia. *Cytogenetics and Cell Genetics* **57**: 9-10.

74. [Winqvist R, Virkkunen P, Grzeschik KH, Vihko P.](http://www.ncbi.nlm.nih.gov/pubmed/2575485?ordinalpos=5&itool=EntrezSystem2.PEntrez.Pubmed.Pubmed_ResultsPanel.Pubmed_RVDocSum) (1989). Chromosomal localization to 3q21----qter and two TaqI RFLPs of the human prostate-specific acid phosphatase gene (ACPP). *Cytogenetics and Cell Genetics* **52**: 68-71.

**Gene 21: PCP4**

**Official symbol: PCP4**

**Official Full Name:** Purkinje cell protein 4

**Also known as:** PEP-19

**References:**

75. [Wei T, Geiser AG, Qian HR, Su C, Helvering LM, Kulkarini NH, Shou J, N'Cho M, Bryant HU, Onyia JE.](http://www.ncbi.nlm.nih.gov/pubmed/17407572?ordinalpos=1&itool=EntrezSystem2.PEntrez.Pubmed.Pubmed_ResultsPanel.Pubmed_RVDocSum) (2007). DNA microarray data integration by ortholog gene analysis reveals potential molecular mechanisms of estrogen-dependent growth of human uterine fibroids. *BMC Women's Health* **7**: 5.

76. [Kanamori T, Takakura K, Mandai M, Kariya M, Fukuhara K, Kusakari T, Momma C, Shime H, Yagi H, Konishi M, Suzuki A, Matsumura N, Nanbu K, Fujita J, Fujii S.](http://www.ncbi.nlm.nih.gov/pubmed/14561813?ordinalpos=2&itool=EntrezSystem2.PEntrez.Pubmed.Pubmed_ResultsPanel.Pubmed_RVDocSum) (2003). PEP-19 overexpression in human uterine leiomyoma. *Molecular Human Reproduction* **9**: 709-717.

**Gene 22: SYNPO2**

**Official symbol: SYNPO2**

**Official Full Name:** synaptopodin 2

**Also known as:** DKFZp686G051

**References:**

77. [Koppen A, Ait-Aissa R, Hopman S, Koster J, Haneveld F, Versteeg R, Valentijn LJ.](http://www.ncbi.nlm.nih.gov/pubmed/17643814?ordinalpos=1&itool=EntrezSystem2.PEntrez.Pubmed.Pubmed_ResultsPanel.Pubmed_RVDocSum) (2007). Dickkopf-1 is down-regulated by MYCN and inhibits neuroblastoma cell proliferation. *Cancer Letters* **256**: 218-228.

78. [Yu YP, Tseng GC, Luo JH.](http://www.ncbi.nlm.nih.gov/pubmed/16979744?ordinalpos=2&itool=EntrezSystem2.PEntrez.Pubmed.Pubmed_ResultsPanel.Pubmed_RVDocSum) (2006). Inactivation of myopodin expression associated with prostate cancer relapse. *Urology* **68**: 578-582.

79. [Yu YP, Luo JH.](http://www.ncbi.nlm.nih.gov/pubmed/16885336?ordinalpos=3&itool=EntrezSystem2.PEntrez.Pubmed.Pubmed_ResultsPanel.Pubmed_RVDocSum) (2006). Myopodin-mediated suppression of prostate cancer cell migration involves interaction with zyxin. *Cancer Research* **66**: 7414-7419.

80. [Jing L, Liu L, Yu YP, Dhir R, Acquafondada M, Landsittel D, Cieply K, Wells A, Luo JH.](http://www.ncbi.nlm.nih.gov/pubmed/15111326?ordinalpos=4&itool=EntrezSystem2.PEntrez.Pubmed.Pubmed_ResultsPanel.Pubmed_RVDocSum) (2004). Expression of myopodin induces suppression of tumor growth and metastasis. *American Journal of Pathology* **164**: 1799-1806.

81. [Lin F, Yu YP, Woods J, Cieply K, Gooding B, Finkelstein P, Dhir R, Krill D, Becich MJ, Michalopoulos G, Finkelstein S, Luo JH.](http://www.ncbi.nlm.nih.gov/pubmed/11696420?ordinalpos=5&itool=EntrezSystem2.PEntrez.Pubmed.Pubmed_ResultsPanel.Pubmed_RVDocSum) (2001). Myopodin, a synaptopodin homologue, is frequently deleted in invasive prostate cancers. *American Journal of Pathology* 159: 1603-1612.

**Gene 23: SORBS1**

**Official symbol: SORBS1**

**Official Full Name:** sorbin and SH3 domain containing 1

**Also known as:** CAP; FLAF2; R85FL; SH3D5; SORB1; SH3P12; FLJ12406; KIAA1296; DKFZp451C066; DKFZp586P1422

**References:**

82. [Lind GE, Skotheim RI, Fraga MF, Abeler VM, Esteller M, Lothe RA.](http://www.ncbi.nlm.nih.gov/pubmed/17029216?ordinalpos=1&itool=EntrezSystem2.PEntrez.Pubmed.Pubmed_ResultsPanel.Pubmed_RVDocSum) (2006). Novel epigenetically deregulated genes in testicular cancer include homeobox genes and SCGB3A1 (HIN-1). *Journal of Pathology* **210**: 441-449.

83. [Vanaja DK, Ballman KV, Morlan BW, Cheville JC, Neumann RM, Lieber MM, Tindall DJ, Young CY.](http://www.ncbi.nlm.nih.gov/pubmed/16489065?ordinalpos=2&itool=EntrezSystem2.PEntrez.Pubmed.Pubmed_ResultsPanel.Pubmed_RVDocSum) (2006). PDLIM4 repression by hypermethylation as a potential biomarker for prostate cancer. [*Clinical cancer research*](http://www.ncbi.nlm.nih.gov/sites/entrez?Db=journals&Cmd=ShowDetailView&TermToSearch=8794) **12**:1128-1136.

84. [Watahiki A, Waki K, Hayatsu N, Shiraki T, Kondo S, Nakamura M, Sasaki D, Arakawa T, Kawai J, Harbers M, Hayashizaki Y, Carninci P.](http://www.ncbi.nlm.nih.gov/pubmed/15782199?ordinalpos=3&itool=EntrezSystem2.PEntrez.Pubmed.Pubmed_ResultsPanel.Pubmed_RVDocSum) (2004). Libraries enriched for alternatively spliced exons reveal splicing patterns in melanocytes and melanomas. *Nature Methods* **1**: 233-239.

85. [Lin WH, Huang CJ, Liu MW, Chang HM, Chen YJ, Tai TY, Chuang LM.](http://www.ncbi.nlm.nih.gov/pubmed/11374898?ordinalpos=6&itool=EntrezSystem2.PEntrez.Pubmed.Pubmed_ResultsPanel.Pubmed_RVDocSum) (2001). Cloning, mapping, and characterization of the human sorbin and SH3 domain containing 1 (SORBS1) gene: a protein associated with c-Abl during insulin signaling in the hepatoma cell line Hep3B. *Genomics* **74**: 12-20.

**Gene 24: MSMB**

**Official symbol: MSMB**

**Official Full Name:** microseminoprotein, beta-

**Also known as:** MSP; PSP; IGBF; MSPB; PN44; PRPS; PSP57; PSP94; PSP-94

**References:**

11. [Eeles RA, Kote-Jarai Z, Giles GG, Olama AA, Guy M, Jugurnauth SK, Mulholland S, Leongamornlert DA, Edwards SM, Morrison J, Field HI, Southey MC, Severi G, Donovan JL, Hamdy FC, Dearnaley DP, Muir KR, Smith C, Bagnato M, Ardern-Jones AT, Hall AL, O'Brien LT, Gehr-Swain BN, Wilkinson RA, Cox A, Lewis S, Brown PM, Jhavar SG, Tymrakiewicz M, Lophatananon A, Bryant SL; UK Genetic Prostate Cancer Study Collaborators; British Association of Urological Surgeons' Section of Oncology; UK ProtecT Study Collaborators, Horwich A, Huddart RA, Khoo VS, Parker CC, Woodhouse CJ, Thompson A, Christmas T, Ogden C, Fisher C, Jamieson C, Cooper CS, English DR, Hopper JL, Neal DE, Easton DF.](http://www.ncbi.nlm.nih.gov/pubmed/18264097?ordinalpos=1&itool=EntrezSystem2.PEntrez.Pubmed.Pubmed_ResultsPanel.Pubmed_RVDocSum) (2008). Multiple newly identified loci associated with prostate cancer susceptibility. *Nature Genetics* **40**: 316-321.

86. [Thomas G, Jacobs KB, Yeager M, Kraft P, Wacholder S, Orr N, Yu K, Chatterjee N, Welch R, Hutchinson A, Crenshaw A, Cancel-Tassin G, Staats BJ, Wang Z, Gonzalez-Bosquet J, Fang J, Deng X, Berndt SI, Calle EE, Feigelson HS, Thun MJ, Rodriguez C, Albanes D, Virtamo J, Weinstein S, Schumacher FR, Giovannucci E, Willett WC, Cussenot O, Valeri A, Andriole GL, Crawford ED, Tucker M, Gerhard DS, Fraumeni JF Jr, Hoover R, Hayes RB, Hunter DJ, Chanock SJ.](http://www.ncbi.nlm.nih.gov/pubmed/18264096?ordinalpos=2&itool=EntrezSystem2.PEntrez.Pubmed.Pubmed_ResultsPanel.Pubmed_RVDocSum) (2008). Multiple loci identified in a genome-wide association study of prostate cancer. *Nature genetics* **40**: 310-315.

87. [Thielen JL, Volzing KG, Collier LS, Green LE, Largaespada DA, Marker PC.](http://www.ncbi.nlm.nih.gov/pubmed/17244021?ordinalpos=3&itool=EntrezSystem2.PEntrez.Pubmed.Pubmed_ResultsPanel.Pubmed_RVDocSum) (2007). Markers of prostate region-specific epithelial identity define anatomical locations in the mouse prostate that are molecularly similar to human prostate cancers*. Differentiation* **75**: 49-61.

88. [Beke L, Nuytten M, Van Eynde A, Beullens M, Bollen M.](http://www.ncbi.nlm.nih.gov/pubmed/17237810?ordinalpos=4&itool=EntrezSystem2.PEntrez.Pubmed.Pubmed_ResultsPanel.Pubmed_RVDocSum) (2007). The gene encoding the prostatic tumor suppressor PSP94 is a target for repression by the Polycomb group protein EZH2. *Oncogene* **26**: 4590-4595.

89. [Gabril MY, Duan W, Wu G, Moussa M, Izawa JI, Panchal CJ, Sakai H, Xuan JW.](http://www.ncbi.nlm.nih.gov/pubmed/15727931?ordinalpos=5&itool=EntrezSystem2.PEntrez.Pubmed.Pubmed_ResultsPanel.Pubmed_RVDocSum) (2005). A novel knock-in prostate cancer model demonstrates biology similar to that of human prostate cancer and suitable for preclinical studies. *Molecular Therapy* **11**: 348-362.

**Gene 25: ACTC**

**Official symbol: ACTC1**

**Official Full Name:** actin, alpha, cardiac muscle 1

**Also known as:** ACTC; CMD1R

**References:**

**Gene 26: TGFB3**

**Official symbol: TGFB3**

**Official Full Name:** transforming growth factor, beta 3

**Also known as:** ARVD; FLJ16571; TGF-beta3

**References:**

90. [Wyatt L, Wadham C, Crocker LA, Lardelli M, Khew-Goodall Y.](http://www.ncbi.nlm.nih.gov/pubmed/17893246?ordinalpos=5&itool=EntrezSystem2.PEntrez.Pubmed.Pubmed_ResultsPanel.Pubmed_RVDocSum) (2007). The protein tyrosine phosphatase Pez regulates TGFbeta, epithelial-mesenchymal transition, and organ development. *Journal of Cell Biology* **178**: 1223-1235.

91. [Parada D, Arciniegas E, Moreira O, Trujillo E.](http://www.ncbi.nlm.nih.gov/pubmed/15112880?ordinalpos=1&itool=EntrezSystem2.PEntrez.Pubmed.Pubmed_ResultsPanel.Pubmed_RVDocSum) (2004). Transforming growth factor-beta2 and beta3 expression in carcinoma of the prostate. *Archivos Españoles de Urologia* **57**: 93-99.

92. [Hisataki T, Itoh N, Suzuki K, Takahashi A, Masumori N, Tohse N, Ohmori Y, Yamada S, Tsukamoto T.](http://www.ncbi.nlm.nih.gov/pubmed/14716743?ordinalpos=2&itool=EntrezSystem2.PEntrez.Pubmed.Pubmed_ResultsPanel.Pubmed_RVDocSum) (2004). Modulation of phenotype of human prostatic stromal cells by transforming growth factor-betas. *Prostate* **58**: 174-182.

**Gene 27: MALT1**

**Official symbol: MALT1**

**Official Full Name:** mucosa associated lymphoid tissue lymphoma translocation gene 1

**Also known as:** MLT; MLT1; DKFZp434L132

93. [Mahanivong C, Chen HM, Yee SW, Pan ZK, Dong Z, Huang S.](http://www.ncbi.nlm.nih.gov/pubmed/17724468?ordinalpos=2&itool=EntrezSystem2.PEntrez.Pubmed.Pubmed_ResultsPanel.Pubmed_RVDocSum) (2008). Protein kinase Calpha-CARMA3 signaling axis links Ras to NF-kappaB for lysophosphatidic acid-induced urokinase plasminogen activator expression in ovarian cancer cells. *Oncogene* **27**: 1273-1280.

94. [Li C](http://www.ncbi.nlm.nih.gov/sites/entrez?Db=pubmed&Cmd=Search&Term="Li C"%5BAuthor%5D&itool=EntrezSystem2.PEntrez.Pubmed.Pubmed_ResultsPanel.Pubmed_RVAbstractPlusDrugs1), [Hibino M](http://www.ncbi.nlm.nih.gov/sites/entrez?Db=pubmed&Cmd=Search&Term="Hibino M"%5BAuthor%5D&itool=EntrezSystem2.PEntrez.Pubmed.Pubmed_ResultsPanel.Pubmed_RVAbstractPlusDrugs1), [Komatsu H](http://www.ncbi.nlm.nih.gov/sites/entrez?Db=pubmed&Cmd=Search&Term="Komatsu H"%5BAuthor%5D&itool=EntrezSystem2.PEntrez.Pubmed.Pubmed_ResultsPanel.Pubmed_RVAbstractPlusDrugs1), [Sakuma H](http://www.ncbi.nlm.nih.gov/sites/entrez?Db=pubmed&Cmd=Search&Term="Sakuma H"%5BAuthor%5D&itool=EntrezSystem2.PEntrez.Pubmed.Pubmed_ResultsPanel.Pubmed_RVAbstractPlusDrugs1), [Sakakura T](http://www.ncbi.nlm.nih.gov/sites/entrez?Db=pubmed&Cmd=Search&Term="Sakakura T"%5BAuthor%5D&itool=EntrezSystem2.PEntrez.Pubmed.Pubmed_ResultsPanel.Pubmed_RVAbstractPlusDrugs1), [Ueda R](http://www.ncbi.nlm.nih.gov/sites/entrez?Db=pubmed&Cmd=Search&Term="Ueda R"%5BAuthor%5D&itool=EntrezSystem2.PEntrez.Pubmed.Pubmed_ResultsPanel.Pubmed_RVAbstractPlusDrugs1), [Eimoto T](http://www.ncbi.nlm.nih.gov/sites/entrez?Db=pubmed&Cmd=Search&Term="Eimoto T"%5BAuthor%5D&itool=EntrezSystem2.PEntrez.Pubmed.Pubmed_ResultsPanel.Pubmed_RVAbstractPlusDrugs1), [Inagaki H](http://www.ncbi.nlm.nih.gov/sites/entrez?Db=pubmed&Cmd=Search&Term="Inagaki H"%5BAuthor%5D&itool=EntrezSystem2.PEntrez.Pubmed.Pubmed_ResultsPanel.Pubmed_RVAbstractPlusDrugs1). (2008). Primary mucosa-associated lymphoid tissue lymphoma of the prostate: Tumor relapse 7 years after local therapy. *Pathology International* **58**: 191-195.

95. [Izumiyama K, Nakagawa M, Yonezumi M, Kasugai Y, Suzuki R, Suzuki H, Tsuzuki S, Hosokawa Y, Asaka M, Seto M.](http://www.ncbi.nlm.nih.gov/pubmed/14603249?ordinalpos=2&itool=EntrezSystem2.PEntrez.Pubmed.Pubmed_ResultsPanel.Pubmed_RVDocSum)(2003). Stability and subcellular localization of API2-MALT1 chimeric protein involved in t(11;18) (q21;q21) MALT lymphoma. *Oncogene* **22**: 8085-8092.

**Gene 28: FLJ10697**

**Official symbol:** ZNF532

**Official Full Name:** zinc finger protein 532

**Also known as:** FLJ10697

**References:**

**Gene 29: ANXA1**

**Official symbol: ANXA1**

**Official Full Name:** annexin A1

**Also known as:** ANX1; LPC1

**References:**

96. [Silistino-Souza R, Rodrigues-Lisoni FC, Cury PM, Maniglia JV, Raposo LS, Tajara EH, Christian HC, Oliani SM.](http://www.ncbi.nlm.nih.gov/pubmed/17340616?ordinalpos=8&itool=EntrezSystem2.PEntrez.Pubmed.Pubmed_ResultsPanel.Pubmed_RVDocSum) (2007). Annexin 1: differential expression in tumor and mast cells in human larynx cancer. *International Journal of Cancer* **120**: 2582-2589.

97. [Gianni-Barrera R, Gariboldi M, De Cecco L, Manenti G, Dragani TA.](http://www.ncbi.nlm.nih.gov/pubmed/16547502?ordinalpos=15&itool=EntrezSystem2.PEntrez.Pubmed.Pubmed_ResultsPanel.Pubmed_RVDocSum) (2006). Specific gene expression profiles distinguish among functional allelic variants of the mouse Pthlh gene in transfected human cancer cells. *Oncogene* **25**: 4501-4504.

98. [Shen D, He J, Chang HR.](http://www.ncbi.nlm.nih.gov/pubmed/15647832?ordinalpos=20&itool=EntrezSystem2.PEntrez.Pubmed.Pubmed_ResultsPanel.Pubmed_RVDocSum) (2005). In silico identification of breast cancer genes by combined multiple high throughput analyses. *International Journal of Molecular Medicine* **15**: 205-212.

**Gene 30: KIAA0992**

**Official symbol:** PALLD

**Official Full Name:** palladin, cytoskeletal associated protein

**Also known as:** PNCA1; SIH002; CGI-151; FLJ22190; FLJ38193; FLJ39139; KIAA0992

**References:**

99. [Pogue-Geile KL](http://www.ncbi.nlm.nih.gov/sites/entrez?Db=pubmed&Cmd=Search&Term="Pogue-Geile KL"%5BAuthor%5D&itool=EntrezSystem2.PEntrez.Pubmed.Pubmed_ResultsPanel.Pubmed_RVAbstractPlusDrugs1), [Chen R](http://www.ncbi.nlm.nih.gov/sites/entrez?Db=pubmed&Cmd=Search&Term="Chen R"%5BAuthor%5D&itool=EntrezSystem2.PEntrez.Pubmed.Pubmed_ResultsPanel.Pubmed_RVAbstractPlusDrugs1), [Bronner MP](http://www.ncbi.nlm.nih.gov/sites/entrez?Db=pubmed&Cmd=Search&Term="Bronner MP"%5BAuthor%5D&itool=EntrezSystem2.PEntrez.Pubmed.Pubmed_ResultsPanel.Pubmed_RVAbstractPlusDrugs1), [Crnogorac-Jurcevic T](http://www.ncbi.nlm.nih.gov/sites/entrez?Db=pubmed&Cmd=Search&Term="Crnogorac-Jurcevic T"%5BAuthor%5D&itool=EntrezSystem2.PEntrez.Pubmed.Pubmed_ResultsPanel.Pubmed_RVAbstractPlusDrugs1), [Moyes KW](http://www.ncbi.nlm.nih.gov/sites/entrez?Db=pubmed&Cmd=Search&Term="Moyes KW"%5BAuthor%5D&itool=EntrezSystem2.PEntrez.Pubmed.Pubmed_ResultsPanel.Pubmed_RVAbstractPlusDrugs1), [Dowen S](http://www.ncbi.nlm.nih.gov/sites/entrez?Db=pubmed&Cmd=Search&Term="Dowen S"%5BAuthor%5D&itool=EntrezSystem2.PEntrez.Pubmed.Pubmed_ResultsPanel.Pubmed_RVAbstractPlusDrugs1), [Otey CA](http://www.ncbi.nlm.nih.gov/sites/entrez?Db=pubmed&Cmd=Search&Term="Otey CA"%5BAuthor%5D&itool=EntrezSystem2.PEntrez.Pubmed.Pubmed_ResultsPanel.Pubmed_RVAbstractPlusDrugs1), [Crispin DA](http://www.ncbi.nlm.nih.gov/sites/entrez?Db=pubmed&Cmd=Search&Term="Crispin DA"%5BAuthor%5D&itool=EntrezSystem2.PEntrez.Pubmed.Pubmed_ResultsPanel.Pubmed_RVAbstractPlusDrugs1), [George RD](http://www.ncbi.nlm.nih.gov/sites/entrez?Db=pubmed&Cmd=Search&Term="George RD"%5BAuthor%5D&itool=EntrezSystem2.PEntrez.Pubmed.Pubmed_ResultsPanel.Pubmed_RVAbstractPlusDrugs1), [Whitcomb DC](http://www.ncbi.nlm.nih.gov/sites/entrez?Db=pubmed&Cmd=Search&Term="Whitcomb DC"%5BAuthor%5D&itool=EntrezSystem2.PEntrez.Pubmed.Pubmed_ResultsPanel.Pubmed_RVAbstractPlusDrugs1), [Brentnall TA](http://www.ncbi.nlm.nih.gov/sites/entrez?Db=pubmed&Cmd=Search&Term="Brentnall TA"%5BAuthor%5D&itool=EntrezSystem2.PEntrez.Pubmed.Pubmed_ResultsPanel.Pubmed_RVAbstractPlusDrugs1). (2006). Palladin mutation causes familial pancreatic cancer and suggests a new cancer mechanism. *PLoS Medicine* **3**: e516.

.

**Gene 31: MT2A**

**Official symbol:** MT2A

**Official Full Name:** metallothionein 2A

**Also known as:** MT2

**References:**

100. [Yamasaki M, Nomura T, Sato F, Mimata H.](http://www.ncbi.nlm.nih.gov/pubmed/17914565?ordinalpos=3&itool=EntrezSystem2.PEntrez.Pubmed.Pubmed_ResultsPanel.Pubmed_RVDocSum) (2007). Metallothionein is up-regulated under hypoxia and promotes the survival of human prostate cancer cells. *Oncology Reports* **18**: 1145-1153.

101. [Gallagher WM, Bergin OE, Rafferty M, Kelly ZD, Nolan IM, Fox EJ, Culhane AC, McArdle L, Fraga MF, Hughes L, Currid CA, O'Mahony F, Byrne A, Murphy AA, Moss C, McDonnell S, Stallings RL, Plumb JA, Esteller M, Brown R, Dervan PA, Easty DJ.](http://www.ncbi.nlm.nih.gov/pubmed/15958521?ordinalpos=10&itool=EntrezSystem2.PEntrez.Pubmed.Pubmed_ResultsPanel.Pubmed_RVDocSum) (2005). Multiple markers for melanoma progression regulated by DNA methylation: insights from transcriptomic studies. *Carcinogenesis* **26**: 1856-1867.

**Gene 32: ING5**

**Official symbol: ING5**

**Official Full Name:** inhibitor of growth family, member 5

**Also known as:** p28ING5; FLJ23842

102. [Cengiz B, Gunduz M, Nagatsuka H, Beder L, Gunduz E, Tamamura R, Mahmut N, Fukushima K, Ali MA, Naomoto Y, Shimizu K, Nagai N.](http://www.ncbi.nlm.nih.gov/pubmed/16857411?ordinalpos=1&itool=EntrezSystem2.PEntrez.Pubmed.Pubmed_ResultsPanel.Pubmed_RVDocSum) (2007). Fine deletion mapping of chromosome 2q21-37 shows three preferentially deleted regions in oral cancer. *Oral Oncology* **43**: 241-7.

103. [Doyon Y, Cayrou C, Ullah M, Landry AJ, Côté V, Selleck W, Lane WS, Tan S, Yang XJ, Côté J.](http://www.ncbi.nlm.nih.gov/pubmed/16387653?ordinalpos=2&itool=EntrezSystem2.PEntrez.Pubmed.Pubmed_ResultsPanel.Pubmed_RVDocSum) (2006). ING tumor suppressor proteins are critical regulators of chromatin acetylation required for genome expression and perpetuation. *Molecular Cell* **21**: 51-64.

104. [Shiseki M, Nagashima M, Pedeux RM, Kitahama-Shiseki M, Miura K, Okamura S, Onogi H, Higashimoto Y, Appella E, Yokota J, Harris CC.](http://www.ncbi.nlm.nih.gov/pubmed/12750254?ordinalpos=3&itool=EntrezSystem2.PEntrez.Pubmed.Pubmed_ResultsPanel.Pubmed_RVDocSum) (2003). p29ING4 and p28ING5 bind to p53 and p300, and enhance p53 activity. *Cancer Research* **63**: 2373-2378.

**Gene 33: PGM5**

**Official symbol: PGM5**

**Official Full Name:** phosphoglucomutase 5

**Also known as:** PGMRP

**References:**

**Gene 34: SERPINA3**

**Official symbol: SERPINA3**

**Official Full Name:** serpin peptidase inhibitor, clade A (alpha-1 antiproteinase, antitrypsin), member 3

**Also known as:** ACT; AACT; GIG24; GIG25; MGC88254

**References:**

105. [Demeo DL, Campbell EJ, Barker AF, Brantly ML, Eden E, McElvaney NG, Rennard SI, Sandhaus RA, Stocks JM, Stoller JK, Strange C, Turino G, Silverman EK.](http://www.ncbi.nlm.nih.gov/pubmed/17690329?ordinalpos=1&itool=EntrezSystem2.PEntrez.Pubmed.Pubmed_ResultsPanel.Pubmed_RVDocSum) (2008). IL10 polymorphisms are associated with airflow obstruction in severe alpha1-antitrypsin deficiency. *American Journal of Respiratory Cell and Molecular Biology* **38**: 114-120.

106. [Monleón I, Iturralde M, Martínez-Lorenzo MJ, Monteagudo L, Lasierra P, Larrad L, Piñeiro A, Naval J, Alava MA, Anel A.](http://www.ncbi.nlm.nih.gov/pubmed/12133900?ordinalpos=2&itool=EntrezSystem2.PEntrez.Pubmed.Pubmed_ResultsPanel.Pubmed_RVDocSum) (2002). Lack of Fas/CD95 surface expression in highly proliferative leukemic cell lines correlates with loss of CtBP/BARS and redirection of the protein toward giant lysosomal structures. *Cell growth & Differentiation* **13**: 315-24.

**Gene 35: KRT5**

**Official symbol: KRT5**

**Official Full Name:** keratin 5 (epidermolysis bullosa simplex, Dowling-Meara/Kobner/Weber-Cockayne types)

**Also known as:** K5; CK5; DDD; EBS2; KRT5A

**References:**

107. [Shen D, He J, Chang HR.](http://www.ncbi.nlm.nih.gov/pubmed/15647832?ordinalpos=1&itool=EntrezSystem2.PEntrez.Pubmed.Pubmed_ResultsPanel.Pubmed_RVDocSum) (2005). In silico identification of breast cancer genes by combined multiple high throughput analyses. *International Journal of Molecular Medicine* **15**: 205-12.

108. [Molinié V, Fromont G, Sibony M, Vieillefond A, Vassiliu V, Cochand-Priollet B, Hervé JM, Lebret T, Baglin AC.](http://www.ncbi.nlm.nih.gov/pubmed/15205683?ordinalpos=2&itool=EntrezSystem2.PEntrez.Pubmed.Pubmed_ResultsPanel.Pubmed_RVDocSum) (2004). Diagnostic utility of a p63/alpha-methyl-CoA-racemase (p504s) cocktail in atypical foci in the prostate. Modern Pathology **17**: 1180-1190.

109. [van Leenders GJ, Gage WR, Hicks JL, van Balken B, Aalders TW, Schalken JA, De Marzo AM.](http://www.ncbi.nlm.nih.gov/pubmed/12707036?ordinalpos=3&itool=EntrezSystem2.PEntrez.Pubmed.Pubmed_ResultsPanel.Pubmed_RVDocSum) (2003). Intermediate cells in human prostate epithelium are enriched in proliferative inflammatory atrophy. *American Journal of Pathology* **162**: 1529-1537.

110. [Rumpold H, Heinrich E, Untergasser G, Hermann M, Pfister G, Plas E, Berger P.](http://www.ncbi.nlm.nih.gov/pubmed/12242724?ordinalpos=5&itool=EntrezSystem2.PEntrez.Pubmed.Pubmed_ResultsPanel.Pubmed_RVDocSum) (2002). Neuroendocrine differentiation of human prostatic primary epithelial cells in vitro. *Prostate* **53**: 101-108.

111. [van Leenders GJ, Aalders TW, Hulsbergen-van de Kaa CA, Ruiter DJ, Schalken JA.](http://www.ncbi.nlm.nih.gov/pubmed/11745692?ordinalpos=4&itool=EntrezSystem2.PEntrez.Pubmed.Pubmed_ResultsPanel.Pubmed_RVDocSum) (2001). Expression of basal cell keratins in human prostate cancer metastases and cell lines. *Journal of Pathology* **195**: 563-570.

**Gene 36: RPL5**

**Official symbol: RPL5**

**Official Full Name:** ribosomal protein L5

**Also known as:** MSTP030; MGC117339

**References:**

112. [Lü B, Xu J, Zhu Y, Zhang H, Lai M.](http://www.ncbi.nlm.nih.gov/pubmed/17184759?ordinalpos=1&itool=EntrezSystem2.PEntrez.Pubmed.Pubmed_ResultsPanel.Pubmed_RVDocSum) (2007). Systemic analysis of the differential gene expression profile in a colonic adenoma-normal SSH library. *Clinica Chimica Acta* **378**: 42-47.

113. [Frigerio JM, Dagorn JC, Iovanna JL.](http://www.ncbi.nlm.nih.gov/pubmed/7772601?ordinalpos=2&itool=EntrezSystem2.PEntrez.Pubmed.Pubmed_ResultsPanel.Pubmed_RVDocSum) (1995). Cloning, sequencing and expression of the L5, L21, L27a, L28, S5, S9, S10 and S29 human ribosomal protein mRNAs. *Biochimica et Biophysica Acta* **1262**: 64-68.

**Gene 37: IGF1**

**Official symbol: IGF1**

**Official Full Name:** insulin-like growth factor 1 (somatomedin C)

**Also known as:** IGFI

**References:**

114. [Sarma AV, Dunn RL, Lange LA, Ray A, Wang Y, Lange EM, Cooney KA.](http://www.ncbi.nlm.nih.gov/pubmed/18163429?ordinalpos=1&itool=EntrezSystem2.PEntrez.Pubmed.Pubmed_ResultsPanel.Pubmed_RVDocSum) (2008). Genetic polymorphisms in CYP17, CYP3A4, CYP19A1, SRD5A2, IGF-1, and IGFBP-3 and prostate cancer risk in African-American men: The Flint Men's Health Study. *Prostate* **68**: 296-305.

115. [Fan W, Yanase T, Morinaga H, Okabe T, Nomura M, Daitoku H, Fukamizu A, Kato S, Takayanagi R, Nawata H.](http://www.ncbi.nlm.nih.gov/pubmed/17202144?ordinalpos=4&itool=EntrezSystem2.PEntrez.Pubmed.Pubmed_ResultsPanel.Pubmed_RVDocSum) (2007). Insulin-like growth factor 1/insulin signaling activates androgen signaling through direct interactions of Foxo1 with androgen receptor. *Journal of Biological Chemistry* **282**: 7329-7338.

116. [Johansson M, McKay JD, Stattin P, Canzian F, Boillot C, Wiklund F, Adami HO, Bälter K, Grönberg H, Kaaks R.](http://www.ncbi.nlm.nih.gov/pubmed/17096324?ordinalpos=6&itool=EntrezSystem2.PEntrez.Pubmed.Pubmed_ResultsPanel.Pubmed_RVDocSum) (2007). Comprehensive evaluation of genetic variation in the IGF1 gene and risk of prostate cancer. International Journal of Cancer. 120: 539-542.

117. [Fürstenberger G, Senn HJ.](http://www.ncbi.nlm.nih.gov/pubmed/12067807?ordinalpos=12&itool=EntrezSystem2.PEntrez.Pubmed.Pubmed_ResultsPanel.Pubmed_RVDocSum) (2002). Insulin-like growth factors and cancer. *Lancet Oncology* **3**: 298-302.

**Gene 38: ZNF92**

**Official symbol: ZNF92**

**Official Full Name:** zinc finger protein 92 (HTF12)

**Also known as:** HPF12

**References:**

**Gene 39: FOLH1**

**Official symbol: FOLH1**

**Official Full Name:** folate hydrolase (prostate-specific membrane antigen) 1

**Also known as:** PSM; FGCP; FOLH; GCP2; PSMA; mGCP; GCPII; NAALAD1; NAALAdase

**References:**

118. [Williams T, Kole R.](http://www.ncbi.nlm.nih.gov/pubmed/16764542?ordinalpos=1&itool=EntrezSystem2.PEntrez.Pubmed.Pubmed_ResultsPanel.Pubmed_RVDocSum) (2006). Analysis of prostate-specific membrane antigen splice variants in LNCap cells. *Oligonucleotides* **16**: 186-195.

119. [Schmittgen TD, Zakrajsek BA, Hill RE, Liu Q, Reeves JJ, Axford PD, Singer MJ, Reed MW.](http://www.ncbi.nlm.nih.gov/pubmed/12712410?ordinalpos=4&itool=EntrezSystem2.PEntrez.Pubmed.Pubmed_ResultsPanel.Pubmed_RVDocSum) (2003). Expression pattern of mouse homolog of prostate-specific membrane antigen (FOLH1) in the transgenic adenocarcinoma of the mouse prostate model. *Prostate* **55**:308-316.

120. [Burger MJ, Tebay MA, Keith PA, Samaratunga HM, Clements J, Lavin MF, Gardiner RA.](http://www.ncbi.nlm.nih.gov/pubmed/12115574?ordinalpos=7&itool=EntrezSystem2.PEntrez.Pubmed.Pubmed_ResultsPanel.Pubmed_RVDocSum) (2002). Expression analysis of delta-catenin and prostate-specific membrane antigen: their potential as diagnostic markers for prostate cancer. *International Journal of Cancer* **100**: 228-237.

121. [Noss KR, Wolfe SA, Grimes SR.](http://www.ncbi.nlm.nih.gov/pubmed/12039052?ordinalpos=8&itool=EntrezSystem2.PEntrez.Pubmed.Pubmed_ResultsPanel.Pubmed_RVDocSum) (2002). Upregulation of prostate specific membrane antigen/folate hydrolase transcription by an enhancer. *Gene* **285**: 247-56.

122. [Maraj BH, Markham AF.](http://www.ncbi.nlm.nih.gov/pubmed/12496775?ordinalpos=6&itool=EntrezSystem2.PEntrez.Pubmed.Pubmed_ResultsPanel.Pubmed_RVDocSum) (1999). Prostate-specific membrane antigen (FOLH1): recent advances in characterising this putative prostate cancer gene. *Prostate Cancer and Prostatic Diseases* **2**: 180-185.

**Gene 40: CYR61**

**Official symbol: CYR61**

**Official Full Name:** cysteine-rich, angiogenic inducer, 61

**Also known as:** CCN1; GIG1; IGFBP10

**References:**

123. [Parker TL, Eggett DL, Christensen MJ.](http://www.ncbi.nlm.nih.gov/pubmed/17434722?ordinalpos=1&itool=EntrezSystem2.PEntrez.Pubmed.Pubmed_ResultsPanel.Pubmed_RVDocSum) (2007). Estrogen receptor activation and estrogen-regulated gene expression are unaffected by methylseleninic acid in LNCaP prostate cancer cells. *Journal of nutritional biochemistry* **18**: 746-752.

124. [Hammacher A, Thompson EW, Williams ED.](http://www.ncbi.nlm.nih.gov/pubmed/15474988?ordinalpos=4&itool=EntrezSystem2.PEntrez.Pubmed.Pubmed_ResultsPanel.Pubmed_RVDocSum) (2005). Interleukin-6 is a potent inducer of S100P, which is up-regulated in androgen-refractory and metastatic prostate cancer. *International Journal of Biochemistry & Cell Biology* **37**: 442-450.

125. [van Ginkel PR, Gee RL, Shearer RL, Subramanian L, Walker TM, Albert DM, Meisner LF, Varnum BC, Polans AS.](http://www.ncbi.nlm.nih.gov/pubmed/14729616?ordinalpos=5&itool=EntrezSystem2.PEntrez.Pubmed.Pubmed_ResultsPanel.Pubmed_RVDocSum) (2004). Expression of the receptor tyrosine kinase Axl promotes ocular melanoma cell survival. *Cancer Research* **64**: 128-134.

126. [Pilarsky CP, Schmidt U, Eissrich C, Stade J, Froschermaier SE, Haase M, Faller G, Kirchner TW, Wirth MP.](http://www.ncbi.nlm.nih.gov/pubmed/9655260?ordinalpos=7&itool=EntrezSystem2.PEntrez.Pubmed.Pubmed_ResultsPanel.Pubmed_RVDocSum) (1998). Expression of the extracellular matrix signaling molecule Cyr61 is downregulated in prostate cancer. *Prostate* **36**:85-91.

**Gene 41: FHL1**

**Official symbol: FHL1**

**Official Full Name:** four and a half LIM domains 1

**Also known as:** FHL1B; KYO-T; SLIM1; XMPMA; MGC111107; bA535K18.1

**References:**

127. [Fryknäs M, Wickenberg-Bolin U, Göransson H, Gustafsson MG, Foukakis T, Lee JJ, Landegren U, Höög A, Larsson C, Grimelius L, Wallin G, Pettersson U, Isaksson A.](http://www.ncbi.nlm.nih.gov/pubmed/16675914?ordinalpos=1&itool=EntrezSystem2.PEntrez.Pubmed.Pubmed_ResultsPanel.Pubmed_RVDocSum) (2006). Molecular markers for discrimination of benign and malignant follicular thyroid tumors. *Tumour Biology* **27**: 211-220.

128. [Shen Y, Jia Z, Nagele RG, Ichikawa H, Goldberg GS.](http://www.ncbi.nlm.nih.gov/pubmed/16452211?ordinalpos=2&itool=EntrezSystem2.PEntrez.Pubmed.Pubmed_ResultsPanel.Pubmed_RVDocSum) (2006). SRC uses Cas to suppress Fhl1 in order to promote nonanchored growth and migration of tumor cells. *Cancer Research* **66**: 1543-1552.

129. [Johannessen M, Møller S, Hansen T, Moens U, Van Ghelue M.](http://www.ncbi.nlm.nih.gov/pubmed/16389449?ordinalpos=3&itool=EntrezSystem2.PEntrez.Pubmed.Pubmed_ResultsPanel.Pubmed_RVDocSum) (2006). The multifunctional roles of the four-and-a-half-LIM only protein FHL2. *Cellular and Molecular Life Sciences* **63**: 268-284.

130. [Boissel JP, Bros M, Schröck A, Gödtel-Armbrust U, Förstermann U.](http://www.ncbi.nlm.nih.gov/pubmed/15170357?ordinalpos=4&itool=EntrezSystem2.PEntrez.Pubmed.Pubmed_ResultsPanel.Pubmed_RVDocSum) (2004). Cyclic AMP-mediated upregulation of the expression of neuronal NO synthase in human A673 neuroepithelioma cells results in a decrease in the level of bioactive NO production: analysis of the signaling mechanisms that are involved. *Biochemistry* **43**: 7197-7206.

**Gene 42: H19**

**Official symbol:** H19

**Official Full Name:** H19, imprinted maternally expressed transcript

**Also known as:** ASM; BWS; ASM1; MGC4485; PRO2605; D11S813E

**References:**

131. [Berteaux N, Lottin S, Adriaenssens E, Van Coppenolle F, Leroy X, Coll J, Dugimont T, Curgy JJ.](http://www.ncbi.nlm.nih.gov/pubmed/15525575?ordinalpos=3&itool=EntrezSystem2.PEntrez.Pubmed.Pubmed_ResultsPanel.Pubmed_RVDocSum) (2004). Hormonal regulation of H19 gene expression in prostate epithelial cells. *Journal of Endocrinology* **183**: 69-78.

**Gene 43: DMN**

**Official symbol: DMN**

**Official Full Name:** desmuslin

**Also known as:** SYN; KIAA0353

**References:**

**Gene 44: NEFH**

**Official symbol: NEFH**

**Official Full Name:** neurofilament, heavy polypeptide 200kDa

**Also known as:** NFH

**References:**

132. [Sainio M, Strachan T, Blomstedt G, Salonen O, Setälä K, Palotie A, Palo J, Pyykkö I, Peltonen L, Jääskeläinen J.](http://www.ncbi.nlm.nih.gov/pubmed/7617190?ordinalpos=1&itool=EntrezSystem2.PEntrez.Pubmed.Pubmed_ResultsPanel.Pubmed_RVDocSum) (1995). Presymptomatic DNA and MRI diagnosis of neurofibromatosis 2 with mild clinical course in an extended pedigree. *Neurology* **45**: 1314-1322.

133. [Ruttledge MH, Narod SA, Dumanski JP, Parry DM, Eldridge R, Wertelecki W, Parboosingh J, Faucher MC, Lenoir GM, Collins VP, et al.](http://www.ncbi.nlm.nih.gov/pubmed/8414026?ordinalpos=2&itool=EntrezSystem2.PEntrez.Pubmed.Pubmed_ResultsPanel.Pubmed_RVDocSum) (1993). Presymptomatic diagnosis for neurofibromatosis 2 with chromosome 22 markers. *Neurology* **43**: 1753-1760.

134. [Sanson M, Marineau C, Desmaze C, Lutchman M, Ruttledge M, Baron C, Narod S, Delattre O, Lenoir G, Thomas G, et al.](http://www.ncbi.nlm.nih.gov/pubmed/8401504?ordinalpos=3&itool=EntrezSystem2.PEntrez.Pubmed.Pubmed_ResultsPanel.Pubmed_RVDocSum) (1993). Germline deletion in a neurofibromatosis type 2 kindred inactivates the NF2 gene and a candidate meningioma locus. *Human Molecular Genetics* **2**: 1215-1220.

135. [Watson CJ, Gaunt L, Evans G, Patel K, Harris R, Strachan T.](http://www.ncbi.nlm.nih.gov/pubmed/8102569?ordinalpos=4&itool=EntrezSystem2.PEntrez.Pubmed.Pubmed_ResultsPanel.Pubmed_RVDocSum) (1993). A disease-associated germline deletion maps the type 2 neurofibromatosis (NF2) gene between the Ewing sarcoma region and the leukaemia inhibitory factor locus. *Human Molecular Genetics* **2**: 701-704.

**Gene 45: PPP1R12B**

**Official symbol:** PPP1R12B

**Official Full Name:** protein phosphatase 1, regulatory (inhibitor) subunit 12B

**Also known as:** MYPT2; MGC87886; MGC131980

**References:**

**Gene 46: ANTXR2**

**Official symbol: ANTXR2**

**Official Full Name:** anthrax toxin receptor 2

**Also known as:** ISH; JHF; CMG2; CMG-2; FLJ31074; MGC45856; MGC111533

**References:**

136. [Rogers MS, Christensen KA, Birsner AE, Short SM, Wigelsworth DJ, Collier RJ, D'Amato RJ.](http://www.ncbi.nlm.nih.gov/pubmed/17942931?ordinalpos=1&itool=EntrezSystem2.PEntrez.Pubmed.Pubmed_ResultsPanel.Pubmed_RVDocSum) (2007). Mutant anthrax toxin B moiety (protective antigen) inhibits angiogenesis and tumor growth. *Cancer Research* **67**: 9980-9985.

137. [Chen KH, Liu S, Bankston LA, Liddington RC, Leppla SH.](http://www.ncbi.nlm.nih.gov/pubmed/17251181?ordinalpos=3&itool=EntrezSystem2.PEntrez.Pubmed.Pubmed_ResultsPanel.Pubmed_RVDocSum) (2007). Selection of anthrax toxin protective antigen variants that discriminate between the cellular receptors TEM8 and CMG2 and achieve targeting of tumor cells. *Journal of Biological Chemistry* **282**:9834-9845.

138. [Santelli E, Bankston LA, Leppla SH, Liddington RC.](http://www.ncbi.nlm.nih.gov/pubmed/15243628?ordinalpos=6&itool=EntrezSystem2.PEntrez.Pubmed.Pubmed_ResultsPanel.Pubmed_RVDocSum) (2004). Crystal structure of a complex between anthrax toxin and its host cell receptor. *Nature* **430**:905-908.

**Gene 47: MRLC2**

**Official symbol:**

**Official Full Name:** myosin regulatory light chain MRLC2

**Also known as:** MRLC2; MLC-B

**References:**

139. [Umeda D](http://www.ncbi.nlm.nih.gov/sites/entrez?Db=pubmed&Cmd=Search&Term="Umeda D"%5BAuthor%5D&itool=EntrezSystem2.PEntrez.Pubmed.Pubmed_ResultsPanel.Pubmed_RVAbstractPlusDrugs1), [Tachibana H](http://www.ncbi.nlm.nih.gov/sites/entrez?Db=pubmed&Cmd=Search&Term="Tachibana H"%5BAuthor%5D&itool=EntrezSystem2.PEntrez.Pubmed.Pubmed_ResultsPanel.Pubmed_RVAbstractPlusDrugs1), [Yamada K](http://www.ncbi.nlm.nih.gov/sites/entrez?Db=pubmed&Cmd=Search&Term="Yamada K"%5BAuthor%5D&itool=EntrezSystem2.PEntrez.Pubmed.Pubmed_ResultsPanel.Pubmed_RVAbstractPlusDrugs1). (2005). Epigallocatechin-3-O-gallate disrupts stress fibers and the contractile ring by reducing myosin regulatory light chain phosphorylation mediated through the target molecule 67 kDa laminin receptor. *Biochemical and Biophysical Research Communications* **333**: 628-635.

**Gene 48: C20orf103**

**Official symbol:** C20orf103

**Official Full Name:** chromosome 20 open reading frame 103

**Also known as:**

**References:**

**Gene 49: UBA52**

**Official symbol: UBA52**

**Official Full Name:** ubiquitin A-52 residue ribosomal protein fusion product 1

**Also known as:** CEP52; RPL40; HUBCEP52; MGC57125; MGC126879; MGC126881

**References:**

140. [Barnard GF, Mori M, Staniunas RJ, Begum NA, Bao S, Puder M, Cobb J, Redman KL, Steele GD Jr, Chen LB.](http://www.ncbi.nlm.nih.gov/pubmed/8541345?ordinalpos=1&itool=EntrezSystem2.PEntrez.Pubmed.Pubmed_ResultsPanel.Pubmed_RVDocSum) (1995). Ubiquitin fusion proteins are overexpressed in colon cancer but not in gastric cancer. *Biochimica et Biophysica Acta* **1272**: 147-153.

141. [Kanayama H, Tanaka K, Aki M, Kagawa S, Miyaji H, Satoh M, Okada F, Sato S, Shimbara N, Ichihara A.](http://www.ncbi.nlm.nih.gov/pubmed/1660345?ordinalpos=2&itool=EntrezSystem2.PEntrez.Pubmed.Pubmed_ResultsPanel.Pubmed_RVDocSum) (1991). Changes in expressions of proteasome and ubiquitin genes in human renal cancer cells. *Cancer Research* **51**: 6677-6685.

**Gene 50: TRGV9**

**Official symbol: TRGV9**

**Official Full Name:** T cell receptor gamma variable 9

**Also known as:** V2; TCRGV9; MGC47828

**References:**

**Gene 51: SPARC**

**Official symbol: SPARC**

**Official Full Name:** secreted protein, acidic, cysteine-rich (osteonectin)

**Also known as:** ON

**References:**

142. [Chen N, Ye XC, Chu K, Navone NM, Sage EH, Yu-Lee LY, Logothetis CJ, Lin SH.](http://www.ncbi.nlm.nih.gov/pubmed/17638862?ordinalpos=2&itool=EntrezSystem2.PEntrez.Pubmed.Pubmed_ResultsPanel.Pubmed_RVDocSum) (2007). A secreted isoform of ErbB3 promotes osteonectin expression in bone and enhances the invasiveness of prostate cancer cells. *Cancer Research* **67**: 6544-6548.

143. [Hooi CF, Blancher C, Qiu W, Revet IM, Williams LH, Ciavarella ML, Anderson RL, Thompson EW, Connor A, Phillips WA, Campbell IG.](http://www.ncbi.nlm.nih.gov/pubmed/16474848?ordinalpos=4&itool=EntrezSystem2.PEntrez.Pubmed.Pubmed_ResultsPanel.Pubmed_RVDocSum) (2006). ST7-mediated suppression of tumorigenicity of prostate cancer cells is characterized by remodeling of the extracellular matrix. *Oncogene* **25**: 3924-3933.

144. [Koblinski JE, Kaplan-Singer BR, VanOsdol SJ, Wu M, Engbring JA, Wang S, Goldsmith CM, Piper JT, Vostal JG, Harms JF, Welch DR, Kleinman HK.](http://www.ncbi.nlm.nih.gov/pubmed/16103089?ordinalpos=6&itool=EntrezSystem2.PEntrez.Pubmed.Pubmed_ResultsPanel.Pubmed_RVDocSum) (2005). Endogenous osteonectin/SPARC/BM-40 expression inhibits MDA-MB-231 breast cancer cell metastasis. *Cancer Research* **65**: 7370-7377.

145. [De S, Chen J, Narizhneva NV, Heston W, Brainard J, Sage EH, Byzova TV.](http://www.ncbi.nlm.nih.gov/pubmed/12885781?ordinalpos=8&itool=EntrezSystem2.PEntrez.Pubmed.Pubmed_ResultsPanel.Pubmed_RVDocSum) (2003). Molecular pathway for cancer metastasis to bone. *Journal of Biological Chemistry* **278**: 39044-39050.

146. [Thomas R, True LD, Bassuk JA, Lange PH, Vessella RL.](http://www.ncbi.nlm.nih.gov/pubmed/10741745?ordinalpos=10&itool=EntrezSystem2.PEntrez.Pubmed.Pubmed_ResultsPanel.Pubmed_RVDocSum) (2000). Differential expression of osteonectin/SPARC during human prostate cancer progression. *Clinical Cancer Research* **6**: 1140-1149.

**Gene 52: AMACR**

**Official symbol: AMACR**

**Official Full Name:** alpha-methylacyl-CoA racemase

**Also known as:** RACE

**References:**

147. [Herawi M, Epstein JI.](http://www.ncbi.nlm.nih.gov/pubmed/17527076?ordinalpos=1&itool=EntrezSystem2.PEntrez.Pubmed.Pubmed_ResultsPanel.Pubmed_RVDocSum) (2007). Immunohistochemical antibody cocktail staining (p63/HMWCK/AMACR) of ductal adenocarcinoma and Gleason pattern 4 cribriform and noncribriform acinar adenocarcinomas of the prostate. *American Journal of Surgical Pathology* **31**: 889-894.

148. [Yemelyanov A, Czwornog J, Chebotaev D, Karseladze A, Kulevitch E, Yang X, Budunova I.](http://www.ncbi.nlm.nih.gov/pubmed/17016446?ordinalpos=1&itool=EntrezSystem2.PEntrez.Pubmed.Pubmed_ResultsPanel.Pubmed_RVDocSum) (2007). Tumor suppressor activity of glucocorticoid receptor in the prostate. *Oncogene* **26**: 1885-1896.

149. [Petrovics G, Liu A, Shaheduzzaman S, Furusato B, Sun C, Chen Y, Nau M, Ravindranath L, Chen Y, Dobi A, Srikantan V, Sesterhenn IA, McLeod DG, Vahey M, Moul JW, Srivastava S.](http://www.ncbi.nlm.nih.gov/pubmed/15750627?ordinalpos=2&itool=EntrezSystem2.PEntrez.Pubmed.Pubmed_ResultsPanel.Pubmed_RVDocSum) (2005). Frequent overexpression of ETS-related gene-1 (ERG1) in prostate cancer transcriptome. *Oncogene* **24**: 3847-3852.

150. [Luo J, Zha S, Gage WR, Dunn TA, Hicks JL, Bennett CJ, Ewing CM, Platz EA, Ferdinandusse S, Wanders RJ, Trent JM, Isaacs WB, De Marzo AM.](http://www.ncbi.nlm.nih.gov/pubmed/11956072?ordinalpos=6&itool=EntrezSystem2.PEntrez.Pubmed.Pubmed_ResultsPanel.Pubmed_RVDocSum) (2002). Alpha-methylacyl-CoA racemase: a new molecular marker for prostate cancer. *Cancer Research* **62**: 2220-2226.

**Gene 53: DNER**

**Official symbol: DNER**

**Official Full Name:** delta/notch-like EGF repeat containing

**Also known as:** bet; UNQ26

**References:**

151. [Stahl S, Reinders Y, Asan E, Mothes W, Conzelmann E, Sickmann A, Felbor U.](http://www.ncbi.nlm.nih.gov/pubmed/17765022?ordinalpos=1&itool=EntrezSystem2.PEntrez.Pubmed.Pubmed_ResultsPanel.Pubmed_RVDocSum) (2007). Proteomic analysis of cathepsin B- and L-deficient mouse brain lysosomes. *Biochimica et biophysica acta* **1774**: 1237-1246.

152. [Hassan MH, Salama SA, Arafa HM, Hamada FM, Al-Hendy A.](http://www.ncbi.nlm.nih.gov/pubmed/17635941?ordinalpos=2&itool=EntrezSystem2.PEntrez.Pubmed.Pubmed_ResultsPanel.Pubmed_RVDocSum) (2007). Adenovirus-mediated delivery of a dominant-negative estrogen receptor gene in uterine leiomyoma cells abrogates estrogen- and progesterone-regulated gene expression. *Journal of Clinical Endocrinology and Metabolism* **92**:3949-3957.

153. [Katoh M, Katoh M.](http://www.ncbi.nlm.nih.gov/pubmed/17143535?ordinalpos=3&itool=EntrezSystem2.PEntrez.Pubmed.Pubmed_ResultsPanel.Pubmed_RVDocSum) (2007). Notch signaling in gastrointestinal tract (review). *International journal of oncology* **30**: 247-251.

154. [Subramanian S, West RB, Marinelli RJ, Nielsen TO, Rubin BP, Goldblum JR, Patel RM, Zhu S, Montgomery K, Ng TL, Corless CL, Heinrich MC, van de Rijn M.](http://www.ncbi.nlm.nih.gov/pubmed/15920699?ordinalpos=4&itool=EntrezSystem2.PEntrez.Pubmed.Pubmed_ResultsPanel.Pubmed_RVDocSum) (2005). The gene expression profile of extraskeletal myxoid chondrosarcoma. *Journal of Pathology* **206**: 433-444.

155. [Kato K, Horiuchi S, Takahashi A, Ueoka Y, Arima T, Matsuda T, Kato H, Nishida Ji J, Nakabeppu Y, Wake N.](http://www.ncbi.nlm.nih.gov/pubmed/11781307?ordinalpos=5&itool=EntrezSystem2.PEntrez.Pubmed.Pubmed_ResultsPanel.Pubmed_RVDocSum) (2002). Contribution of estrogen receptor alpha to oncogenic K-Ras-mediated NIH3T3 cell transformation and its implication for escape from senescence by modulating the p53 pathway. *Journal of Biological Chemistry* **277**: 11217-11224.

**Gene 54: PRNP**

**Official symbol: PRNP**

**Official Full Name:** prion protein (p27-30)

**Also known as:** CJD; GSS; PrP; ASCR; PRIP; PrPc; CD230; MGC26679; PrP27-30; PrP33-35C

**References:**

156. [Kaiser S, Park YK, Franklin JL, Halberg RB, Yu M, Jessen WJ, Freudenberg J, Chen X, Haigis K, Jegga AG, Kong S, Sakthivel B, Xu H, Reichling T, Azhar M, Boivin GP, Roberts RB, Bissahoyo AC, Gonzales F, Bloom GC, Eschrich S, Carter SL, Aronow JE, Kleimeyer J, Kleimeyer M, Ramaswamy V, Settle SH, Boone B, Levy S, Graff JM, Doetschman T, Groden J, Dove WF, Threadgill DW, Yeatman TJ, Coffey RJ Jr, Aronow BJ.](http://www.ncbi.nlm.nih.gov/pubmed/17615082?ordinalpos=1&itool=EntrezSystem2.PEntrez.Pubmed.Pubmed_ResultsPanel.Pubmed_RVDocSum) (2007). Transcriptional recapitulation and subversion of embryonic colon development by mouse colon tumor models and human colon cancer. *Genome biology* **8**: R131.

157. [Norstrom EM, Ciaccio MF, Rassbach B, Wollmann R, Mastrianni JA.](http://www.ncbi.nlm.nih.gov/pubmed/17182694?ordinalpos=4&itool=EntrezSystem2.PEntrez.Pubmed.Pubmed_ResultsPanel.Pubmed_RVDocSum) (2007). Cytosolic prion protein toxicity is independent of cellular prion protein expression and prion propagation. *Journal of Virology* **81**: 2831-2837.

158. [van Santen HM, Aronson DC, Vulsma T, Tummers RF, Geenen MM, de Vijlder JJ, van den Bos C.](http://www.ncbi.nlm.nih.gov/pubmed/15251165?ordinalpos=10&itool=EntrezSystem2.PEntrez.Pubmed.Pubmed_ResultsPanel.Pubmed_RVDocSum) (2004). Frequent adverse events after treatment for childhood-onset differentiated thyroid carcinoma: a single institute experience. *European Journal of Cancer* **40**: 1743-1751.

159. [Diarra-Mehrpour M, Arrabal S, Jalil A, Pinson X, Gaudin C, Piétu G, Pitaval A, Ripoche H, Eloit M, Dormont D, Chouaib S.](http://www.ncbi.nlm.nih.gov/pubmed/14744790?ordinalpos=11&itool=EntrezSystem2.PEntrez.Pubmed.Pubmed_ResultsPanel.Pubmed_RVDocSum) (2004). Prion protein prevents human breast carcinoma cell line from tumor necrosis factor alpha-induced cell death. Cancer Research 64: 719-727.

**Gene 55: PDK4**

**Official symbol: PDK4**

**Official Full Name:** pyruvate dehydrogenase kinase, isozyme 4

**Also known as:** FLJ40832

**References:**

160. [Nagasawa M, Akasaka Y, Ide T, Hara T, Kobayashi N, Utsumi M, Murakami K.](http://www.ncbi.nlm.nih.gov/pubmed/17904533?ordinalpos=1&itool=EntrezSystem2.PEntrez.Pubmed.Pubmed_ResultsPanel.Pubmed_RVDocSum) (2007). Highly sensitive upregulation of apolipoprotein A-IV by peroxisome proliferator-activated receptor alpha (PPARalpha) agonist in human hepatoma cells. [*Biochemical Pharmacology*](http://www.ncbi.nlm.nih.gov/sites/entrez?Db=journals&Cmd=ShowDetailView&TermToSearch=1015) **74**: 1738-1746.

161. [Roche TE, Hiromasa Y.](http://www.ncbi.nlm.nih.gov/pubmed/17310282?ordinalpos=2&itool=EntrezSystem2.PEntrez.Pubmed.Pubmed_ResultsPanel.Pubmed_RVDocSum) (2007). Pyruvate dehydrogenase kinase regulatory mechanisms and inhibition in treating diabetes, heart ischemia, and cancer. *Cellular and Molecular Life Sciences* **64**: 830-849. .

162. [Zhang Y, Ma K, Sadana P, Chowdhury F, Gaillard S, Wang F, McDonnell DP, Unterman TG, Elam MB, Park EA.](http://www.ncbi.nlm.nih.gov/pubmed/17079227?ordinalpos=3&itool=EntrezSystem2.PEntrez.Pubmed.Pubmed_ResultsPanel.Pubmed_RVDocSum) (2006). Estrogen-related receptors stimulate pyruvate dehydrogenase kinase isoform 4 gene expression. *Journal of Biological Chemistry* **281**: 39897-39906.

163. [Savkur RS, Bramlett KS, Michael LF, Burris TP.](http://www.ncbi.nlm.nih.gov/pubmed/15721319?ordinalpos=4&itool=EntrezSystem2.PEntrez.Pubmed.Pubmed_ResultsPanel.Pubmed_RVDocSum) (2005). Regulation of pyruvate dehydrogenase kinase expression by the farnesoid X receptor. *Biochemical and Biophysical Research Communications* **329**: 391-396.

**Gene 56: APOD**

**Official symbol: APOD**

**Official Full Name:** apolipoprotein D

**Also known as:**

**References:**

164. [Ashida S, Nakagawa H, Katagiri T, Furihata M, Iiizumi M, Anazawa Y, Tsunoda T, Takata R, Kasahara K, Miki T, Fujioka T, Shuin T, Nakamura Y.](http://www.ncbi.nlm.nih.gov/pubmed/15342375?ordinalpos=1&itool=EntrezSystem2.PEntrez.Pubmed.Pubmed_ResultsPanel.Pubmed_RVDocSum) (2004). Molecular features of the transition from prostatic intraepithelial neoplasia (PIN) to prostate cancer: genome-wide gene-expression profiles of prostate cancers and PINs. *Cancer Research* **64**: 5963-5972.

165. [Rodríguez JC, Díaz M, González LO, Sánchez J, Sánchez MT, Merino AM, Vizoso F.](http://www.ncbi.nlm.nih.gov/pubmed/12678535?ordinalpos=3&itool=EntrezSystem2.PEntrez.Pubmed.Pubmed_ResultsPanel.Pubmed_RVDocSum) (2000). Apolipoprotein D expression in benign and malignant prostate tissues*. International journal of surgical investigation* **2**: 319-326.

166. [Simard J, Veilleux R, de Launoit Y, Haagensen DE, Labrie F.](http://www.ncbi.nlm.nih.gov/pubmed/1868457?ordinalpos=11&itool=EntrezSystem2.PEntrez.Pubmed.Pubmed_ResultsPanel.Pubmed_RVDocSum) (1991). Stimulation of apolipoprotein D secretion by steroids coincides with inhibition of cell proliferation in human LNCaP prostate cancer cells. *Cancer Research* **51**: 4336-4341.

**Gene 57: HERPUD1**

**Official symbol: HERPUD1**

**Official Full Name:** homocysteine-inducible, endoplasmic reticulum stress-inducible, ubiquitin-like domain member 1

**Also known as:** SUP; HERP; Mif1; KIAA0025

**References:**

167. [Joo JH, Liao G, Collins JB, Grissom SF, Jetten AM.](http://www.ncbi.nlm.nih.gov/pubmed/17699800?ordinalpos=1&itool=EntrezSystem2.PEntrez.Pubmed.Pubmed_ResultsPanel.Pubmed_RVDocSum) (2007). Farnesol-induced apoptosis in human lung carcinoma cells is coupled to the endoplasmic reticulum stress response. *Cancer Research* **67**: 7929-7936.

168. [Hong SH, Kim J, Kim JM, Lee SY, Shin DS, Son KH, Han DC, Sung YK, Kwon BM.](http://www.ncbi.nlm.nih.gov/pubmed/17606223?ordinalpos=2&itool=EntrezSystem2.PEntrez.Pubmed.Pubmed_ResultsPanel.Pubmed_RVDocSum) (2007). Apoptosis induction of 2'-hydroxycinnamaldehyde as a proteasome inhibitor is associated with ER stress and mitochondrial perturbation in cancer cells. [*Biochemical pharmacology*](http://www.ncbi.nlm.nih.gov/sites/entrez?Db=journals&Cmd=ShowDetailView&TermToSearch=1015) **74**: 557-565.

169. [Hendriksen PJ, Dits NF, Kokame K, Veldhoven A, van Weerden WM, Bangma CH, Trapman J, Jenster G.](http://www.ncbi.nlm.nih.gov/pubmed/16707422?ordinalpos=3&itool=EntrezSystem2.PEntrez.Pubmed.Pubmed_ResultsPanel.Pubmed_RVDocSum) (2006). Evolution of the androgen receptor pathway during progression of prostate cancer. *Cancer Research* **66**: 5012-5020.

170. [García J, Castrillo JL.](http://www.ncbi.nlm.nih.gov/pubmed/15450385?ordinalpos=4&itool=EntrezSystem2.PEntrez.Pubmed.Pubmed_ResultsPanel.Pubmed_RVDocSum) (2004). Differential display RT-PCR analysis of human choriocarcinoma cell lines and normal term trophoblast cells: identification of new genes expressed in placenta. *Placenta* **25**: 684-693.

171. [Segawa T, Nau ME, Xu LL, Chilukuri RN, Makarem M, Zhang W, Petrovics G, Sesterhenn IA, McLeod DG, Moul JW, Vahey M, Srivastava S.](http://www.ncbi.nlm.nih.gov/pubmed/12483528?ordinalpos=5&itool=EntrezSystem2.PEntrez.Pubmed.Pubmed_ResultsPanel.Pubmed_RVDocSum) (2002). Androgen-induced expression of endoplasmic reticulum (ER) stress response genes in prostate cancer cells. *Oncogene* **21**: 8749-8758.

**Gene 58: FSTL1**

**Official symbol: FSTL1**

**Official Full Name:** follistatin-like 1

**Also known as:** FRP; FSL1

**References:**

172. [Hodgson G, Hager JH, Volik S, Hariono S, Wernick M, Moore D, Nowak N, Albertson DG, Pinkel D, Collins C, Hanahan D, Gray JW.](http://www.ncbi.nlm.nih.gov/pubmed/11694878?ordinalpos=13&itool=EntrezSystem2.PEntrez.Pubmed.Pubmed_ResultsPanel.Pubmed_RVDocSum) (2001). Genome scanning with array CGH delineates regional alterations in mouse islet carcinomas. *Nature Genetics* **29**: 459-464.

**Gene 59: HSPCB**

**Official symbol:** HSP90AB1

**Official Full Name:** heat shock protein 90kDa alpha (cytosolic), class B member 1

**Also known as:** HSPC2; HSPCB; D6S182; HSP90B; FLJ26984; HSP90-BETA

**References:**

173. [Chan CT, Paulmurugan R, Gheysens OS, Kim J, Chiosis G, Gambhir SS.](http://www.ncbi.nlm.nih.gov/pubmed/18172314?ordinalpos=2&itool=EntrezSystem2.PEntrez.Pubmed.Pubmed_ResultsPanel.Pubmed_RVDocSum) (2008). Molecular imaging of the efficacy of heat shock protein 90 inhibitors in living subjects. *Cancer Research* **68**: 216-226.

174. [Negroni L, Samson M, Guigonis JM, Rossi B, Pierrefite-Carle V, Baudoin C.](http://www.ncbi.nlm.nih.gov/pubmed/17938268?ordinalpos=3&itool=EntrezSystem2.PEntrez.Pubmed.Pubmed_ResultsPanel.Pubmed_RVDocSum) (2007). Treatment of colon cancer cells using the cytosine deaminase/5-fluorocytosine suicide system induces apoptosis, modulation of the proteome, and Hsp90beta phosphorylation. *Molecular Cancer Therapeutics* **6**: 2747-2756.

175. [Sato T, Susuki S, Suico MA, Miyata M, Ando Y, Mizuguchi M, Takeuchi M, Dobashi M, Shuto T, Kai H.](http://www.ncbi.nlm.nih.gov/pubmed/17431395?ordinalpos=4&itool=EntrezSystem2.PEntrez.Pubmed.Pubmed_ResultsPanel.Pubmed_RVDocSum) (2007). Endoplasmic reticulum quality control regulates the fate of transthyretin variants in the cell. *EMBO Journal* **26**: 2501-2512.

176. [Yu X, Harris SL, Levine AJ.](http://www.ncbi.nlm.nih.gov/pubmed/16651434?ordinalpos=6&itool=EntrezSystem2.PEntrez.Pubmed.Pubmed_ResultsPanel.Pubmed_RVDocSum) (2006). The regulation of exosome secretion: a novel function of the p53 protein. *Cancer Research* **66**: 4795-4801.

177. [Hayashi E, Kuramitsu Y, Okada F, Fujimoto M, Zhang X, Kobayashi M, Iizuka N, Ueyama Y, Nakamura K.](http://www.ncbi.nlm.nih.gov/pubmed/15712240?ordinalpos=7&itool=EntrezSystem2.PEntrez.Pubmed.Pubmed_ResultsPanel.Pubmed_RVDocSum) (2005). Proteomic profiling for cancer progression: Differential display analysis for the expression of intracellular proteins between regressive and progressive cancer cell lines. *Proteomics* **5**: 1024-1032.

**Gene 60: GSTM2**

**Official symbol: GSTM2**

**Official Full Name:** glutathione S-transferase M2 (muscle)

**Also known as:** GST4; GSTM; GTHMUS; GSTM2-2; MGC117303

**References:**

178. [Tijhuis MJ, Visker MH, Aarts JM, Peters WH, Roelofs HM, den Camp LO, Rietjens IM, Boerboom AM, Nagengast FM, Kok FJ, Kampman E.](http://www.ncbi.nlm.nih.gov/pubmed/17071629?ordinalpos=1&itool=EntrezSystem2.PEntrez.Pubmed.Pubmed_ResultsPanel.Pubmed_RVDocSum) (2007). Glutathione S-transferase phenotypes in relation to genetic variation and fruit and vegetable consumption in an endoscopy-based population. *Carcinogenesis* **28**: 848-857.

179. [Ricci G, De Maria F, Antonini G, Turella P, Bullo A, Stella L, Filomeni G, Federici G, Caccuri AM.](http://www.ncbi.nlm.nih.gov/pubmed/15888444?ordinalpos=3&itool=EntrezSystem2.PEntrez.Pubmed.Pubmed_ResultsPanel.Pubmed_RVDocSum) (2005). 7-Nitro-2,1,3-benzoxadiazole derivatives, a new class of suicide inhibitors for glutathione S-transferases. Mechanism of action of potential anticancer drugs. *Journal of Biological Chemistry* **280**: 26397-26405.

180. [Ebert MN, Klinder A, Peters WH, Schäferhenrich A, Sendt W, Scheele J, Pool-Zobel BL.](http://www.ncbi.nlm.nih.gov/pubmed/12896903?ordinalpos=6&itool=EntrezSystem2.PEntrez.Pubmed.Pubmed_ResultsPanel.Pubmed_RVDocSum) (2003). Expression of glutathione S-transferases (GSTs) in human colon cells and inducibility of GSTM2 by butyrate. *Carcinogenesis* **24**: 1637-1644.

**Gene 61: PTN**

**Official symbol: PTN**

**Official Full Name:** pleiotrophin

**Also known as:** HARP; HBNF; HBGF8; NEGF1

**References:**

181. [Mikelis C, Koutsioumpa M, Papadimitriou E.](http://www.ncbi.nlm.nih.gov/pubmed/18221061?ordinalpos=1&itool=EntrezSystem2.PEntrez.Pubmed.Pubmed_ResultsPanel.Pubmed_RVDocSum) (2007). Pleiotrophin as a possible new target for angiogenesis-related diseases and cancer. *Recent Patents on Anti-cancer Drug Discovery* **2**: 175-186.

182. [Calvet L, Geoerger B, Regairaz M, Opolon P, Machet L, Morizet J, Joseph JM, Elie N, Vassal G.](http://www.ncbi.nlm.nih.gov/pubmed/16501609?ordinalpos=1&itool=EntrezSystem2.PEntrez.Pubmed.Pubmed_ResultsPanel.Pubmed_RVDocSum) (2006). Pleiotrophin, a candidate gene for poor tumor vasculature and in vivo neuroblastoma sensitivity to irinotecan. *Oncogene* **25**: 3150-3159.

183. [Krauss O, Ernst J, Kauschke M, Stolzenburg JU, Weissflog G, Schwarz R.](http://www.ncbi.nlm.nih.gov/pubmed/16465520?ordinalpos=2&itool=EntrezSystem2.PEntrez.Pubmed.Pubmed_ResultsPanel.Pubmed_RVDocSum) (2006). Patients after prostatectomy. Psychiatric comorbidity, need for psychooncological treatment and quality of life. *Der Urologe. Ausg. A* **45**: 482-488.

184. [Yamashita S, Wakazono K, Nomoto T, Tsujino Y, Kuramoto T, Ushijima T.](http://www.ncbi.nlm.nih.gov/pubmed/16079240?ordinalpos=3&itool=EntrezSystem2.PEntrez.Pubmed.Pubmed_ResultsPanel.Pubmed_RVDocSum) (2005). Expression quantitative trait loci analysis of 13 genes in the rat prostate. *Genetics* **171**: 1231-1238.

**Gene 62: ERG**

**Official symbol: ERG**

**Official Full Name:** v-ets erythroblastosis virus E26 oncogene homolog (avian)

**Also known as:** p55; erg-3

**References:**

185. [Attard G, Clark J, Ambroisine L, Fisher G, Kovacs G, Flohr P, Berney D, Foster CS, Fletcher A, Gerald WL, Moller H, Reuter V, De Bono JS, Scardino P, Cuzick J, Cooper CS; Transatlantic Prostate Group.](http://www.ncbi.nlm.nih.gov/pubmed/17637754?ordinalpos=22&itool=EntrezSystem2.PEntrez.Pubmed.Pubmed_ResultsPanel.Pubmed_RVDocSum) (2008). Duplication of the fusion of TMPRSS2 to ERG sequences identifies fatal human prostate cancer. *Oncogene* **27**: 253-263.

186. [Clark J, Attard G, Jhavar S, Flohr P, Reid A, De-Bono J, Eeles R, Scardino P, Cuzick J, Fisher G, Parker MD, Foster CS, Berney D, Kovacs G, Cooper CS.](http://www.ncbi.nlm.nih.gov/pubmed/17922029?ordinalpos=15&itool=EntrezSystem2.PEntrez.Pubmed.Pubmed_ResultsPanel.Pubmed_RVDocSum) (2007). Complex patterns of ETS gene alteration arise during cancer development in the human prostate. *Oncogene* Epub ahead of print.

187. [Lapointe J, Li C, Giacomini CP, Salari K, Huang S, Wang P, Ferrari M, Hernandez-Boussard T, Brooks JD, Pollack JR.](http://www.ncbi.nlm.nih.gov/pubmed/17875689?ordinalpos=16&itool=EntrezSystem2.PEntrez.Pubmed.Pubmed_ResultsPanel.Pubmed_RVDocSum) (2007). Genomic profiling reveals alternative genetic pathways of prostate tumorigenesis. *Cancer Research* 67:8504-8510.

188. [Tomlins SA, Laxman B, Dhanasekaran SM, Helgeson BE, Cao X, Morris DS, Menon A, Jing X, Cao Q, Han B, Yu J, Wang L, Montie JE, Rubin MA, Pienta KJ, Roulston D, Shah RB, Varambally S, Mehra R, Chinnaiyan AM.](http://www.ncbi.nlm.nih.gov/pubmed/17671502?ordinalpos=20&itool=EntrezSystem2.PEntrez.Pubmed.Pubmed_ResultsPanel.Pubmed_RVDocSum) (2007). Distinct classes of chromosomal rearrangements create oncogenic ETS gene fusions in prostate cancer. *Nature* **448**: 595-599.

189. [Tomlins SA, Rhodes DR, Perner S, Dhanasekaran SM, Mehra R, Sun XW, Varambally S, Cao X, Tchinda J, Kuefer R, Lee C, Montie JE, Shah RB, Pienta KJ, Rubin MA, Chinnaiyan AM.](http://www.ncbi.nlm.nih.gov/pubmed/16254181?ordinalpos=54&itool=EntrezSystem2.PEntrez.Pubmed.Pubmed_ResultsPanel.Pubmed_RVDocSum) (2005). Recurrent fusion of TMPRSS2 and ETS transcription factor genes in prostate cancer. *Science* **310**: 644-648

190. [Marx J.](http://www.ncbi.nlm.nih.gov/pubmed/16254158?ordinalpos=55&itool=EntrezSystem2.PEntrez.Pubmed.Pubmed_ResultsPanel.Pubmed_RVDocSum) (2005). Medicine. Fused genes may help explain the origins of prostate cancer. *Science* **310**: 603.

**Gene 63: CTGF**

**Official symbol: CTGF**

**Official Full Name:** connective tissue growth factor

**Also known as:** CCN2; NOV2; HCS24; IGFBP8; MGC102839

**References:**

191. [Yang F, Tuxhorn JA, Ressler SJ, McAlhany SJ, Dang TD, Rowley DR.](http://www.ncbi.nlm.nih.gov/pubmed/16204060?ordinalpos=1&itool=EntrezSystem2.PEntrez.Pubmed.Pubmed_ResultsPanel.Pubmed_RVDocSum) (2005). Stromal expression of connective tissue growth factor promotes angiogenesis and prostate cancer tumorigenesis. *Cancer Research* **65**: 8887-8895.

192. [Shimizu T, Okayama A, Inoue T, Takeda K.](http://www.ncbi.nlm.nih.gov/pubmed/16012728?ordinalpos=2&itool=EntrezSystem2.PEntrez.Pubmed.Pubmed_ResultsPanel.Pubmed_RVDocSum) (2005). Analysis of gene expression during staurosporine-induced neuronal differentiation of human prostate cancer cells. *Oncology Reports* **14**: 441-448.

193. [Untergasser G, Gander R, Lilg C, Lepperdinger G, Plas E, Berger P.](http://www.ncbi.nlm.nih.gov/pubmed/15610763?ordinalpos=3&itool=EntrezSystem2.PEntrez.Pubmed.Pubmed_ResultsPanel.Pubmed_RVDocSum) (2005). Profiling molecular targets of TGF-beta1 in prostate fibroblast-to-myofibroblast transdifferentiation.*Mechanisms of Ageing and Development* **126**: 59-69.

194. [Gervaz P, Hennig R, Buechler M, Soravia C, Brigstock DR, Morel P, Egger JF, Friess H.](http://www.ncbi.nlm.nih.gov/pubmed/12974179?ordinalpos=4&itool=EntrezSystem2.PEntrez.Pubmed.Pubmed_ResultsPanel.Pubmed_RVDocSum)(2003). Long-term expression of fibrogenic cytokines in radiation-induced damage to the internal anal sphincter. *Swiss Surgery* **9**: 193-197.

195. [Perbal B.](http://www.ncbi.nlm.nih.gov/pubmed/11322167?ordinalpos=5&itool=EntrezSystem2.PEntrez.Pubmed.Pubmed_ResultsPanel.Pubmed_RVDocSum) (2001). NOV (nephroblastoma overexpressed) and the CCN family of genes: structural and functional issues. *Molecular Pathology* **54**: 57-79.

**Gene 64: GUCY1A3**

**Official symbol:** GUCY1A3

**Official Full Name:** guanylate cyclase 1, soluble, alpha 3

**Also known as:** GUCA3; GC-SA3; GUC1A3; GUCSA3

**References:**

196. [Dong Y, Zhang H, Gao AC, Marshall JR, Ip C.](http://www.ncbi.nlm.nih.gov/pubmed/16020662?ordinalpos=1&itool=EntrezSystem2.PEntrez.Pubmed.Pubmed_ResultsPanel.Pubmed_RVDocSum) (2005). Androgen receptor signaling intensity is a key factor in determining the sensitivity of prostate cancer cells to selenium inhibition of growth and cancer-specific biomarkers. *Molecular Cancer Therapeutics* **4**:1047-1055.

197. [Saino M, Maruyama T, Sekiya T, Kayama T, Murakami Y.](http://www.ncbi.nlm.nih.gov/pubmed/15201957?ordinalpos=2&itool=EntrezSystem2.PEntrez.Pubmed.Pubmed_ResultsPanel.Pubmed_RVDocSum) (2004). Inhibition of angiogenesis in human glioma cell lines by antisense RNA from the soluble guanylate cyclase genes, GUCY1A3 and GUCY1B3. *Oncology Reports* **12**: 47-52.

**Gene 65: MT1F**

**Official symbol: MT1F**

**Official Full Name:** metallothionein 1F

**Also known as:** MT1; MGC32732

**References:**

198. [Lee S, Bang S, Song K, Lee I.](http://www.ncbi.nlm.nih.gov/pubmed/16969489?ordinalpos=1&itool=EntrezSystem2.PEntrez.Pubmed.Pubmed_ResultsPanel.Pubmed_RVDocSum) (2006). Differential expression in normal-adenoma-carcinoma sequence suggests complex molecular carcinogenesis in colon. *Oncology Reports* **16**: 747-754.

199. [Lu DD, Chen YC, Zhang XR, Cao XR, Jiang HY, Yao L.](http://www.ncbi.nlm.nih.gov/pubmed/15369632?ordinalpos=2&itool=EntrezSystem2.PEntrez.Pubmed.Pubmed_ResultsPanel.Pubmed_RVDocSum) (2003). The relationship between metallothionein-1F (MT1F) gene and hepatocellular carcinoma. *Yale Journal of Biology and Medicine* **76**: 55-62.

**Gene 66: TIMP3**

**Official symbol: TIMP3**

**Official Full Name:** TIMP metallopeptidase inhibitor 3

**Also known as:** SFD; K222; K222TA2; HSMRK222

**References:**

200. [Rouprêt M, Hupertan V, Yates DR, Catto JW, Rehman I, Meuth M, Ricci S, Lacave R, Cancel-Tassin G, de la Taille A, Rozet F, Cathelineau X, Vallancien G, Hamdy FC, Cussenot O.](http://www.ncbi.nlm.nih.gov/pubmed/17363525?ordinalpos=2&itool=EntrezSystem2.PEntrez.Pubmed.Pubmed_ResultsPanel.Pubmed_RVDocSum) (2007). Molecular detection of localized prostate cancer using quantitative methylation-specific PCR on urinary cells obtained following prostate massage. *Clinical Cancer Research* **13**: 1720-1725.

201. [Hoque MO, Topaloglu O, Begum S, Henrique R, Rosenbaum E, Van Criekinge W, Westra WH, Sidransky D.](http://www.ncbi.nlm.nih.gov/pubmed/16170165?ordinalpos=3&itool=EntrezSystem2.PEntrez.Pubmed.Pubmed_ResultsPanel.Pubmed_RVDocSum) (2005). Quantitative methylation-specific polymerase chain reaction gene patterns in urine sediment distinguish prostate cancer patients from control subjects. *Journal of Clinical Oncology* **23**: 6569-6575.

202. [Riddick AC, Shukla CJ, Pennington CJ, Bass R, Nuttall RK, Hogan A, Sethia KK, Ellis V, Collins AT, Maitland NJ, Ball RY, Edwards DR.](http://www.ncbi.nlm.nih.gov/pubmed/15928670?ordinalpos=4&itool=EntrezSystem2.PEntrez.Pubmed.Pubmed_ResultsPanel.Pubmed_RVDocSum) (2005). Identification of degradome components associated with prostate cancer progression by expression analysis of human prostatic tissues. *British Journal of Cancer* **92**: 2171-2180.

203. [Jerónimo C, Henrique R, Hoque MO, Mambo E, Ribeiro FR, Varzim G, Oliveira J, Teixeira MR, Lopes C, Sidransky D.](http://www.ncbi.nlm.nih.gov/pubmed/15623627?ordinalpos=5&itool=EntrezSystem2.PEntrez.Pubmed.Pubmed_ResultsPanel.Pubmed_RVDocSum) (2004). A quantitative promoter methylation profile of prostate cancer. *Clinical Cancer Research* **10**: 8472-8478.

204. [Yegnasubramanian S, Kowalski J, Gonzalgo ML, Zahurak M, Piantadosi S, Walsh PC, Bova GS, De Marzo AM, Isaacs WB, Nelson WG.](http://www.ncbi.nlm.nih.gov/pubmed/15026333?ordinalpos=6&itool=EntrezSystem2.PEntrez.Pubmed.Pubmed_ResultsPanel.Pubmed_RVDocSum) (2004). Hypermethylation of CpG islands in primary and metastatic human prostate cancer. *Cancer Research* **64**: 1975-1986.

205. [Karan D, Lin FC, Bryan M, Ringel J, Moniaux N, Lin MF, Batra SK.](http://www.ncbi.nlm.nih.gov/pubmed/14532978?ordinalpos=7&itool=EntrezSystem2.PEntrez.Pubmed.Pubmed_ResultsPanel.Pubmed_RVDocSum) (2003). Expression of ADAMs (a disintegrin and metalloproteases) and TIMP-3 (tissue inhibitor of metalloproteinase-3) in human prostatic adenocarcinomas. *International Journal of Oncology* **23**: 1365-1371.

**Gene 67: LDHB**

**Official symbol: LDHB**

**Official Full Name:** lactate dehydrogenase B

**Also known as:** LDH-H; TRG-5

**References:**

206. [Leiblich A, Cross SS, Catto JW, Phillips JT, Leung HY, Hamdy FC, Rehman I.](http://www.ncbi.nlm.nih.gov/pubmed/16547507?ordinalpos=3&itool=EntrezSystem2.PEntrez.Pubmed.Pubmed_ResultsPanel.Pubmed_RVDocSum) (2006). Lactate dehydrogenase-B is silenced by promoter hypermethylation in human prostate cancer. *Oncogene* **25**: 2953-2960.

207. [Maekawa M, Taniguchi T, Ishikawa J, Sugimura H, Sugano K, Kanno T.](http://www.ncbi.nlm.nih.gov/pubmed/12928234?ordinalpos=5&itool=EntrezSystem2.PEntrez.Pubmed.Pubmed_ResultsPanel.Pubmed_RVDocSum) (2003). Promoter hypermethylation in cancer silences LDHB, eliminating lactate dehydrogenase isoenzymes 1-4. *Clinical Chemistry* **49**: 1518-1520.

208. [Rodriguez S, Jafer O, Goker H, Summersgill BM, Zafarana G, Gillis AJ, van Gurp RJ, Oosterhuis JW, Lu YJ, Huddart R, Cooper CS, Clark J, Looijenga LH, Shipley JM.](http://www.ncbi.nlm.nih.gov/pubmed/12660824?ordinalpos=6&itool=EntrezSystem2.PEntrez.Pubmed.Pubmed_ResultsPanel.Pubmed_RVDocSum) (2003). Expression profile of genes from 12p in testicular germ cell tumors of adolescents and adults associated with i(12p) and amplification at 12p11.2-p12.1. *Oncogene* **22**: 1880-1891.

209. [Holtkamp B, Cramer M, Rajewsky K.](http://www.ncbi.nlm.nih.gov/pubmed/6196194?ordinalpos=9&itool=EntrezSystem2.PEntrez.Pubmed.Pubmed_ResultsPanel.Pubmed_RVDocSum) (1983). Somatic variation of H-2Kk expression and structure in a T-cell lymphoma: instability, stabilization, high production and structural mutation. *EMBO Journal* **2**: 1943-1951.

**Gene 68: RNASE4**

**Official symbol: RNASE4**

**Official Full Name:** ribonuclease, RNase A family, 4

**Also known as:** RNS4; MGC9306

**References:**

**Gene 69: ANPEP**

**Official symbol: ANPEP**

**Official Full Name:** alanyl (membrane) aminopeptidase (aminopeptidase N, aminopeptidase M, microsomal aminopeptidase, CD13, p150)

**Also known as:** APN; CD13; LAP1; PEPN; gp150

**References:**

210. [Wiese AH, Auer J, Lassmann S, Nährig J, Rosenberg R, Höfler H, Rüger R, Werner M.](http://www.ncbi.nlm.nih.gov/pubmed/17936523?ordinalpos=1&itool=EntrezSystem2.PEntrez.Pubmed.Pubmed_ResultsPanel.Pubmed_RVDocSum) (2007). Identification of gene signatures for invasive colorectal tumor cells. *Cancer Detection and Prevention* **31**: 282-295.

211. [Razvi MH, Peng D, Dar AA, Powell SM, Frierson HF Jr, Moskaluk CA, Washington K, El-Rifai W.](http://www.ncbi.nlm.nih.gov/pubmed/17636545?ordinalpos=2&itool=EntrezSystem2.PEntrez.Pubmed.Pubmed_ResultsPanel.Pubmed_RVDocSum) (2007). Transcriptional oncogenomic hot spots in Barrett's adenocarcinomas: serial analysis of gene expression. *Genes, Chromosomes & Cancer* **46**: 914-928.

212. [Kern W, Kohlmann A, Schoch C, Schnittger S, Haferlach T.](http://www.ncbi.nlm.nih.gov/pubmed/17041886?ordinalpos=3&itool=EntrezSystem2.PEntrez.Pubmed.Pubmed_ResultsPanel.Pubmed_RVDocSum) (2006). Comparison of mRNA abundance quantified by gene expression profiling and percentage of positive cells using immunophenotyping for diagnostic antigens in acute and chronic leukemias. *Cancer* **107**: 2401-2407.

**Gene 70: CAV1**

**Official symbol: CAV1**

**Official Full Name:** caveolin 1, caveolae protein, 22kDa

**Also known as:** CAV; VIP21; MSTP085

**References:**

213. [Karam JA, Lotan Y, Roehrborn CG, Ashfaq R, Karakiewicz PI, Shariat SF.](http://www.ncbi.nlm.nih.gov/pubmed/17299799?ordinalpos=4&itool=EntrezSystem2.PEntrez.Pubmed.Pubmed_ResultsPanel.Pubmed_RVDocSum) (2007). Caveolin-1 overexpression is associated with aggressive prostate cancer recurrence. *Prostate* **67**: 614-622.

214. [Llorente A, de Marco MC, Alonso MA.](http://www.ncbi.nlm.nih.gov/pubmed/15466889?ordinalpos=13&itool=EntrezSystem2.PEntrez.Pubmed.Pubmed_ResultsPanel.Pubmed_RVDocSum) (2004). Caveolin-1 and MAL are located on prostasomes secreted by the prostate cancer PC-3 cell line. *Journal of Cell Science* **117**: 5343-5351.

215. [Sowa G, Pypaert M, Fulton D, Sessa WC.](http://www.ncbi.nlm.nih.gov/pubmed/12743374?ordinalpos=18&itool=EntrezSystem2.PEntrez.Pubmed.Pubmed_ResultsPanel.Pubmed_RVDocSum) (2003). The phosphorylation of caveolin-2 on serines 23 and 36 modulates caveolin-1-dependent caveolae formation. *Proceedings of the National Academy of Sciences of the United States of America* **100**: 6511-6516.

216. [Timme TL, Goltsov A, Tahir S, Li L, Wang J, Ren C, Johnston RN, Thompson TC.](http://www.ncbi.nlm.nih.gov/pubmed/10918582?ordinalpos=35&itool=EntrezSystem2.PEntrez.Pubmed.Pubmed_ResultsPanel.Pubmed_RVDocSum) (2000). Caveolin-1 is regulated by c-myc and suppresses c-myc-induced apoptosis. *Oncogene* **19**: 3256-3265.

217. [Yang G, Truong LD, Wheeler TM, Thompson TC.](http://www.ncbi.nlm.nih.gov/pubmed/10582690?ordinalpos=36&itool=EntrezSystem2.PEntrez.Pubmed.Pubmed_ResultsPanel.Pubmed_RVDocSum)(1999). Caveolin-1 expression in clinically confined human prostate cancer: a novel prognostic marker. *Cancer Research* **59**: 5719-5723.

218. [Nasu Y, Timme TL, Yang G, Bangma CH, Li L, Ren C, Park SH, DeLeon M, Wang J, Thompson TC.](http://www.ncbi.nlm.nih.gov/pubmed/9734401?ordinalpos=41&itool=EntrezSystem2.PEntrez.Pubmed.Pubmed_ResultsPanel.Pubmed_RVDocSum) (1998). Suppression of caveolin expression induces androgen sensitivity in metastatic androgen-insensitive mouse prostate cancer cells. *Nature Medicine* **4**: 1062-1064.

**Gene 71: TM9SF2**

**Official symbol: TM9SF2**

**Official Full Name:** transmembrane 9 superfamily member 2

**Also known as:** P76; FLJ26287; MGC117391

**References:**

**Gene 72: H11**

**Official symbol:** HSPB8

**Official Full Name:** heat shock 22kDa protein 8

**Also known as:** H11; HMN2; CMT2L; DHMN2; E2IG1; HMN2A; HSP22

**References:**

219. [Trent S, Yang C, Li C, Lynch M, Schmidt EV.](http://www.ncbi.nlm.nih.gov/pubmed/18006821?ordinalpos=2&itool=EntrezSystem2.PEntrez.Pubmed.Pubmed_ResultsPanel.Pubmed_RVDocSum) (2007). Heat shock protein B8, a cyclin-dependent kinase-independent cyclin D1 target gene, contributes to its effects on radiation sensitivity. *Cancer Research* **67**: 10774-10781.

220. [Schlomm T, Luebke AM, Sültmann H, Hellwinkel OJ, Sauer U, Poustka A, David KA, Chun FK, Haese A, Graefen M, Erbersdobler A, Huland H.](http://www.ncbi.nlm.nih.gov/pubmed/16077921?ordinalpos=1&itool=EntrezSystem2.PEntrez.Pubmed.Pubmed_ResultsPanel.Pubmed_RVDocSum) (2005). Extraction and processing of high quality RNA from impalpable and macroscopically invisible prostate cancer for microarray gene expression analysis. *Internatinal Journal of Oncology* **27**: 713-720.

221. [Gober MD, Smith CC, Ueda K, Toretsky JA, Aurelian L.](http://www.ncbi.nlm.nih.gov/pubmed/12832417?ordinalpos=2&itool=EntrezSystem2.PEntrez.Pubmed.Pubmed_ResultsPanel.Pubmed_RVDocSum) (2003). Forced expression of the H11 heat shock protein can be regulated by DNA methylation and trigger apoptosis in human cells. *Journal of Biological Chemistry* **278**: 37600-37609.

222. [Yu YX, Heller A, Liehr T, Smith CC, Aurelian L.](http://www.ncbi.nlm.nih.gov/pubmed/11295034?ordinalpos=3&itool=EntrezSystem2.PEntrez.Pubmed.Pubmed_ResultsPanel.Pubmed_RVDocSum) (2001). Expression analysis and chromosome location of a novel gene (H11) associated with the growth of human melanoma cells. *International Journal of Oncology* **18**: 905-911.

**Gene 73: TUBA3**

**Official symbol:** TUBA1A

**Official Full Name:** tubulin, alpha 1a

**Also known as:** LIS3; TUBA3; FLJ25113; B-ALPHA-1

**References:**

**Gene 74: LIM**

**Official symbol:** PDLIM5

**Official Full Name:** PDZ and LIM domain 5

**Also known as:** L9; ENH; LIM; ENH1

**References:**

223. [Eeckhoute J, Carroll JS, Geistlinger TR, Torres-Arzayus MI, Brown M.](http://www.ncbi.nlm.nih.gov/pubmed/16980581?ordinalpos=1&itool=EntrezSystem2.PEntrez.Pubmed.Pubmed_ResultsPanel.Pubmed_RVDocSum) (2006). A cell-type-specific transcriptional network required for estrogen regulation of cyclin D1 and cell cycle progression in breast cancer. *Genes & Development* **20**: 2513-2526.

224. [Lasorella A, Iavarone A.](http://www.ncbi.nlm.nih.gov/pubmed/16549780?ordinalpos=2&itool=EntrezSystem2.PEntrez.Pubmed.Pubmed_ResultsPanel.Pubmed_RVDocSum) (2006). The protein ENH is a cytoplasmic sequestration factor for Id2 in normal and tumor cells from the nervous system. *Proceedings of the National Academy of Sciences of the United States of America* **103**: 4976-4981.

**Gene 75: LPP**

**Official symbol: LPP**

**Official Full Name:** LIM domain containing preferred translocation partner in lipoma

**Also known as:**

**References:**

225. [Guo B, Sallis RE, Greenall A, Petit MM, Jansen E, Young L, Van de Ven WJ, Sharrocks AD.](http://www.ncbi.nlm.nih.gov/pubmed/16738319?ordinalpos=2&itool=EntrezSystem2.PEntrez.Pubmed.Pubmed_ResultsPanel.Pubmed_RVDocSum) (2006). The LIM domain protein LPP is a coactivator for the ETS domain transcription factor PEA3. *Molecular and Cellular Biology* **26**: 4529-4538.

226. [Majesky MW.](http://www.ncbi.nlm.nih.gov/pubmed/16484626?ordinalpos=3&itool=EntrezSystem2.PEntrez.Pubmed.Pubmed_ResultsPanel.Pubmed_RVDocSum) (2006). Organizing motility: LIM domains, LPP, and smooth muscle migration. *Circulation Research* **98**: 306-308.

227. [Crombez KR, Vanoirbeek EM, Van de Ven WJ, Petit MM.](http://www.ncbi.nlm.nih.gov/pubmed/15755872?ordinalpos=6&itool=EntrezSystem2.PEntrez.Pubmed.Pubmed_ResultsPanel.Pubmed_RVDocSum) (2005). Transactivation functions of the tumor-specific HMGA2/LPP fusion protein are augmented by wild-type HMGA2. *Molecular Cancer Research* **3**: 63-70.

228. [Petit MM, Mols R, Schoenmakers EF, Mandahl N, Van de Ven WJ.](http://www.ncbi.nlm.nih.gov/pubmed/8812423?ordinalpos=10&itool=EntrezSystem2.PEntrez.Pubmed.Pubmed_ResultsPanel.Pubmed_RVDocSum) (1996). LPP, the preferred fusion partner gene of HMGIC in lipomas, is a novel member of the LIM protein gene family. *Genomics* **36**: 118-129.

**Gene 76: MAD2L1BP**

**Official symbol:** MAD2L1BP

**Official Full Name:** MAD2L1 binding protein

**Also known as:** CMT2; KIAA0110; MGC11282; RP1-261G23.6

**References:**

229. [Yun MY, Kim SB, Park S, Han CJ, Han YH, Yoon SH, Kim SH, Kim CM, Choi DW, Cho MH, Park GH, Lee KH.](http://www.ncbi.nlm.nih.gov/pubmed/17934339?ordinalpos=2&itool=EntrezSystem2.PEntrez.Pubmed.Pubmed_ResultsPanel.Pubmed_RVDocSum) (2007). Mutation analysis of p31comet gene, a negative regulator of Mad2, in human hepatocellular carcinoma. *Experimental & molecular medicine* **39**: 508-513.

**Gene 77: ADAMTS1**

**Official symbol: ADAMTS1**

**Official Full Name:** ADAM metallopeptidase with thrombospondin type 1 motif, 1

**Also known as:** C3-C5; METH1; KIAA1346

**References:**

230. [Gustavsson H, Jennbacken K, Welén K, Damber JE.](http://www.ncbi.nlm.nih.gov/pubmed/18076023?ordinalpos=1&itool=EntrezSystem2.PEntrez.Pubmed.Pubmed_ResultsPanel.Pubmed_RVDocSum) (2008). Altered expression of genes regulating angiogenesis in experimental androgen-independent prostate cancer. *Prostate* **68**: 161-170.

231. [Lind GE, Kleivi K, Meling GI, Teixeira MR, Thiis-Evensen E, Rognum TO, Lothe RA.](http://www.ncbi.nlm.nih.gov/pubmed/17167179?ordinalpos=2&itool=EntrezSystem2.PEntrez.Pubmed.Pubmed_ResultsPanel.Pubmed_RVDocSum) (2006). ADAMTS1, CRABP1, and NR3C1 identified as epigenetically deregulated genes in colorectal tumorigenesis. *Cellular Oncology* **28**: 259-272.

232. [Cross NA, Chandrasekharan S, Jokonya N, Fowles A, Hamdy FC, Buttle DJ, Eaton CL.](http://www.ncbi.nlm.nih.gov/pubmed/15599946?ordinalpos=3&itool=EntrezSystem2.PEntrez.Pubmed.Pubmed_ResultsPanel.Pubmed_RVDocSum) (2005). The expression and regulation of ADAMTS-1, -4, -5, -9, and -15, and TIMP-3 by TGFbeta1 in prostate cells: relevance to the accumulation of versican. *Prostate* **63**: 269-275.

**Gene 78: ARHA**

**Official symbol:** RHOA

**Official Full Name:** ras homolog gene family, member A

**Also known as:** ARHA; ARH12; RHO12; RHOH12

**References:**

233. [Nie D, Guo Y, Yang D, Tang Y, Chen Y, Wang MT, Zacharek A, Qiao Y, Che M, Honn KV.](http://www.ncbi.nlm.nih.gov/pubmed/18172303?ordinalpos=2&itool=EntrezSystem2.PEntrez.Pubmed.Pubmed_ResultsPanel.Pubmed_RVDocSum) (2008). Thromboxane A2 receptors in prostate carcinoma: expression and its role in regulating cell motility via small GTPase Rho. *Cancer Research* **68**: 115-121.

234. [Evelyn CR, Wade SM, Wang Q, Wu M, Iñiguez-Lluhí JA, Merajver SD, Neubig RR.](http://www.ncbi.nlm.nih.gov/pubmed/17699722?ordinalpos=4&itool=EntrezSystem2.PEntrez.Pubmed.Pubmed_ResultsPanel.Pubmed_RVDocSum) (2007). CCG-1423: a small-molecule inhibitor of RhoA transcriptional signaling. *Molecular Cancer Therapeutics* **6**: 2249-2260.

235. [Zheng R, Iwase A, Shen R, Goodman OB Jr, Sugimoto N, Takuwa Y, Lerner DJ, Nanus DM.](http://www.ncbi.nlm.nih.gov/pubmed/16652149?ordinalpos=11&itool=EntrezSystem2.PEntrez.Pubmed.Pubmed_ResultsPanel.Pubmed_RVDocSum) (2006). Neuropeptide-stimulated cell migration in prostate cancer cells is mediated by RhoA kinase signaling and inhibited by neutral endopeptidase. *Oncogene* 25: 5942-5952.

236. [Gao Y, Dickerson JB, Guo F, Zheng J, Zheng Y.](http://www.ncbi.nlm.nih.gov/pubmed/15128949?ordinalpos=24&itool=EntrezSystem2.PEntrez.Pubmed.Pubmed_ResultsPanel.Pubmed_RVDocSum) (2004). Rational design and characterization of a Rac GTPase-specific small molecule inhibitor. *Proceedings of the National Academy of Sciences of the United States of America* **101**: 7618-7623.

237. [Ghosh PM, Ghosh-Choudhury N, Moyer ML, Mott GE, Thomas CA, Foster BA, Greenberg NM, Kreisberg JI.](http://www.ncbi.nlm.nih.gov/pubmed/10435593?ordinalpos=33&itool=EntrezSystem2.PEntrez.Pubmed.Pubmed_ResultsPanel.Pubmed_RVDocSum) (1999). Role of RhoA activation in the growth and morphology of a murine prostate tumor cell line. *Oncogene* **18**: 4120-4130.

**Gene 79: TXNIP**

**Official symbol:** TXNIP

**Official Full Name:** thioredoxin interacting protein

**Also known as:** THIF; VDUP1; HHCPA78; EST01027

**References:**

238. [Dunn TA, Chen S, Faith DA, Hicks JL, Platz EA, Chen Y, Ewing CM, Sauvageot J, Isaacs WB, De Marzo AM, Luo J.](http://www.ncbi.nlm.nih.gov/pubmed/17071605?ordinalpos=1&itool=EntrezSystem2.PEntrez.Pubmed.Pubmed_ResultsPanel.Pubmed_RVDocSum) (2006). A novel role of myosin VI in human prostate cancer. *American Journal of Pathology* **169**: 1843-1854.

239. [Xu W, Ngo L, Perez G, Dokmanovic M, Marks PA.](http://www.ncbi.nlm.nih.gov/pubmed/17030815?ordinalpos=2&itool=EntrezSystem2.PEntrez.Pubmed.Pubmed_ResultsPanel.Pubmed_RVDocSum) (2006). Intrinsic apoptotic and thioredoxin pathways in human prostate cancer cell response to histone deacetylase inhibitor. *Proceedings of the National Academy of Sciences of the United States of America* **103**: 15540-15545.

240. [Sheth SS, Bodnar JS, Ghazalpour A, Thipphavong CK, Tsutsumi S, Tward AD, Demant P, Kodama T, Aburatani H, Lusis AJ.](http://www.ncbi.nlm.nih.gov/pubmed/16607285?ordinalpos=17&itool=EntrezSystem2.PEntrez.Pubmed.Pubmed_ResultsPanel.Pubmed_RVDocSum) (2006). Hepatocellular carcinoma in Txnip-deficient mice. *Oncogene* **25**: 3528-3536.

241. [Goldberg SF, Miele ME, Hatta N, Takata M, Paquette-Straub C, Freedman LP, Welch DR.](http://www.ncbi.nlm.nih.gov/pubmed/12543799?ordinalpos=36&itool=EntrezSystem2.PEntrez.Pubmed.Pubmed_ResultsPanel.Pubmed_RVDocSum) (2003). Melanoma metastasis suppression by chromosome 6: evidence for a pathway regulated by CRSP3 and TXNIP. *Cancer Research* **63**: 432-440.

**Gene 80: OGDH**

**Official symbol: OGDH**

**Official Full Name:** oxoglutarate (alpha-ketoglutarate) dehydrogenase (lipoamide)

**Also known as:** E1k; OGDC; AKGDH

**References:**

**Gene 81: RPL35**

**Official symbol: RPL35**

**Official Full Name:** ribosomal protein L35

**Also known as:**

**References:**

**Gene 82: ANKH**

**Official symbol: ANKN**

**Official Full Name:** ankylosis, progressive homolog (mouse)

**Also known as:** ANK; CMDJ; HANK; MANK; CCAL2; CPPDD; FLJ27166

**References:**

242. [Kloth JN, Oosting J, van Wezel T, Szuhai K, Knijnenburg J, Gorter A, Kenter GG, Fleuren GJ, Jordanova ES.](http://www.ncbi.nlm.nih.gov/pubmed/17311676?ordinalpos=1&itool=EntrezSystem2.PEntrez.Pubmed.Pubmed_ResultsPanel.Pubmed_RVDocSum) (2007). Combined array-comparative genomic hybridization and single-nucleotide polymorphism-loss of heterozygosity analysis reveals complex genetic alterations in cervical cancer. *BMC Genomics* **8**: 53.

243. [Sobel RE, Wang Y, Sadar MD.](http://www.ncbi.nlm.nih.gov/pubmed/16618209?ordinalpos=2&itool=EntrezSystem2.PEntrez.Pubmed.Pubmed_ResultsPanel.Pubmed_RVDocSum) Molecular analysis and characterization of PrEC, commercially available prostate epithelial cells. (2006). *In Vitro Cellular & Developmental Biology Animal* **42**: 33-39.

244. [Coe BP, Henderson LJ, Garnis C, Tsao MS, Gazdar AF, Minna J, Lam S, Macaulay C, Lam WL.](http://www.ncbi.nlm.nih.gov/pubmed/15611929?ordinalpos=3&itool=EntrezSystem2.PEntrez.Pubmed.Pubmed_ResultsPanel.Pubmed_RVDocSum) (2005). High-resolution chromosome arm 5p array CGH analysis of small cell lung carcinoma cell lines. *Genes, Chromosomes & Cancer* **42**: 308-313.

245. [Zheng M, Simon R, Mirlacher M, Maurer R, Gasser T, Forster T, Diener PA, Mihatsch MJ, Sauter G, Schraml P.](http://www.ncbi.nlm.nih.gov/pubmed/15215162?ordinalpos=4&itool=EntrezSystem2.PEntrez.Pubmed.Pubmed_ResultsPanel.Pubmed_RVDocSum) (2004). TRIO amplification and abundant mRNA expression is associated with invasive tumor growth and rapid tumor cell proliferation in urinary bladder cancer. *American journal of pathology* **165**: 63-69.

246. [Dhanasekaran SM, Dash A, Yu J, Maine IP, Laxman B, Tomlins SA, Creighton CJ, Menon A, Rubin MA, Chinnaiyan AM.](http://www.ncbi.nlm.nih.gov/pubmed/15548588?ordinalpos=2&itool=EntrezSystem2.PEntrez.Pubmed.Pubmed_ResultsPanel.Pubmed_RVDocSum) (2005). Molecular profiling of human prostate tissues: insights into gene expression patterns of prostate development during puberty. *FASEB Journal* **19**: 243-245.

**Gene 83: MPST**

**Official symbol: MPST**

**Official Full Name:** mercaptopyruvate sulfurtransferase

**Also known as:** MST; TST2; MGC24539

**References:**

247. [Iciek M, Włodek L.](http://www.ncbi.nlm.nih.gov/pubmed/11785922?ordinalpos=1&itool=EntrezSystem2.PEntrez.Pubmed.Pubmed_ResultsPanel.Pubmed_RVDocSum) (2201). Biosynthesis and biological properties of compounds containing highly reactive, reduced sulfane sulfur. [*Polish journal of pharmacology*](http://www.ncbi.nlm.nih.gov/sites/entrez?Db=journals&Cmd=ShowDetailView&TermToSearch=2464) **53**: 215-225.

248. [Faucher F, Lacoste L, Dufort I, Luu-The V.](http://www.ncbi.nlm.nih.gov/pubmed/11358677?ordinalpos=2&itool=EntrezSystem2.PEntrez.Pubmed.Pubmed_ResultsPanel.Pubmed_RVDocSum) (2001). High metabolization of catecholestrogens by type 1 estrogen sulfotransferase (hEST1). *Journal of Steroid Biochemistry and Molecular Biology* **77**: 83-86.

249. [Włodek L, Wróbel M, Czubak J.](http://www.ncbi.nlm.nih.gov/pubmed/8094343?ordinalpos=4&itool=EntrezSystem2.PEntrez.Pubmed.Pubmed_ResultsPanel.Pubmed_RVDocSum) (1993). Transamination and transsulphuration of L-cysteine in Ehrlich ascites tumor cells and mouse liver. The nonenzymatic reaction of L-cysteine with pyruvate. *International journal of biochemistry* **25**: 107-112.

**Gene 84: MORF4L2**

**Official symbol:** MORF4L2

**Official Full Name:** mortality factor 4 like 2

**Also known as:** MRGX; MORFL2; KIAA0026

**References:**

## 250. [**Shadeo A**](http://www.ncbi.nlm.nih.gov/sites/entrez?Db=pubmed&Cmd=Search&Term="Shadeo A"%5BAuthor%5D&itool=EntrezSystem2.PEntrez.Pubmed.Pubmed_ResultsPanel.Pubmed_RVAbstractPlusDrugs1), [**Chari R**](http://www.ncbi.nlm.nih.gov/sites/entrez?Db=pubmed&Cmd=Search&Term="Chari R"%5BAuthor%5D&itool=EntrezSystem2.PEntrez.Pubmed.Pubmed_ResultsPanel.Pubmed_RVAbstractPlusDrugs1), [**Lonergan KM**](http://www.ncbi.nlm.nih.gov/sites/entrez?Db=pubmed&Cmd=Search&Term="Lonergan KM"%5BAuthor%5D&itool=EntrezSystem2.PEntrez.Pubmed.Pubmed_ResultsPanel.Pubmed_RVAbstractPlusDrugs1), [**Pusic A**](http://www.ncbi.nlm.nih.gov/sites/entrez?Db=pubmed&Cmd=Search&Term="Pusic A"%5BAuthor%5D&itool=EntrezSystem2.PEntrez.Pubmed.Pubmed_ResultsPanel.Pubmed_RVAbstractPlusDrugs1), [**Miller D**](http://www.ncbi.nlm.nih.gov/sites/entrez?Db=pubmed&Cmd=Search&Term="Miller D"%5BAuthor%5D&itool=EntrezSystem2.PEntrez.Pubmed.Pubmed_ResultsPanel.Pubmed_RVAbstractPlusDrugs1), [**Ehlen T**](http://www.ncbi.nlm.nih.gov/sites/entrez?Db=pubmed&Cmd=Search&Term="Ehlen T"%5BAuthor%5D&itool=EntrezSystem2.PEntrez.Pubmed.Pubmed_ResultsPanel.Pubmed_RVAbstractPlusDrugs1), [**Van Niekerk D**](http://www.ncbi.nlm.nih.gov/sites/entrez?Db=pubmed&Cmd=Search&Term="Van Niekerk D"%5BAuthor%5D&itool=EntrezSystem2.PEntrez.Pubmed.Pubmed_ResultsPanel.Pubmed_RVAbstractPlusDrugs1), [**Matisic J**](http://www.ncbi.nlm.nih.gov/sites/entrez?Db=pubmed&Cmd=Search&Term="Matisic J"%5BAuthor%5D&itool=EntrezSystem2.PEntrez.Pubmed.Pubmed_ResultsPanel.Pubmed_RVAbstractPlusDrugs1), [**Richards-Kortum R**](http://www.ncbi.nlm.nih.gov/sites/entrez?Db=pubmed&Cmd=Search&Term="Richards-Kortum R"%5BAuthor%5D&itool=EntrezSystem2.PEntrez.Pubmed.Pubmed_ResultsPanel.Pubmed_RVAbstractPlusDrugs1), [**Follen M**](http://www.ncbi.nlm.nih.gov/sites/entrez?Db=pubmed&Cmd=Search&Term="Follen M"%5BAuthor%5D&itool=EntrezSystem2.PEntrez.Pubmed.Pubmed_ResultsPanel.Pubmed_RVAbstractPlusDrugs1), [**Guillaud M**](http://www.ncbi.nlm.nih.gov/sites/entrez?Db=pubmed&Cmd=Search&Term="Guillaud M"%5BAuthor%5D&itool=EntrezSystem2.PEntrez.Pubmed.Pubmed_ResultsPanel.Pubmed_RVAbstractPlusDrugs1), [**Lam WL**](http://www.ncbi.nlm.nih.gov/sites/entrez?Db=pubmed&Cmd=Search&Term="Lam WL"%5BAuthor%5D&itool=EntrezSystem2.PEntrez.Pubmed.Pubmed_ResultsPanel.Pubmed_RVAbstractPlusDrugs1), [**Macaulay C**](http://www.ncbi.nlm.nih.gov/sites/entrez?Db=pubmed&Cmd=Search&Term="Macaulay C"%5BAuthor%5D&itool=EntrezSystem2.PEntrez.Pubmed.Pubmed_ResultsPanel.Pubmed_RVAbstractPlusDrugs1). (2008). Up regulation in gene expression of chromatin remodelling factors in cervical intraepithelial neoplasia. [***BMC Genomics***](javascript:AL_get(this, 'jour', 'BMC Genomics.');) 9**: 64 Epub ahead of print**

**Gene 85: DKFZP434B044**

**Official symbol:** CRISPLD2

**Official Full Name:** cysteine-rich secretory protein LCCL domain containing 2

**Also known as:** CRISP11; LCRISP2; MGC74865; DKFZP434B044

**References:**

**Gene 86: CD9**

**Official symbol: CD9**

**Official Full Name:** CD9 molecule

**Also known as:** 5H9; BA2; P24; GIG2; MIC3; MRP-1; BTCC-1; DRAP-27; TSPAN29

**References:**

251. [Zvereff V, Wang JC, Shun K, Lacoste J, Chevrette M.](http://www.ncbi.nlm.nih.gov/pubmed/17848953?ordinalpos=1&itool=EntrezSystem2.PEntrez.Pubmed.Pubmed_ResultsPanel.Pubmed_RVDocSum) (2007). Colocalisation of CD9 and mortalin in CD9-induced mitotic catastrophe in human prostate cancer cells. *British Journal of Cancer* **97**: 941-948.

252. [Wang JC, Bégin LR, Bérubé NG, Chevalier S, Aprikian AG, Gourdeau H, Chevrette M.](http://www.ncbi.nlm.nih.gov/pubmed/17406028?ordinalpos=2&itool=EntrezSystem2.PEntrez.Pubmed.Pubmed_ResultsPanel.Pubmed_RVDocSum) (2007). Down-regulation of CD9 expression during prostate carcinoma progression is associated with CD9 mRNA modifications. *Clinical Cancer Research* **13**: 2354-2361.

253. [He B, Liu L, Cook GA, Grgurevich S, Jennings LK, Zhang XA.](http://www.ncbi.nlm.nih.gov/pubmed/15557282?ordinalpos=5&itool=EntrezSystem2.PEntrez.Pubmed.Pubmed_ResultsPanel.Pubmed_RVDocSum) (2005). Tetraspanin CD82 attenuates cellular morphogenesis through down-regulating integrin alpha6-mediated cell adhesion. *Journal of biological chemistry* **280**: 3346-3354.

254. [Zhang XA, Lane WS, Charrin S, Rubinstein E, Liu L.](http://www.ncbi.nlm.nih.gov/pubmed/12750295?ordinalpos=6&itool=EntrezSystem2.PEntrez.Pubmed.Pubmed_ResultsPanel.Pubmed_RVDocSum) (2003). EWI2/PGRL associates with the metastasis suppressor KAI1/CD82 and inhibits the migration of prostate cancer cells. *Cancer Research* **63:** 2665-2674

255. [White A, Lamb PW, Barrett JC.](http://www.ncbi.nlm.nih.gov/pubmed/9671393?ordinalpos=7&itool=EntrezSystem2.PEntrez.Pubmed.Pubmed_ResultsPanel.Pubmed_RVDocSum) (1998). Frequent downregulation of the KAI1(CD82) metastasis suppressor protein in human cancer cell lines. *Oncogene* **16**: 3143-3149.

**Gene 87: ALDH3A2**

**Official symbol:** ALDH3A2

**Official Full Name:** aldehyde dehydrogenase 3 family, member A2

**Also known as:** SLS; FALDH; ALDH10; FLJ20851; DKFZp686E23276

**References:**

**Gene 88: SCN2B**

**Official symbol:** SCN2B

**Official Full Name:** sodium channel, voltage-gated, type II, beta

**Also known as:**

**References:**

256. [Pertin M](http://www.ncbi.nlm.nih.gov/sites/entrez?Db=pubmed&Cmd=Search&Term="Pertin M"%5BAuthor%5D&itool=EntrezSystem2.PEntrez.Pubmed.Pubmed_ResultsPanel.Pubmed_RVAbstractPlusDrugs1), [Ji RR](http://www.ncbi.nlm.nih.gov/sites/entrez?Db=pubmed&Cmd=Search&Term="Ji RR"%5BAuthor%5D&itool=EntrezSystem2.PEntrez.Pubmed.Pubmed_ResultsPanel.Pubmed_RVAbstractPlusDrugs1), [Berta T](http://www.ncbi.nlm.nih.gov/sites/entrez?Db=pubmed&Cmd=Search&Term="Berta T"%5BAuthor%5D&itool=EntrezSystem2.PEntrez.Pubmed.Pubmed_ResultsPanel.Pubmed_RVAbstractPlusDrugs1), [Powell AJ](http://www.ncbi.nlm.nih.gov/sites/entrez?Db=pubmed&Cmd=Search&Term="Powell AJ"%5BAuthor%5D&itool=EntrezSystem2.PEntrez.Pubmed.Pubmed_ResultsPanel.Pubmed_RVAbstractPlusDrugs1), [Karchewski L](http://www.ncbi.nlm.nih.gov/sites/entrez?Db=pubmed&Cmd=Search&Term="Karchewski L"%5BAuthor%5D&itool=EntrezSystem2.PEntrez.Pubmed.Pubmed_ResultsPanel.Pubmed_RVAbstractPlusDrugs1), [Tate SN](http://www.ncbi.nlm.nih.gov/sites/entrez?Db=pubmed&Cmd=Search&Term="Tate SN"%5BAuthor%5D&itool=EntrezSystem2.PEntrez.Pubmed.Pubmed_ResultsPanel.Pubmed_RVAbstractPlusDrugs1), [Isom LL](http://www.ncbi.nlm.nih.gov/sites/entrez?Db=pubmed&Cmd=Search&Term="Isom LL"%5BAuthor%5D&itool=EntrezSystem2.PEntrez.Pubmed.Pubmed_ResultsPanel.Pubmed_RVAbstractPlusDrugs1), [Woolf CJ](http://www.ncbi.nlm.nih.gov/sites/entrez?Db=pubmed&Cmd=Search&Term="Woolf CJ"%5BAuthor%5D&itool=EntrezSystem2.PEntrez.Pubmed.Pubmed_ResultsPanel.Pubmed_RVAbstractPlusDrugs1), [Gilliard N](http://www.ncbi.nlm.nih.gov/sites/entrez?Db=pubmed&Cmd=Search&Term="Gilliard N"%5BAuthor%5D&itool=EntrezSystem2.PEntrez.Pubmed.Pubmed_ResultsPanel.Pubmed_RVAbstractPlusDrugs1), [Spahn DR](http://www.ncbi.nlm.nih.gov/sites/entrez?Db=pubmed&Cmd=Search&Term="Spahn DR"%5BAuthor%5D&itool=EntrezSystem2.PEntrez.Pubmed.Pubmed_ResultsPanel.Pubmed_RVAbstractPlusDrugs1), [Decosterd I](http://www.ncbi.nlm.nih.gov/sites/entrez?Db=pubmed&Cmd=Search&Term="Decosterd I"%5BAuthor%5D&itool=EntrezSystem2.PEntrez.Pubmed.Pubmed_ResultsPanel.Pubmed_RVAbstractPlusDrugs1). (2005). Upregulation of the voltage-gated sodium channel beta2 subunit in neuropathic pain models: characterization of expression in injured and non-injured primary sensory neurons. [*Journal of Neuroscience*](javascript:AL_get(this, 'jour', 'J Neurosci.');) **25**: 10970-10980.

**Gene 89: SPARCL1**

**Official symbol: SPARCL1**

**Official Full Name:** SPARC-like 1 (mast9, hevin)

**Also known as:** SC1; PIG33

**References:**

257. [Schlomm T, Luebke AM, Sültmann H, Hellwinkel OJ, Sauer U, Poustka A, David KA, Chun FK, Haese A, Graefen M, Erbersdobler A, Huland H.](http://www.ncbi.nlm.nih.gov/pubmed/16077921?ordinalpos=1&itool=EntrezSystem2.PEntrez.Pubmed.Pubmed_ResultsPanel.Pubmed_RVDocSum) (2005). Extraction and processing of high quality RNA from impalpable and macroscopically invisible prostate cancer for microarray gene expression analysis. *International Journal of Oncology* **27**: 713-720.

258. [Isler SG, Ludwig CU, Chiquet-Ehrismann R, Schenk S.](http://www.ncbi.nlm.nih.gov/pubmed/15375558?ordinalpos=2&itool=EntrezSystem2.PEntrez.Pubmed.Pubmed_ResultsPanel.Pubmed_RVDocSum) (2004). Evidence for transcriptional repression of SPARC-like 1, a gene downregulated in human lung tumors. *International Journal of Oncology* **25**: 1073-1079.

259. [Isler SG, Schenk S, Bendik I, Schraml P, Novotna H, Moch H, Sauter G, Ludwig CU.](http://www.ncbi.nlm.nih.gov/pubmed/11179481?ordinalpos=3&itool=EntrezSystem2.PEntrez.Pubmed.Pubmed_ResultsPanel.Pubmed_RVDocSum) (2001). Genomic organization and chromosomal mapping of SPARC-like 1, a gene down regulated in cancers. *International Journal of Oncology* **18**: 521-526.

260. [Nelson PS, Plymate SR, Wang K, True LD, Ware JL, Gan L, Liu AY, Hood L.](http://www.ncbi.nlm.nih.gov/pubmed/9443398?ordinalpos=4&itool=EntrezSystem2.PEntrez.Pubmed.Pubmed_ResultsPanel.Pubmed_RVDocSum) (1998). Hevin, an antiadhesive extracellular matrix protein, is down-regulated in metastatic prostate adenocarcinoma. *Cancer Research* **58**: 232-236.

**Gene 90: IGJ**

**Official symbol: IGJ**

**Official Full Name:** immunoglobulin J polypeptide, linker protein for immunoglobulin alpha and mu polypeptides

**Also known as:** JCH; IGCJ

**References:**

261. [Yao R, Rich SA, Schneider E.](http://www.ncbi.nlm.nih.gov/pubmed/12142393?ordinalpos=1&itool=EntrezSystem2.PEntrez.Pubmed.Pubmed_ResultsPanel.Pubmed_RVDocSum) (2002). Validation of sixteen leukemia and lymphoma cell lines as controls for molecular gene rearrangement assays. *Clinical Chemistry* **48**: 1344-1351.

262. [Tajima K, Fukase N, Shiono T, Katagiri T, Mito S.](http://www.ncbi.nlm.nih.gov/pubmed/7606090?ordinalpos=2&itool=EntrezSystem2.PEntrez.Pubmed.Pubmed_ResultsPanel.Pubmed_RVDocSum) (1995). An autopsied case of T-cell rich B-cell lymphoma with general involvement. *International Medicina* **34**: 243-246.

**Gene 91: ZNF134**

**Official symbol: ZNF134**

**Official Full Name:** zinc finger protein 134

**Also known as:** pHZ-15; MGC138499; MGC141970

**References:**

**Gene 92: MRPL43**

**Official symbol: MRPL43**

**Official Full Name:** mitochondrial ribosomal protein L43

**Also known as:** bMRP36a; MGC17989; MGC48892

**References:**

**Gene 93: LOC152485**

**Official symbol:**

**Official Full Name:** hypothetical protein LOC152485

**Also known as:** LOC152485

**References:**

**Gene 94: CALM2**

**Official symbol: CALM2**

**Official Full Name:** calmodulin 2 (phosphorylase kinase, delta)

**Also known as:** PHKD; CAMII; PHKD2

**References:**

263. [Rust R, Visser L, van der Leij J, Harms G, Blokzijl T, Deloulme JC, van der Vlies P, Kamps W, Kok K, Lim M, Poppema S, van den Berg A.](http://www.ncbi.nlm.nih.gov/pubmed/16351635?ordinalpos=1&itool=EntrezSystem2.PEntrez.Pubmed.Pubmed_ResultsPanel.Pubmed_RVDocSum) (2005). High expression of calcium-binding proteins, S100A10, S100A11 and CALM2 in anaplastic large cell lymphoma. *British Journal of Haematology* **131**: 596-608.

264. [Toutenhoofd SL, Strehler EE.](http://www.ncbi.nlm.nih.gov/pubmed/12445464?ordinalpos=2&itool=EntrezSystem2.PEntrez.Pubmed.Pubmed_ResultsPanel.Pubmed_RVDocSum) (2002). Regulation of calmodulin mRNAs in differentiating human IMR-32 neuroblastoma cells. *Biochimica et Biophysica Acta* **1600**: 95-104.

265. [Toutenhoofd SL, Foletti D, Wicki R, Rhyner JA, Garcia F, Tolon R, Strehler EE.](http://www.ncbi.nlm.nih.gov/pubmed/9681195?ordinalpos=3&itool=EntrezSystem2.PEntrez.Pubmed.Pubmed_ResultsPanel.Pubmed_RVDocSum) (1998). Characterization of the human CALM2 calmodulin gene and comparison of the transcriptional activity of CALM1, CALM2 and CALM3. *Cell Calcium* **23**: 323-338.

**Gene 95: COL9A2**

**Official symbol: COL9A2**

**Official Full Name:** collagen, type IX, alpha 2

**Also known as:** MED; EDM2; DJ39G22.4

**References:**

**Gene 96: GAGEC1**

**Official symbol:** PAGE4

**Official Full Name:** P antigen family, member 4 (prostate associated)

**Also known as:** JM27; GAGE-9; GAGEC1; PAGE-1; PAGE-4; FLJ35184

**References:**

266. [Yokokawa J, Bera TK, Palena C, Cereda V, Remondo C, Gulley JL, Arlen PM, Pastan I, Schlom J, Tsang KY.](http://www.ncbi.nlm.nih.gov/pubmed/17397028?ordinalpos=1&itool=EntrezSystem2.PEntrez.Pubmed.Pubmed_ResultsPanel.Pubmed_RVDocSum) (2007). Identification of cytotoxic T-lymphocyte epitope(s) and its agonist epitope(s) of a novel target for vaccine therapy (PAGE4). *International Journal of Cancer* **121**: 595-605.

267. [Cannon GW, Mullins C, Lucia MS, Hayward SW, Lin V, Liu BC, Slawin K, Rubin MA, Getzenberg RH.](http://www.ncbi.nlm.nih.gov/pubmed/17222644?ordinalpos=2&itool=EntrezSystem2.PEntrez.Pubmed.Pubmed_ResultsPanel.Pubmed_RVDocSum) (2007). A preliminary study of JM-27: a serum marker that can specifically identify men with symptomatic benign prostatic hyperplasia. *Journal of Urology* **177**: 610-614.

268. [Sampson N, Untergasser G, Lilg C, Tadic L, Plas E, Berger P.](http://www.ncbi.nlm.nih.gov/pubmed/17113629?ordinalpos=3&itool=EntrezSystem2.PEntrez.Pubmed.Pubmed_ResultsPanel.Pubmed_RVDocSum) (2007). GAGEC1, a cancer/testis associated antigen family member, is a target of TGF-beta1 in age-related prostatic disease. *Mechanisms of Ageing and Development* **128**: 64-66.

269. [Iavarone C, Wolfgang C, Kumar V, Duray P, Willingham M, Pastan I, Bera TK.](http://www.ncbi.nlm.nih.gov/pubmed/12489849?ordinalpos=6&itool=EntrezSystem2.PEntrez.Pubmed.Pubmed_ResultsPanel.Pubmed_RVDocSum) (2002). PAGE4 is a cytoplasmic protein that is expressed in normal prostate and in prostate cancers. *Molecular Cancer Therapeutics* **1**: 329-335.

**Gene 97: CALM1**

**Official symbol: CALM1**

**Official Full Name:** calmodulin 1 (phosphorylase kinase, delta)

**Also known as:** CAMI; PHKD; DD132; CALML2

**References:**

270. [Flanagan JM, Popendikyte V, Pozdniakovaite N, Sobolev M, Assadzadeh A, Schumacher A, Zangeneh M, Lau L, Virtanen C, Wang SC, Petronis A.](http://www.ncbi.nlm.nih.gov/pubmed/16773567?ordinalpos=1&itool=EntrezSystem2.PEntrez.Pubmed.Pubmed_ResultsPanel.Pubmed_RVDocSum) (2006). Intra- and interindividual epigenetic variation in human germ cells. *American Journal of Human Genetics* **79**: 67-84.

2751 [Wang DY, McKague B, Liss SN, Edwards EA.](http://www.ncbi.nlm.nih.gov/pubmed/15597897?ordinalpos=2&itool=EntrezSystem2.PEntrez.Pubmed.Pubmed_ResultsPanel.Pubmed_RVDocSum) (2004). Gene expression profiles for detecting and distinguishing potential endocrine-disrupting compounds in environmental samples. *Environmental Science & Technology* **38**: 6396-6406.

272. [Toutenhoofd SL, Strehler EE.](http://www.ncbi.nlm.nih.gov/pubmed/12445464?ordinalpos=3&itool=EntrezSystem2.PEntrez.Pubmed.Pubmed_ResultsPanel.Pubmed_RVDocSum) (2002). Regulation of calmodulin mRNAs in differentiating human IMR-32 neuroblastoma cells. *Biochimica et Biophysica acta* **1600**: 95-104.

273. [Toutenhoofd SL, Foletti D, Wicki R, Rhyner JA, Garcia F, Tolon R, Strehler EE.](http://www.ncbi.nlm.nih.gov/pubmed/9681195?ordinalpos=4&itool=EntrezSystem2.PEntrez.Pubmed.Pubmed_ResultsPanel.Pubmed_RVDocSum) (1998). Characterization of the human CALM2 calmodulin gene and comparison of the transcriptional activity of CALM1, CALM2 and CALM3. *Cell Calcium* **23**: 323-338.

**Gene 98: ACTB**

**Official symbol: ACTB**

**Official Full Name:** actin, beta

**Also known as:** PS1TP5BP1

**References:**

274. [Ohl F, Jung M, Xu C, Stephan C, Rabien A, Burkhardt M, Nitsche A, Kristiansen G, Loening SA, Radonić A, Jung K.](http://www.ncbi.nlm.nih.gov/pubmed/16211407?ordinalpos=1&itool=EntrezSystem2.PEntrez.Pubmed.Pubmed_ResultsPanel.Pubmed_RVDocSum) (2005). Gene expression studies in prostate cancer tissue: which reference gene should be selected for normalization? *Journal of Molecular Medicine* **83**: 1014-1024.

275. [Yamashita S, Wakazono K, Nomoto T, Tsujino Y, Kuramoto T, Ushijima T.](http://www.ncbi.nlm.nih.gov/pubmed/16079240?ordinalpos=2&itool=EntrezSystem2.PEntrez.Pubmed.Pubmed_ResultsPanel.Pubmed_RVDocSum) (2005). Expression quantitative trait loci analysis of 13 genes in the rat prostate. *Genetics* **171**: 1231-1238.

276. [Zhou M, Tokumaru Y, Sidransky D, Epstein JI.](http://www.ncbi.nlm.nih.gov/pubmed/15126784?ordinalpos=3&itool=EntrezSystem2.PEntrez.Pubmed.Pubmed_ResultsPanel.Pubmed_RVDocSum) (2004). Quantitative GSTP1 methylation levels correlate with Gleason grade and tumor volume in prostate needle biopsies. *Journal of Urology* **171**: 2195-2198.

277. [Chaib H, Cockrell EK, Rubin MA, Macoska JA.](http://www.ncbi.nlm.nih.gov/pubmed/11326315?ordinalpos=4&itool=EntrezSystem2.PEntrez.Pubmed.Pubmed_ResultsPanel.Pubmed_RVDocSum) (2001). Profiling and verification of gene expression patterns in normal and malignant human prostate tissues by cDNA microarray analysis. *Neoplasia* **3**: 43-52.

**Gene 99: AGR2**

**Official symbol: AGR2**

**Official Full Name:** anterior gradient homolog 2 (Xenopus laevis)

**Also known as:** AG2; GOB-4; HAG-2; XAG-2

**References:**

278. [Wang Z, Hao Y, Lowe AW.](http://www.ncbi.nlm.nih.gov/pubmed/18199544?ordinalpos=1&itool=EntrezSystem2.PEntrez.Pubmed.Pubmed_ResultsPanel.Pubmed_RVDocSum) (2008). The adenocarcinoma-associated antigen, AGR2, promotes tumor growth, cell migration, and cellular transformation. *Cancer Research* **68**: 492-497.

279. [Zhang Y, Forootan SS, Liu D, Barraclough R, Foster CS, Rudland PS, Ke Y.](http://www.ncbi.nlm.nih.gov/pubmed/17457305?ordinalpos=2&itool=EntrezSystem2.PEntrez.Pubmed.Pubmed_ResultsPanel.Pubmed_RVDocSum) (2007). Increased expression of anterior gradient-2 is significantly associated with poor survival of prostate cancer patients. *Prostate Cancer and Prostatic Diseases* **10**: 293-300.

280. [Smirnov DA, Zweitzig DR, Foulk BW, Miller MC, Doyle GV, Pienta KJ, Meropol NJ, Weiner LM, Cohen SJ, Moreno JG, Connelly MC, Terstappen LW, O'Hara SM.](http://www.ncbi.nlm.nih.gov/pubmed/15958538?ordinalpos=4&itool=EntrezSystem2.PEntrez.Pubmed.Pubmed_ResultsPanel.Pubmed_RVDocSum) (2005). Global gene expression profiling of circulating tumor cells. *Cancer Research* **65**: 4993-4997.

281. [Zhang JS, Gong A, Cheville JC, Smith DI, Young CY.](http://www.ncbi.nlm.nih.gov/pubmed/15834940?ordinalpos=5&itool=EntrezSystem2.PEntrez.Pubmed.Pubmed_ResultsPanel.Pubmed_RVDocSum) (2005). AGR2, an androgen-inducible secretory protein overexpressed in prostate cancer. *Genes, Chromosomes & Cancer* **43**: 249-259.

282. [Kristiansen G, Pilarsky C, Wissmann C, Kaiser S, Bruemmendorf T, Roepcke S, Dahl E, Hinzmann B, Specht T, Pervan J, Stephan C, Loening S, Dietel M, Rosenthal A.](http://www.ncbi.nlm.nih.gov/pubmed/15532095?ordinalpos=6&itool=EntrezSystem2.PEntrez.Pubmed.Pubmed_ResultsPanel.Pubmed_RVDocSum) (2005). Expression profiling of microdissected matched prostate cancer samples reveals CD166/MEMD and CD24 as new prognostic markers for patient survival. Journal of Pathology 205: 359-376.

**Gene 100: RPS28**

**Official symbol:** RPS28

**Official Full Name:** ribosomal protein S28

**Also known as:**

**References:**
